# Associations of Adiposity, Circulating Protein Biomarkers, and Risk of Major Vascular Diseases

**DOI:** 10.1001/jamacardio.2020.6041

**Published:** 2020-12-02

**Authors:** Yuanjie Pang, Christiana Kartsonaki, Jun Lv, Zammy Fairhurst-Hunter, Iona Y. Millwood, Canqing Yu, Yu Guo, Yiping Chen, Zheng Bian, Ling Yang, Junshi Chen, Robert Clarke, Robin G. Walters, Michael V. Holmes, Liming Li, Zhengming Chen

**Affiliations:** 1 Department of Epidemiology and Biostatistics, School of Public Health, Peking University, Beijing, China; 2 Clinical Trial Service Unit & Epidemiological Studies Unit, Nuffield Department of Population Health, University of Oxford, United Kingdom; 3 Medical Research Council Population Health Research Unit at the University of Oxford, Nuffield Department of Population Health, University of Oxford, United Kingdom; 4 Chinese Academy of Medical Sciences, Beijing, China; 5 National Center for Food Safety Risk Assessment, Beijing, China; 6 National Institute for Health Research Oxford Biomedical Research Centre, Oxford University Hospital, Oxford, United Kingdom

## Abstract

**Importance:**

Obesity is associated with a higher risk of cardiovascular disease (CVD), but little is known about the role that circulating protein biomarkers play in this association.

**Objective:**

To examine the observational and genetic associations of adiposity with circulating protein biomarkers and the observational associations of proteins with incident CVD.

**Design, Setting, and Participants:**

This subcohort study included 628 participants from the prospective China Kadoorie Biobank who did not have a history of cancer at baseline. The Olink platform measured 92 protein markers in baseline plasma samples. Data were collected from June 2004 to January 2016 and analyzed from January 2019 to June 2020.

**Exposures:**

Measured body mass index (BMI) obtained during the baseline survey and genetically instrumented BMI derived using 571 externally weighted single-nucleotide variants.

**Main Outcomes and Measures:**

Cross-sectional associations of adiposity with biomarkers were examined using linear regression. Associations of biomarkers with CVD risk were assessed using Cox regression among those without prior cancer or CVD at baseline. Mendelian randomization was conducted to derive genetically estimated associations of BMI with biomarkers.

**Findings:**

In observational analyses of 628 individuals (mean [SD] age, 52.2 [10.5] years; 385 women [61.3%]), BMI (mean [SD], 23.9 [3.6]) was positively associated with 27 proteins (per 1-SD higher BMI; eg, interleukin-6: 0.21 [95% CI, 0.12-0.29] SD; interleukin-18: 0.13 [95% CI, 0.05-0.21] SD; monocyte chemoattractant protein–1: 0.12 [95% CI, 0.04-0.20] SD; hepatocyte growth factor: 0.31 [95% CI, 0.24-0.39] SD), and inversely with 3 proteins (Fas ligand: −0.11 [95% CI, −0.19 to −0.03] SD; TNF-related weak inducer of apoptosis, −0.14 [95% CI, −0.23 to −0.06] SD; and carbonic anhydrase 9: (−0.14 [95% CI, −0.22 to −0.05] SD), with similar associations identified for other adiposity traits (eg, waist circumference [*r* = 0.96]). In mendelian randomization, the associations of genetically elevated BMI with specific proteins were directionally consistent with the observational associations. In meta-analyses of genetically elevated BMI with 8 proteins, combining present estimates with previous studies, the most robust associations were shown for interleukin-6 (per 1-SD higher BMI; 0.21 [95% CI, 0.13-0.29] SD), interleukin-18 (0.16 [95% CI, 0.06-0.26] SD), monocyte chemoattractant protein–1 (0.21 [95% CI, 0.11-0.30] SD), monocyte chemotactic protein–3 (0.12 [95% CI, 0.03-0.21] SD), TNF-related apoptosis-inducing ligand (0.23 [95% CI, 0.13-0.32] SD), and hepatocyte growth factor (0.14 [95% CI, 0.06-0.22] SD). Of the 30 BMI-associated biomarkers, 10 (including interleukin-6, interleukin-18, and hepatocyte growth factor) were nominally associated with incident CVD.

**Conclusions and Relevance:**

Mendelian randomization shows adiposity to be associated with a range of protein biomarkers, with some biomarkers also showing association with CVD risk. Future studies are warranted to validate these findings and assess whether proteins may be mediators between adiposity and CVD.

## Key Points

**Question:**

Is adiposity associated with differences in circulating protein concentrations, and might these proteins potentially explain the associations of adiposity with risk of cardiovascular disease?

**Findings:**

In a cohort study of 628 individuals in China, there was evidence of genetic associations of body mass index with protein biomarkers consistent with observational associations, particularly for interleukin-6, interleukin-18, monocyte chemoattractant protein–1, monocyte chemotactic protein–3, TNF-related apoptosis-inducing ligand, and hepatocyte growth factor. Several of these proteins were observationally associated with risk of incident cardiovascular disease.

**Meaning:**

In this study of Chinese adults, adiposity was associated both cross-sectionally and through genetic analyses with a range of protein biomarkers, which might partly explain the association between adiposity and cardiovascular disease.

## Teaser

This subcohort study of Chinese adults examines the observational and genetic associations of adiposity with proteomics and the observational associations of adiposity with cardiovascular disease.

## Introduction

Adiposity is a major risk factor for cardiometabolic diseases and certain cancers.^1,2,3^ Inflammation, adipokine signaling, angiogenesis, and insulin resistance have all been proposed as possible mechanisms underlying these associations.^1,2,3^ For coronary heart disease (CHD), the central interleukin-6 (IL6) inflammatory signaling pathway plays an important role in atherogenesis and is also a drug target.^4^ Multiple cross-sectional studies have examined the associations of body mass index (BMI; calculated as weight in kilograms divided by height in meters squared) with inflammatory cytokines and growth factors and reported broadly positive associations of adiposity with interleukins, chemokines, and growth factors.^5,6,7,8,9,10,11,12,13,14,15^ Several randomized trials have reported that weight loss through dietary and exercise interventions reduces levels of interleukins (IL6, IL18, and IL1 receptor antagonist) and C-reactive protein (CRP),^16,17,18^ but there is limited evidence on other biomarkers.

Mendelian randomization can be used to evaluate the potential association of adiposity with levels of biomarkers.^19,20^ Previous mendelian randomization studies have suggested that BMI is associated with higher levels of CRP and IL6.^21,22,23,24^ However, the associations of adiposity with a range of protein biomarkers associated with cardiometabolic diseases and cancers has yet to be more fully characterized. Understanding the influence of adiposity on these biomarkers may help evaluate mediators and pathways between adiposity and diseases, leading to the discovery of novel therapeutic targets.

The objectives of this study were to examine the conventional observational and genetically estimated associations of adiposity with inflammation and immune-associated proteins in a subcohort of the China Kadoorie Biobank (CKB). We also evaluated the associations of these protein biomarkers with risk of CVD.

## Methods

### Study Population

**Figure 1.**
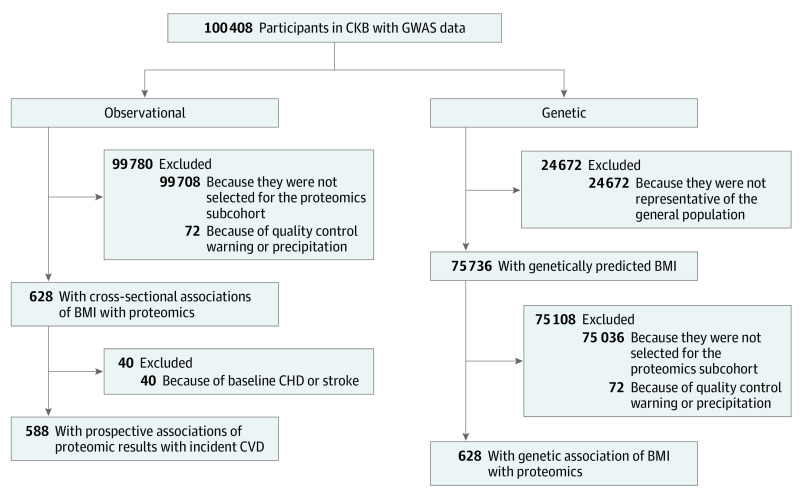
Flow Diagram A flow diagram to show participants whose data were used to estimate observational and genetic associations of body mass index, proteomics, and cardiovascular disease in the China Kadoorie Biobank (CKB). The excluded participants (n = 24 672) were enriched for cases as part of a case-control study, leaving 75 736 individuals with similar characteristics to the underlying CKB data set. BMI indicates body mass index; CHD, coronary heart disease; CVD, cardiovascular disease; GWAS indicates genome-wide association study.

The study population was a subcohort of CKB, which is a prospective cohort study of 512 715 adults aged 30 to 79 years, recruited between June 2004 and July 2008 from 10 geographically defined regions in China (5 urban and 5 rural regions). Details of the CKB design, survey methods, and long-term follow-up have been previously described.^25^ At the baseline survey, participants completed an interviewer-administered, laptop-based questionnaire, underwent a range of physical measurements, and provided a 10-mL nonfasting blood sample. A case-subcohort study was originally designed to examine the associations of proteomics with risk of incident pancreatic cancer, involving 700 cases of pancreatic cancer (*International Statistical Classification of Diseases and Related Health Problems, Tenth Revision* [*ICD-10*] code C25) that accumulated until January 1, 2016 (not considered in the present study), and a subcohort of 700 participants selected from the baseline cohort using simple random sampling with genome-wide genotyping data. In the subcohort, 72 participants were excluded for not passing quality control, leaving 628 participants for the present study. We tracked who among the present study participants developed incident vascular events (*ICD-10* codes I00-I09, I16-I25, I27-I88, I95-I99, and I10-I15 [only if fatal]) during 10 years of follow-up (Figure 1), including which of these vascular disease events were major adverse coronary events (*ICD-10* codes I21-I23, I60-I61, and I63-I64 [from any source]; and I00-I20, I24-I25, I27-I59, I62, I65-I88, and I95-I99 [only if fatal]). Greater than 90% diagnostic accuracy has been shown in ongoing outcome adjudication studies.

Prior international, national, and regional ethical approvals were obtained; this study was approved by the ethical committee and research council of the Chinese Center for Disease Control and Prevention and the Oxford Tropical Research Ethics Committee at the University of Oxford. All participants provided written informed consent.

### Proteomics Assay

The Olink Immuno-Oncology assay measured 92 protein biomarkers selected to include proteins known or suspected to be involved in promotion and inhibition of tumor immunity, chemotaxis, vascular and tissue remodeling, apoptosis and cell killing, and metabolism and autophagy (eTable 1 in the Supplement). The Olink method is based on proximity extension assay technology, to obtain normalized protein expression values, which is an arbitrary unit on a log2 scale.^26,27,28^ Details on the evaluation of assay performance, including derivation of the limit of detection, intra-assay and interassay coefficients of variation are described in eMethods and eFigures 19 and 20 in the Supplement, together with numerical values of the limit of detection, coefficients of variation, and grouping of the 92 proteins according to their main protein class and function (eTables 2 and 3 in the Supplement). We excluded IL21 and IL35 because more than 99% participants had values below the limits of detection, leaving 90 proteins for all analyses. In addition, 17 biomarkers were separately quantified using standard clinical biochemistry assays at the Wolfson Laboratory, Clinical Trial Service Unit, University of Oxford in the UK, in a nested case-control study of 18 181 participants and 17 biomarkers. Assessment of adiposity measures, other covariates, and clinical biochemistry are described in the eMethods in the Supplement. All biomarkers were standardized to have a SD of 1.

### Genotyping

Genotyping was conducted using a custom-designed 800K-SNP array (Axiom [Affymetrix]), with imputation to 1000 Genomes Phase 3. In CKB release 15, data were available for samples from 100 408 participants aged 30 to 79 years who had passed quality control (overall call rate, >99.97% across all variants), including a population-based sample of 75 736 participants randomly selected from the total CKB cohort. These 75 736 participants were used for genetic analyses in this study, and this included all participants in the proteomics subcohort. The remaining 24 672 participants who had been genotyped were selected for nested case-control studies of incident CVD or chronic obstructive pulmonary disease, and so, to avoid potential selection bias, they were not included in the analyses (Figure 1).

### Genetic Risk Score for BMI

We selected genetic variants as instrumental variables for BMI based on a meta-analysis of UK Biobank and the Genetic Investigation of Anthropometric Traits consortium (which examined 670 independent single-nucleotide variants [SNVs]; *r*^2^ ≤ 0.01 in European individuals).^29^ Eighty-four of these variants had low minor allele frequency (<1%) in CKB, leaving 586 SNVs for the BMI genetic score (eFigure 1 and eTable 4 in the Supplement). An externally weighted BMI genetic score was constructed by summing the number of effect alleles carried by each participant (the SD difference in BMI per effect allele), weighted by the reported effect size of each variant on BMI as reported by Biobank Japan.^30^ Fifteen of the 586 SNVs had low minor allele frequency (<1%) in Biobank Japan, leaving 571 SNVs for the weighted score. Five of the 571 SNVs were unavailable in Biobank Japan, and proxy SNPs were selected (*R*^2^ ≥ 0.80, using the linkage disequilibrium structure in CEU [1000 Genomes Project]). The BMI genetic score was a strong instrument (*F,* 1593; variance explained, 2.06%) and was not associated with traits that might be considered potential confounders (eTable 5 in the Supplement).

### Statistical Methods

In observational analysis involving 628 participants, linear regression was used to assess the associations of adiposity with protein markers, adjusted for age at baseline, age squared, sex, region, education, household income, alcohol use, self-rated health, systolic blood pressure, diabetes, statin treatment, prior kidney disease, and fasting time (ie, the time since last having eaten). For each biomarker, adjusted SD differences and 95% CIs associated with 1-SD higher adiposity calculated in the whole CKB cohort were estimated. In mendelian randomization analysis, we calculated the genetically estimated associations of BMI with proteomics by the 2-stage least squares estimator method using individual participant–level data. In the first stage, the associations between BMI genetic score and BMI were examined in 75 736 participants in the genome-wide association study population subset using linear regression, adjusting for age, age squared, sex, region, the first 12 principal components, education, smoking status, and alcohol use. In the second stage, the associations of the resulting estimated BMI values with proteomics were examined in the subcohort of 628 individuals using linear regression with the same adjustments. We calculated the genetically estimated associations per 3.4-point higher BMI (corresponding to 1-SD baseline BMI in the whole CKB cohort) on measured protein levels, to allow comparison with observational BMI. In prospective analyses of associations of protein levels with risk of CVD, Cox proportional hazards models were used to estimate hazard ratios (HRs) of vascular disease per 1-SD higher protein markers, adjusted for the same variables as in the analysis of adiposity and protein markers. We reported in the eMethods in the Supplement: (1) estimation of the extent to which additional adjustment for protein biomarkers might influence the observational association of BMI with CVD, (2) methods to account for multiple comparisons, (3) meta-analyses of the genetic associations of BMI with protein biomarkers and the observational associations of protein biomarkers with CVD, and (4) sensitivity analyses.

The statistical analysis was performed from January 2019 to June 2020 using R statistical software version 3.6.0 (R Project for Statistical Computing). We used the following approach to account for multiple comparisons. Significance was assessed at a 5% false-discovery rate (FDR) in the observational analysis of BMI with protein biomarkers. Unadjusted *P* values are reported for the genetic associations of BMI with protein biomarkers and observational associations of protein biomarkers with vascular events to avoid overcorrection.

## Results

The overall mean (SD) BMI of included participants was 23.9 (3.6). The mean (SD) age was 52.2 (10.5) years, and 385 (61.3%) were women. Participants with higher BMI had higher mean blood pressure (eg, mean [SD] systolic blood pressure: BMI <20, 120.2 [18.2] mm Hg vs BMI ≥27.5, 135.2 [23.7] mm Hg) and higher prevalence of diabetes (eg, BMI <20, 1 [1.3%] vs BMI ≥27.5, 7 [7.4%]) and less likely to be smokers (in men only; eg, BMI <20, 22 [73.3%] vs BMI ≥27.5, 20 [62.5%]) and physically active (eg, mean [SD] metabolic equivalent of task hours per day: BMI <20, 21.7 [16.2] vs BMI ≥27.5, 18.8 [13.4]) (Table). The patterns of baseline characteristics by BMI category in the subcohort were similar to those in the overall CKB cohort (eTable 6 in the Supplement).

**Table.** Baseline Characteristics of Participants in the Subcohort by Body Mass Index (BMI) Category

Variable^a^	BMI categories, mean (SD)					All (N = 628)
	<20 (n = 76)	20-<22.5 (n = 163)	22.5-<25.0 (n = 181)	25.0-<27.5 (n = 114)	≥27.5 (n = 94)	
Age, y	50.7 (11.1)	51.8 (10.8)	53.7 (11.1)	51.0 (9.2)	51.1 (9.8)	52.2 (10.5)
Female, No. (%)	46 (60.5)	97 (59.5)	111 (61.3)	65 (57.0)	66 (70.2)	385 (61.3)
Socioeconomic and lifestyle factors, No. (%)						
Urban region	28 (36.8)	69 (42.3)	98 (54.1)	67 (58.8)	61 (64.9)	323 (51.4)
≥9 y of Education	15 (19.7)	32 (19.6)	50 (27.6)	28 (24.6)	22 (23.4)	147 (23.4)
Household income ≥$5261/y^b^	15 (19.7)	27 (16.6)	23 (12.7)	27 (23.7)	24 (25.5)	116 (18.5)
Ever regular smoking, No. (%)						
Male	22 (73.3)	51 (79.7)	35 (50.7)	29 (60.4)	20 (62.5)	157 (64.6)
Female	3 (6.5)	2 (2.1)	2 (1.8)	1 (1.5)	5 (7.6)	13 (3.4)
Weekly drinking, No. (%)^c^						
Male	11 (36.7)	25 (39.1)	18 (26.1)	17 (35.4)	13 (40.6)	84 (34.6)
Female	0	0	2 (1.8)	2 (3.1)	4 (6.1)	8 (2.1)
Total physical activity, metabolic equivalent of task h/d	21.7 (16.2)	21.1 (15.0)	20.1 (13.6)	17.9 (14.2)	18.8 (13.4)	20.2 (14.4)
Blood pressure and anthropometry						
Systolic blood pressure, mm Hg	120.2 (18.2)	126.7 (22.8)	129.2 (18.8)	136.5 (22.0)	135.2 (23.7)	131.3 (21.9)
Random plasma glucose, mmol/L	5.8 (2.5)	5.5 (1.3)	6.0 (2.8)	6.1 (2.8)	5.9 (2.6)	6.0 (2.4)
BMI	18.8 (0.9)	21.3 (0.8)	23.6 (0.7)	25.9 (0.7)	28.6 (2.1)	23.9 (3.6)
Circumference, cm						
Waist	67.7 (5.0)	73.3 (4.9)	79.1 (6.1)	85.8 (5.2)	91.0 (8.3)	80.2 (10.5)
Hip	83.0 (3.8)	86.7 (4.4)	89.8 (4.1)	93.8 (4.8)	96.3 (6.1)	90.9 (7.3)
Waist-to-hip ratio	0.82 (0.05)	0.85 (0.06)	0.88 (0.06)	0.91 (0.06)	0.91 (0.07)	0.88 (0.07)
Prior disease history, No. (%)						
Coronary heart disease	1 (1.3)	2 (1.2)	11 (6.1)	7 (6.1)	6 (6.4)	27 (4.3)
Stroke or transient ischemic attack	1 (1.3)	2 (1.2)	4 (2.2)	2 (1.8)	7 (7.4)	16 (2.5)
Hypertension	2 (2.6)	9 (5.5)	13 (7.2)	19 (16.7)	23 (24.5)	66 (10.5)
Diabetes	1 (1.3)	2 (1.2)	6 (3.3)	5 (4.4)	7 (7.4)	21 (3.3)
Family history						
Diabetes	4 (5.3)	6 (3.7)	8 (4.4)	11 (9.6)	4 (4.3)	33 (5.3)
Cancer	10 (13.2)	26 (16.0)	23 (12.7)	20 (17.5)	17 (18.1)	96 (15.3)

Abbreviation: BMI, body mass index (calculated as weight in kilograms divided by height in meters squared).

^a^ All means by BMI categories are adjusted for age, sex, and region, except for age (which was only adjusted for sex and region). The numbers, percentages, and SDs are unadjusted values.

^b^ This is 35 000 or more Chinese yuan (renminbi) per year.

^c^ The numbers of male participants by BMI categories (<20, 20-<22.5, 22.5-<25, 25-<27.5, and ≥27.5) were 30, 64, 69, 48, and 32, respectively; numbers of female participants by BMI categories were 46, 97, 111, 65, and 66, respectively.

Of the 628 participants, 588 had no prior history of CVD at baseline. One hundred fifty participants developed incident vascular events, of which 60% were major adverse cardiovascular events.

For the 90 protein markers, most individuals had a near-normal distribution after log transformation, while the distributions of a few protein biomarkers were somewhat skewed to the right (eg, IL5, IL6, and vascular endothelial growth factor C) or skewed to the left (eg, matrix metalloproteinase 7, platelet-derived growth factor subunit B) (eFigure 2 in the Supplement). There were low to moderate correlations between levels of protein biomarkers (Pearson correlation coefficient *r*: median, 0.24 [interquartile range, 0.10-0.41]; eFigure 3 in the Supplement).

### Observational Associations of Adiposity With Proteomics

**Figure 2.**
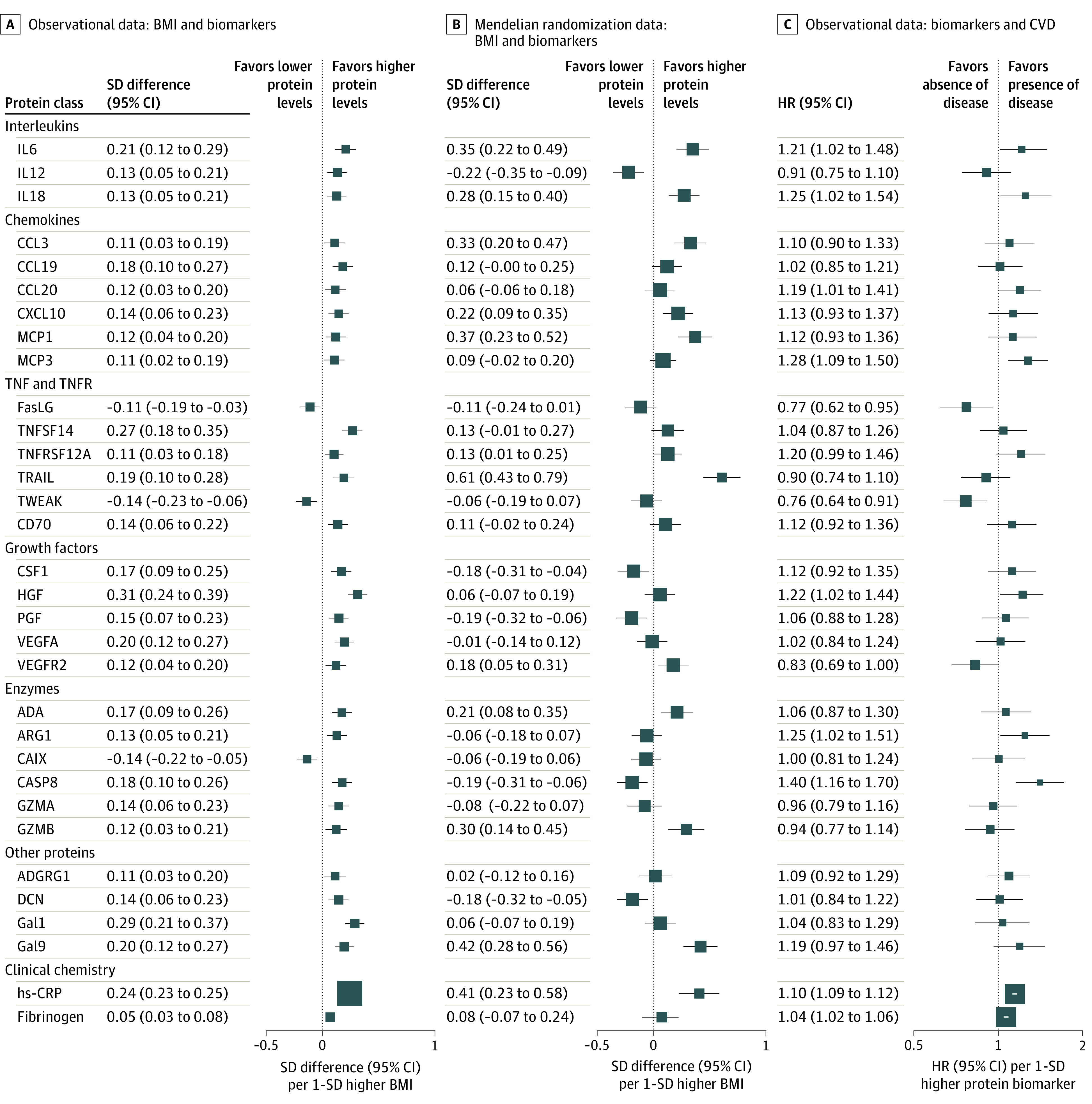
Associations of Observational and Genetically Instrumented Body Mass Index (BMI) With Proteins and Proteins With Vascular Events A, Adjusted SD differences (95% CI) of protein biomarkers per 1-SD higher observational BMI (calculated as weight in kilograms divided by height in meters squared) for 30 protein biomarkers with false-discovery rate–corrected *P* < .05. B, Corresponding estimates per 1-SD higher genetically elevated BMI. The observational estimates were adjusted for age, age squared, sex, region, smoking, alcohol use, education, household income, self-rated health, systolic blood pressure, diabetes, statin treatment, prior kidney disease, and fasting time. The mendelian randomization estimates were adjusted for age, age squared, sex, region, the first 12 principal components, education, smoking status, and alcohol use. The SD for BMI in the whole China Kadoorie Biobank cohort was 3.4. C, Adjusted hazard ratios (HRs) with 95% CIs of vascular events (*International Statistical Classification of Diseases and Related Health Problems, Tenth Revision* codes I00-I09, I16-I25, I27-I88, I95-I99, and I10-I15 [only if fatal]) per 1-SD higher protein biomarkers. Models were adjusted for age, age squared, sex, region, smoking status, alcohol use, education, household income, self-rated health, systolic blood pressure, diabetes, statin treatment, prior kidney disease, and fasting time. Within each column, the size of the box was inversely proportional to the variance of the SD difference or logHR. In columns (B) and (C), the size of the box was scaled up because of the larger SDs compared with those in (A). ADA indicates adenosine deaminase; ADGRG1, adhesion G protein-coupled receptor G1; ARG1, arginase 1; CAIX, carbonic anhydrase 9; CASP8, caspase 8; CCL3, chemokine (C-C motif) ligand 3; CCL19, chemokine (C–C motif) ligand 19;CCL20, chemokine (C–C motif) ligand 20; CSF1, colony-stimulating factor 1; CVD, cardiovascular disease; CXCL10, C-X-C motif chemokine ligand 10; DCN, decorin; FasLG, Fas ligand; Gal1, galactokinase; Gal9, galectin-9; GZMA, granzyme A; GZMB, granzyme B; IL6, interleukin-6; IL12, interleukin-12; IL18, interleukin-18; HGF, hepatocyte growth factor; hs-CRP, high-sensitivity C-reactive protein; MCP1, monocyte chemoattractant protein–1; MCP3, monocyte chemotactic protein–3; PGF, placenta growth factor; TNFSF14, tumor necrosis factor superfamily member 14; TNFRSF12A, TNF receptor superfamily member 12A; TRAIL, TNF-related apoptosis-inducing ligand; TWEAK, TNF-related weak inducer of apoptosis; VEGFA, vascular endothelial growth factor A; VEGFR2, vascular endothelial growth factor receptor 2.

After adjusting for multiple comparisons, significant associations of BMI with 30 of the 90 protein biomarkers were identified (at 5% FDR; Figure 2A; eTable 7 in the Supplement). Similarly, based on the Rényi plot, 31 protein biomarkers surpassed the threshold, providing evidence of associations of BMI with these proteins on observational analysis (eFigure 4 in the Supplement), including the 30 proteins surpassing an FDR less than 5%. When examining the shape of associations (eFigure 5 in the Supplement), association with BMI was approximately linear for interleukins (IL6, IL12, and IL18), chemokines (chemokine [C-C motif] ligand 3, monocyte chemoattractant protein–1, and monocyte chemotactic protein–3), tumor necrosis factor (TNF) and TNF-receptor (Fas ligand, TNF-related apoptosis-inducing ligand [TRAIL], and TNF-related weak inducer of apoptosis [TWEAK]), growth factors (colony-stimulating factor 1 [CSF1], hepatocyte growth factor [HGF], and vascular endothelial growth factor A), enzymes (caspase 8), and other secreted proteins (decorin [DCN], galactokinase, and galectin-9). The associations of proteomics with other adiposity traits (eg, waist circumference [*r* = 0.96]) were similar to those with BMI (eFigure 6 in the Supplement).

### Genetic Associations of BMI With Proteomics

In genetic analyses, the 30 BMI-associated protein biomarkers (at 5% FDR) in observational analyses also showed similar associations to genetically elevated BMI (Pearson correlation coefficient *r* = 0.40; Figure 2B; eFigure 7 in the Supplement). There was evidence of genetically estimated associations of BMI (per 1-SD higher BMI) with 6 proteins at 5% FDR (SD differences: IL6, 0.35 [95% CI, 0.22-0.49] SD; IL18, 0.28 [95% CI, 0.15-0.40] SD; CCL3, 0.33 [95% CI, 0.20-0.47] SD; MCP1, 0.37 [95% CI, 0.23-0.52] SD; TRAIL, 0.61 [95% CI, 0.43-0.79] SD; and galectin-9, 0.42 [95% CI, 0.28 -0.56] SD) and suggestive evidence for 9 proteins (uncorrected *P* < .05: IL12, –0.22 [95% CI, –0.35 to –0.09]; C-X-C motif chemokine ligand 10, 0.22 [95% CI, 0.09-0.35]; TNF receptor superfamily member 12A, 0.13 [95% CI, 0.01-0.25]; CSF1, –0.18 [95% CI, –0.31 to –0.04]; vascular endothelial growth factor receptor 2 [VEGFR2], 0.18 [95% CI, 0.05-0.31]; adenosine deaminase, 0.21 [95% CI, 0.08-0.35]; caspase 8, –0.19 [95% CI, –0.31 to –0.06]; granzyme B, 0.30 [95% CI, 0.14-0.45]; and DCN, –0.18 [95% CI, –0.32 to –0.05]) (Figure 2B). The Cochran *Q* test found no evidence for differences between the genetic and observational estimates, with the exception of 3 proteins (IL12, CSF1, and DCN), for which the genetic estimate was directionally opposite to the observational estimate (genetic estimates: IL12, −0.22 [95% CI, −0.35 to −0.09]; CSF1, −0.18 [95% CI, −0.31 to −0.04]; DCN, −0.18 [95% CI, −0.32 to −0.05]; observational estimates: IL12, 0.13 [95% CI, 0.05-0.21]; CSF1, 0.17 [95% CI, 0.09-0.25]; DCN, 0.14 [95% CI, 0.06-0.23]).

**Figure 3.**
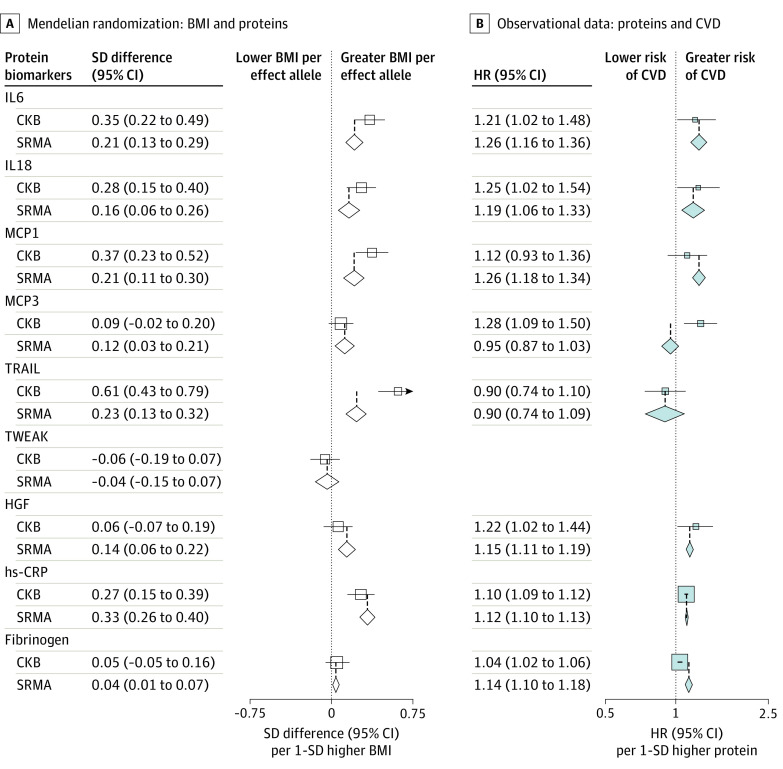
Meta-analysis of Genetic Associations of Body Mass Index (BMI) With Proteins and Observational Associations of Proteins With Cardiovascular Disease (CVD) A, Genetic associations of BMI with selected proteins (estimates from individual studies are shown in eFigure 8 in the Supplement). B, Observational associations of selected proteins with cardiovascular disease (estimates from individual studies are shown in Figure 4). Diamonds denote pooled estimates from meta-analyses, and open boxes denote estimates in the China Kadoorie Biobank (CKB). For C-reactive protein (CRP) and fibrinogen, outcomes estimates were extracted from previous studies of the largest known sample sizes.^33,34,35,36^ No prospective studies were identified on TNF-related weak inducer of apoptosis (TWEAK) and CVD. IL6 indicates interleukin-6; IL8, interleukin-8; IL12, interleukin-12; HGF, hepatocyte growth factor; hs-CRP, high-sensitivity CRP; MCP1, monocyte chemoattractant protein–1; MCP3, monocyte chemotactic protein–3; SRMA, systematic review and meta-analysis; TRAIL, TNF-related apoptosis-inducing ligand.

When meta-analyzing genetic estimates in the present study with estimates from published studies^15,21,22,31,32^ where data permitted, there was evidence for positive associations of genetically elevated BMI with IL6, IL18, MCP1, MCP3, TRAIL, and HGF (Figure 3A; eFigure 8 in the Supplement).^33,34,35,36^ Taken together, each 1-SD higher genetically estimated BMI was associated with an IL6 level 0.21 (95% CI, 0.13-0.29) SD higher, an IL18 level 0.16 (95% CI, 0.06-0.26) SD higher, an MCP1 level 0.21 (95% CI, 0.11-0.30) SD higher, an MCP3 level 0.12 (95% CI, 0.03-0.21) SD higher, a TRAIL level 0.23 (95% CI, 0.13-0.32) SD higher, and an HGF level 0.14 (95% CI, 0.06-0.22) SD higher. In addition, mendelian randomization identified genetically estimated associations of BMI with high-sensitivity CRP and fibrinogen levels, with pooled SD differences of 0.33 (95% CI, 0.26-0.40) SD and 0.04 (95% CI, 0.01-0.07) SD, respectively.

### Associations of Proteomics With Vascular Diseases

Of the 30 BMI-associated protein biomarkers, 10 proteins (HRs: IL6, 1.21 [95% CI, 1.02-1.48]; IL18, 1.25 [95% CI, 1.02-1.54]; chemokine [C–C motif] ligand 20, 1.19 [95% CI, 1.01-1.41]; MCP3, 1.28 [95% CI, 1.09-1.50]; Fas ligand, 0.77 [95% CI, 0.62-0.95]; TWEAK, 0.76 [95% CI, 0.64-0.91]; HGF, 1.22 [95% CI, 1.02-1.44]; VEGFR2, 0.83 [95% CI, 0.69-1.00]; Arginase 1, 1.25 [95% CI, 1.02-1.51]; and caspase 8, 1.40 [95% CI, 1.16-1.70]) were nominally associated with incident risk of vascular disease events in CKB (uncorrected *P* < .05, Figure 2C). Likewise, based on the Rényi plot, we identified 23 significant protein biomarkers (eFigure 4 in the Supplement), including these 10 proteins. Of these 10 proteins, there was consistency in the direction of results between the associations of BMI with protein and protein with vascular events. In other words, when BMI was associated with altered levels of a protein, corresponding altered levels of that same protein were associated with a higher risk of CVD. When adjusting for age at baseline, age squared, sex, region, education, household income, alcohol use, self-rated health, statin treatment, prior kidney disease, and fasting time, further simultaneous adjustment for all 10 proteins attenuated the positive association between BMI and risk of vascular events by 38%. Likewise, adding systolic blood pressure and diabetes to the model attenuated the positive association between BMI and risk of vascular events by 38% and 6%, respectively. When the 10 proteins, systolic blood pressure, and diabetes were included in the same model, the positive association of BMI with risk of vascular events was attenuated by 66%.

**Figure 4.**
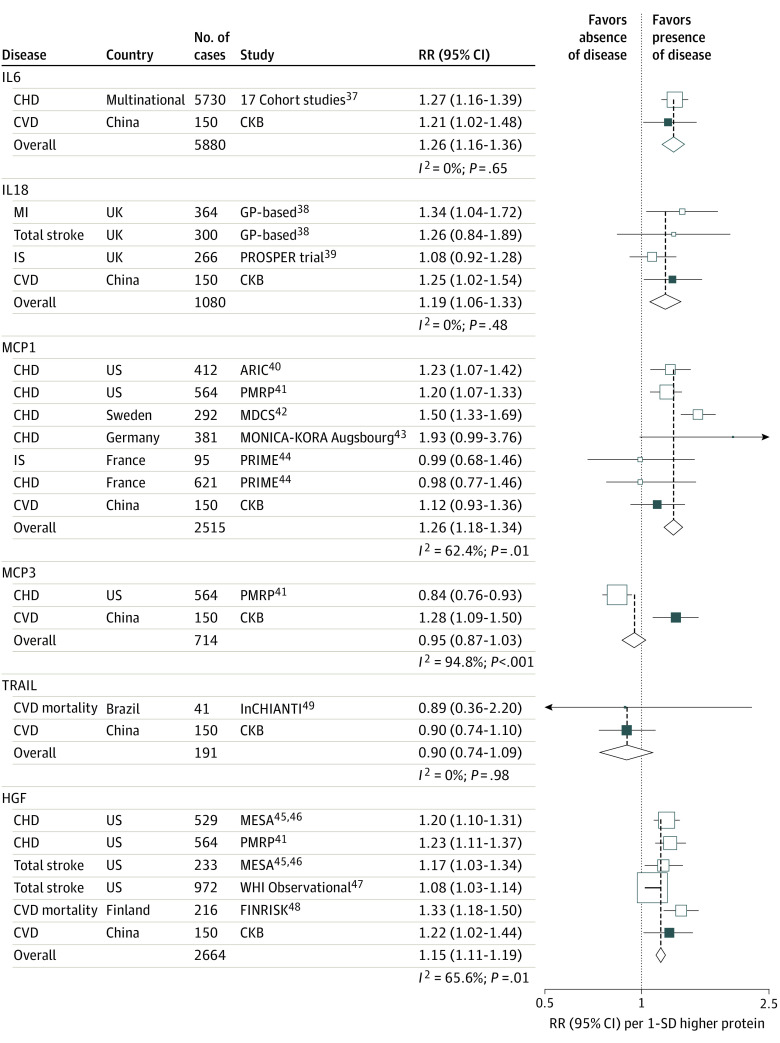
Meta-analysis of Observational Associations of Proteins With Cardiovascular Disease (CVD) Boxes represent the relative risks (RRs) of CVD per 1-SD higher protein for individual studies, with the size of the box inversely proportional to the variance of the logRR. Open boxes represent previously published studies,^37,38,39,40,41,42,43,44,45,46,47,48^ and the black box represents the China Kadoorie Biobank (CKB). Diamonds represent summary RRs for each protein. Blood pressure, diabetes, and/or lipids were adjusted for in all studies. ARIC indicates Atherosclerosis Risk in Communities; CHD, coronary heart disease; FINRISK, Finnish risk study; GP, general practitioner; HGF, hepatocyte growth factor; IL6, interleukin-6; IL18, interleukin-18; InCHIANTI, Invecchiare in Chianti; IS, ischemic stroke; MCP1, monocyte chemoattractant protein–1; MCP3, monocyte chemotactic protein–3; MDCS, the Malmö Diet and Cancer Study; MESA, Multi-Ethnic Study of Atherosclerosis; MI, myocardial infarction; MONICA-KORA, Myocardial Infarction Augsbourg–Cooperative Health Research in the Region Augsburg; PMRP, Personalized Medicine Research Project; PRIME, the Panitumumab Randomized Trial in Combination With Chemotherapy for Metastatic Colorectal Cancer to Determine Efficacy; PROSPER: the Prospective Study of Pravastatin in the Elderly; RR, relative risk; WHI, Women's Health Initiative.

When pooling the observational estimates in CKB with previous prospective studies involving 6 of these proteins (no prospective studies were identified for TWEAK), there were positive associations of IL6 (relative risk [RR], 1.26 [95% CI, 1.16-1.36]), IL18 (RR, 1.19 [1.06-1.33]), MCP1 (RR, 1.26 [1.18-1.34]), and HGF (RR, 1.15 [1.11-1.19]) with CVD (Figure 4).^37,38,39,40,41,42,43,44,45,46,47,48,49^ Of these proteins, there was consistency in the direction of outcomes between the genetic associations of BMI with proteins and observational associations of proteins with CVD for IL6 (SD difference, 0.21 [95% CI, 0.13-0.29] SD vs HR, 1.26 [95% CI, 1.16-1.36] SD), IL18 (SD difference, 0.16 [95% CI, 0.06-0.26] SD vs HR, 1.19 [95% CI, 1.06-1.33] SD), MCP1 (SD difference, 0.21 [95% CI, 0.11-0.30] SD vs HR, 1.26 [95% CI, 1.18-1.34] SD), and HGF (SD difference, 0.14 [95% CI, 0.06-0.22] SD vs HR, 1.15 [95% CI, 1.11-1.19] SD; Figure 3). For 4 proteins with available *cis* instruments (IL18, TRAIL, TWEAK, and HGF), 2-sample mendelian randomization analysis suggested inverse associations of genetically elevated TWEAK with both CHD and ischemic stroke (HR, 0.93 [95% CI, 0.89-0.97] and 0.97 [95% CI, 0.93-0.99]; eFigure 9 and eTables 12 and 13 in the Supplement). When *trans*-acting SNVs were included in the genetic instruments for MCP1 and HGF, there were positive genetic associations with ischemic stroke, but the genetic association for MCP1 was entirely driven by 38 *trans*–protein quantitative trait loci (pQTLs) and, for HGF, by the 2 *trans*-pQTLs (eTable 8 and eFigure 10 in the Supplement). There was a positive genetic association between TRAIL and CHD (OR, 1.03 [95% CI, 1.01-1.06]), also driven by *trans*-pQTL (eTable 8 in the Supplement). Using published pQTL and stroke association data, colocalization analysis showed no evidence for a role of the TWEAK locus (posterior probability of a model with 1 shared common variant [PP4]: 0.006) or HGF locus (PP4: 0.005) in ischemic stroke (eFigure 11 and eMethods in the Supplement).

### Subgroup and Sensitivity Analyses

The genetic associations of BMI with proteomics were similar when using an unweighted score, albeit with less precision (eFigure 12 in the Supplement). For the weighted score, the patterns of genetic associations were similar when external weights from Biobank Japan or the meta-analysis of UK Biobank and the Genetic Investigation of Anthropometric Traits were used (eFigure 13 in the Supplement). The observational associations of BMI with proteomics were broadly consistent by sex, region, smoking status, physical activity, random plasma glucose level, and hypertension status (eFigure 14 in the Supplement). The observational associations of protein biomarkers with major adverse coronary events were generally consistent with those seen for vascular events (eFigure 15 in the Supplement). Mendelian randomization (MR)–Egger and weighted median estimates were consistent with the individual participant–level data estimates, but MR-Egger estimates were more imprecise (Pearson correlation coefficient *r* = 0.89; eFigure 16 and eTable 8 in the Supplement). The genetic associations of BMI with protein biomarkers were similar when excluding from the BMI genetic score SNVs within 1 Mbp of the genes encoding protein biomarkers (eFigure 17 in the Supplement). Mendelian randomization–Steiger results provided support that the orientation of the genetic associations was from adiposity traits to proteins (eTable 9 in the Supplement).

## Discussion

In this Chinese population, which was relatively lean, BMI was associated with a range of protein biomarkers, which covered chemokines, interleukins, TNF and the TNF-receptor superfamily, growth factors, enzymes, cell surface proteins, and extracellular proteins. The pattern of associations with proteomics was similar for other general and central adiposity traits (eg, waist circumference). Mendelian randomization analyses suggested directionally consistent associations of proteins with genetically elevated BMI and observational BMI, with the evidence more robust for IL6, IL18, MCP1, MCP3, TRAIL, and HGF (eFigure 18 in the Supplement). Some of the BMI-associated protein biomarkers (eg, IL6, IL18, MCP1, and HGF) were observationally associated with risk of CVD, providing potential insights into cardiometabolic disease pathways.

We showed that increased or decreased levels of several proteins that BMI is genetically estimated to associate with were also observationally associated with risk of CVD. Our study findings are consistent with previous prospective studies showing positive associations of IL6, IL18, MCP1, and HGF with CVD risk. Interleukin-6 plays a key role in inflammatory responses through binding to its receptor IL6R.^50^ Mendelian randomization studies have suggested that blockage of IL6R may lower risk of CHD, but the association between IL6 and CHD remains less clear.^51^ In addition, MCP1 is an inflammatory chemokine that plays a key role in atherogenesis and atheroprogression.^52^ On binding to its receptors, CCR2 and CCR4, MCP1 recruits monocytes and basophils to sites of inflammation, including the subendothelial space of the atherogenic arterial wall.^53^ A recent mendelian randomization study showed an association of MCP1 with ischemic stroke.^54^ However, in that study, none of the MCP1 instruments were located in or near the *MCP1* gene. While we were able to replicate the association of MCP1 with risk of CHD and ischemic stroke,^54^ as with the prior study, our mendelian randomization estimate relies entirely on *trans*-acting instruments. Therefore, nonspecific (ie, horizontally pleiotropic) effects of the MCP1 *trans*-acting instruments cannot be excluded.^20^

There is emerging evidence that inhibition of inflammatory pathways may represent an effective therapeutic strategy for the treatment and prevention of CVD, with several randomized trials currently underway to test various new drugs (eTable 10 in the Supplement).^4,55^ In the Canakinumab Antiinflammatory Thrombosis Outcome Study (CANTOS) trial,^56^ IL1β inhibition with canakinumab lowered plasma CRP and IL6 and recurrent cardiovascular events. Recently, the Colchicine Cardiovascular Outcomes Trial (COLCOT) trial^57^ reported that colchicine lowered risk of ischemic CVD, compared with placebo, among patients with a recent myocardial infarction. A phase IIa trial^58^ showed that anti-IL18 monoclonal antibody lowered hemoglobin A_1c_ levels, suggesting that IL18 might be a potential therapeutic target for type 2 diabetes. In line with this randomized clinical trial, we conducted 2-sample mendelian randomization using DIAGRAM and show genetic evidence of an association of IL18 inhibition on type 2 diabetes (odds ratio, 0.89 [95% CI, 0.81-0.99]; eMethods in the Supplement). Furthermore, recent studies have reported TWEAK, matrix metallopeptidase 12, CD40, and scavenger receptor class A member 5 as promising targets for the treatment of ischemic stroke.^54,59^

The strengths of the CKB included use of a prospective design, coverage of a broad range of blood-based protein biomarkers involved in multiple biological pathways, assessment of different adiposity measures, and use of 3 different, complementary types of analyses to assess genetically estimated associations of adiposity, proteins, and CVD risk in the same study population.

### Limitations

Our study also had several limitations. First, the genetic analyses of BMI and proteomics had limited power. Therefore, we conducted meta-analyses where possible, combining genetic estimates for 8 of the 30-BMI associated proteins from CKB with those from published studies, which showed concordant associations. Second, it is plausible that a subset of SNVs included in the BMI genetic score may affect protein biomarkers independently of BMI, potentially violating the assumptions of mendelian randomization.^60^ However, we showed that weighted median and MR-Egger estimates were broadly consistent with the inverse‐variance weighted estimates in CKB (eFigure 16 in the Supplement). Moreover, our finding for IL6 is generally concordant with previous mendelian randomization studies using different genetic variants to construct the BMI genetic score.^22,24^ Third, it is possible that SNPs used in the instrument for adiposity might more strongly influence proteins than adiposity; we explored this through mendelian randomization Steiger^61^ and by removing SNVs from the BMI score near genes encoding proteins. Lastly, there is lack of validation of the observational associations of adiposity with proteomics in a non-European population. Of the 30 protein biomarkers associated with BMI in CKB, we compared the observational associations for 14 protein biomarkers also examined in 3362 European adults and showed consistent associations for 11 proteins (eTable 11 in the Supplement).^15^ However, that European study used a different platform (ie, the SomaScan assay) to measure proteomics and adjusted only for age and sex, so, notwithstanding the potential for *trans*-ethnic heterogeneity, the results are not directly comparable.

## Conclusions

In summary, our study in a Chinese population with a relatively low mean BMI showed that adiposity was associated with a range of inflammatory and immune-associated protein biomarkers. For many such biomarkers, there was consistency between observational and genetic findings in their associations with BMI. Some of the BMI-associated protein biomarkers were also shown to be observationally associated with risk of incident CVD. These findings provide potential insights into the biological mechanisms linking adiposity and cardiovascular and metabolic diseases.

## Notes

Supplement. **eFigure 1.** Genetic risk score (GRS) for BMI in CKB. **eFigure 2.** Histograms of protein biomarkers. **eFigure 3.** Correlation matrix of protein biomarkers. **eFigure 4.** Observational associations of BMI with protein biomarkers and of protein biomarkers with CVD. **eFigure 5.** Associations of BMI with 21 selected protein biomarkers. **eFigure 6.** Global comparison of 1-SD differences of 90 protein biomarkers associated with 1-SD higher BMI versus other adiposity traits. **eFigure 7.** Global comparison of 1-SD differences of 90 protein biomarkers associated with 1-SD higher observational BMI versus genetically elevated BMI. **eFigure 8.** Meta-analysis of causal associations of BMI with selected protein biomarkers. **eFigure 9.** Meta-analysis of genetic associations of BMI with proteins and of proteins with CVD. **eFigure 10.** Associations of cis and trans SNP in relation to protein biomarkers and CVD. **eFigure 11.** Colocalisation analysis of TWEAK, HGF, and ischaemic stroke. **eFigure 12.** Genetic associations of BMI with proteomics comparing weighted and unweighted GRS. **eFigure 13.** Genetic associations of BMI with proteomics using different external weights. **eFigure 14.** Associations of BMI with proteomics by population subgroup. **eFigure 15.** Observational associations of protein biomarkers with CVD. **eFigure 16.** Causal associations of BMI and proteomics using different MR methods. **eFigure 17.** Sensitivity analyses of genetic associations of BMI and proteomics in CKB. **eFigure 18.** Central illustration of BMI, protein biomarkers, and risk of CVD. **eFigure 19. **Internal and external controls. **eFigure 20. **Assay specificity. **eTable 1.** List of 92 proteomics quantified by the OLINK immuno-oncology assay. **eTable 2.** Grouping of the 92 proteins measured by OLINK Immuno-Oncology assay. **eTable 3.** Limit of detection and coefficient of variation of 92 protein biomarkers. **eTable 4.** Genetic variants associated with BMI in the GIANT consortium. **eTable 5.** Associations of potential confounders with BMI GRS. **eTable 6.** Baseline characteristics of study participants by BMI in all CKB participants. **eTable 7.** Associations of BMI with proteomics and of proteomics with CVD. **eTable 8.** Genetic associations of MCP1, HGF, IL18, and TRAIL with risk of CVD. **eTable 9.** P-values from the MR Steiger test. **eTable 10.** Randomised controlled trials of anti-inflammatory therapy in CVD **eTable 11.** Observational associations between BMI and proteins in CKB and the INTERVAL study. **eMethods.** **eReferences.** **eTable 12. **SNPs that were used as instruments for selected protein biomarkers. **eTable 13. **Selected proteins, data sources, and calculation in meta-analysis and two-sample Mendelian randomisation.
