# Supplementary material for: Associations of Adiposity, Circulating Protein Biomarkers, and Risk of Major Vascular Diseases
Source: JAMA Cardiol. 2020 Dec 2;6(3):1–11. doi: 10.1001/jamacardio.2020.6041 (PMC7711564; doi:10.1001/jamacardio.2020.6041)
Supplement: Supplement. — eFigure 1. Genetic risk score (GRS) for BMI in CKB. eFigure 2. Histograms of protein biomarkers. eFigure 3. Correlation matrix of protein biomarkers. eFigure 4. Observational associations of BMI with protein biomarkers and of protein biomarkers with CVD. eFigure 5. Associations of BMI with 21 selected protein biomarkers. eFigure 6. Global comparison of 1-SD differences of 90 protein biomarkers associated with 1-SD higher BMI versus other adiposity traits. eFigure 7. Global comparison of 1-SD differences of 90 protein biomarkers associated with 1-SD higher observational BMI versus genetically elevated BMI. eFigure 8. Meta-analysis of causal associations of BMI with selected protein biomarkers. eFigure 9. Meta-analysis of genetic associations of BMI with proteins and of proteins with CVD. eFigure 10. Associations of cis and trans SNP in relation to protein biomarkers and CVD. eFigure 11. Colocalisation analysis of TWEAK, HGF, and ischaemic stroke. eFigure 12. Genetic associations of BMI with proteomics comparing weighted and unweighted GRS. eFigure 13. Genetic associations of BMI with proteomics using different external weights. eFigure 14. Associations of BMI with proteomics by population subgroup. eFigure 15. Observational associations of protein biomarkers with CVD. eFigure 16. Causal associations of BMI and proteomics using different MR methods. eFigure 17. Sensitivity analyses of genetic associations of BMI and proteomics in CKB. eFigure 18. Central illustration of BMI, protein biomarkers, and risk of CVD. eFigure 19. Internal and external controls. eFigure 20. Assay specificity. eTable 1. List of 92 proteomics quantified by the OLINK immuno-oncology assay. eTable 2. Grouping of the 92 proteins measured by OLINK Immuno-Oncology assay. eTable 3. Limit of detection and coefficient of variation of 92 protein biomarkers. eTable 4. Genetic variants associated with BMI in the GIANT consortium. eTable 5. Associations of potential confounders with BMI GRS. eTable 6. Base [file jamacardiol-e206041-s001.pdf]

Supplemental Online Content

Pang Y, Kartsonaki C, Lv J, et al. Associations of adiposity, circulating protein biomarkers, and risk of major vascular diseases. *JAMA Cardiol*. Published online December 2, 2020. doi:10.1001/jamacardio.2020.6041

eFigure 1. Genetic risk score (GRS) for BMI in CKB.
eFigure 2. Histograms of protein biomarkers.
eFigure 3. Correlation matrix of protein biomarkers.
eFigure 4. Observational associations of BMI with protein biomarkers and of protein biomarkers with CVD.
eFigure 5. Associations of BMI with 21 selected protein biomarkers.
eFigure 6. Global comparison of 1-SD differences of 90 protein biomarkers associated with 1-SD higher BMI versus other adiposity traits.
eFigure 7. Global comparison of 1-SD differences of 90 protein biomarkers associated with 1-SD higher observational BMI versus genetically elevated BMI.
eFigure 8. Meta-analysis of causal associations of BMI with selected protein biomarkers.
eFigure 9. Meta-analysis of genetic associations of BMI with proteins and of proteins with CVD.
eFigure 10. Associations of cis and trans SNP in relation to protein biomarkers and CVD.
eFigure 11. Colocalisation analysis of TWEAK, HGF, and ischaemic stroke.
eFigure 12. Genetic associations of BMI with proteomics comparing weighted and unweighted GRS.
eFigure 13. Genetic associations of BMI with proteomics using different external weights.
eFigure 14. Associations of BMI with proteomics by population subgroup.
eFigure 15. Observational associations of protein biomarkers with CVD.
eFigure 16. Causal associations of BMI and proteomics using different MR methods.
eFigure 17. Sensitivity analyses of genetic associations of BMI and proteomics in CKB.
eFigure 18. Central illustration of BMI, protein biomarkers, and risk of CVD.
eFigure 19. Internal and external controls.
eFigure 20. Assay specificity.
eTable 1. List of 92 proteomics quantified by the OLINK immuno-oncology assay.
eTable 2. Grouping of the 92 proteins measured by OLINK Immuno-Oncology assay.
eTable 3. Limit of detection and coefficient of variation of 92 protein biomarkers.
eTable 4. Genetic variants associated with BMI in the GIANT consortium.
eTable 5. Associations of potential confounders with BMI GRS.
eTable 6. Baseline characteristics of study participants by BMI in all CKB participants.
eTable 7. Associations of BMI with proteomics and of proteomics with CVD.
eTable 8. Genetic associations of MCP1, HGF, IL18, and TRAIL with risk of CVD.
eTable 9. P-values from the MR Steiger test.
eTable 10. Randomised controlled trials of anti-inflammatory therapy in CVD
eTable 11. Observational associations between BMI and proteins in CKB and the INTERVAL study.
eMethods.
eReferences.
eTable 12. SNPs that were used as instruments for selected protein biomarkers
eTable 13. Selected proteins, data sources, and calculation in meta-analysis and two-sample Mendelian randomisation

This supplemental material has been provided by the authors to give readers additional information about their work.

© 2020 Pang et al. *JAMA Cardiology.*

© 2020 Pang et al. *JAMA Cardiology.*

eFigure 1. Genetic risk score (GRS) for BMI in CKB

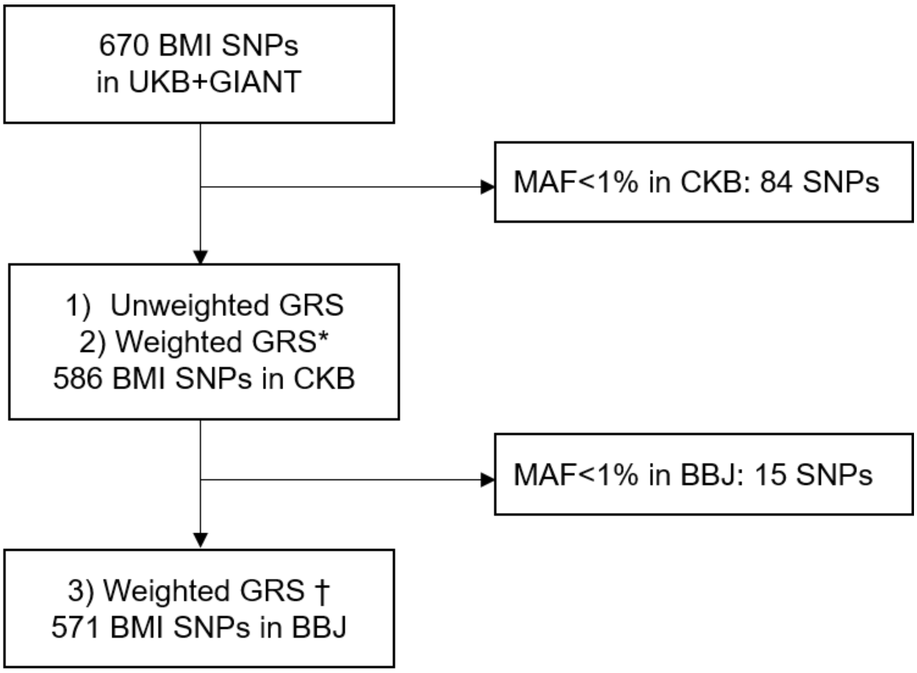

	1) Unweighted GRS	2) Weighted GRS*	3) Weighted GRS†
**No. SNPs**	586	586	571
**R^2**	1.10%	2.04%	2.06%
**F-statistic**	1071	1577	1593

R^2 and F-statistic were calculated in 75,736 participants in the population subset.

* External weights were taken from UKB+GIANT.
† External weights were taken from BBJ.
Proxy SNPs were identified for 5 SNPs unavailable in BBJ
(in high linkage disequilibrium [$R^2 \geq 0.8$] in CEU).

© 2020 Pang et al. *JAMA Cardiology.*

eFigure 2. Histograms of protein biomarkers

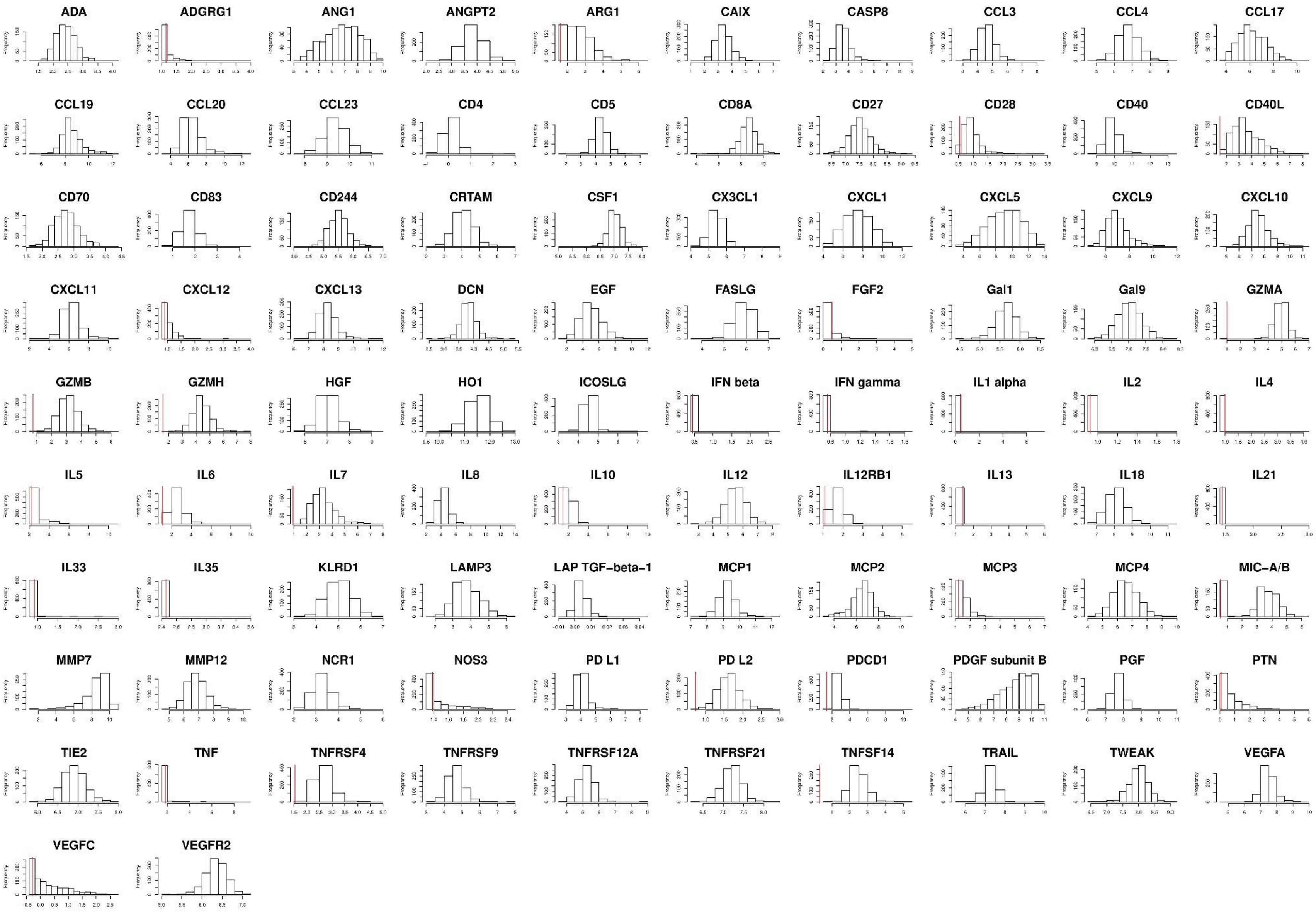

The red line denotes the LOD for individual proteins.

© 2020 Pang et al. *JAMA Cardiology.*

eFigure 3. Correlation matrix of protein biomarkers

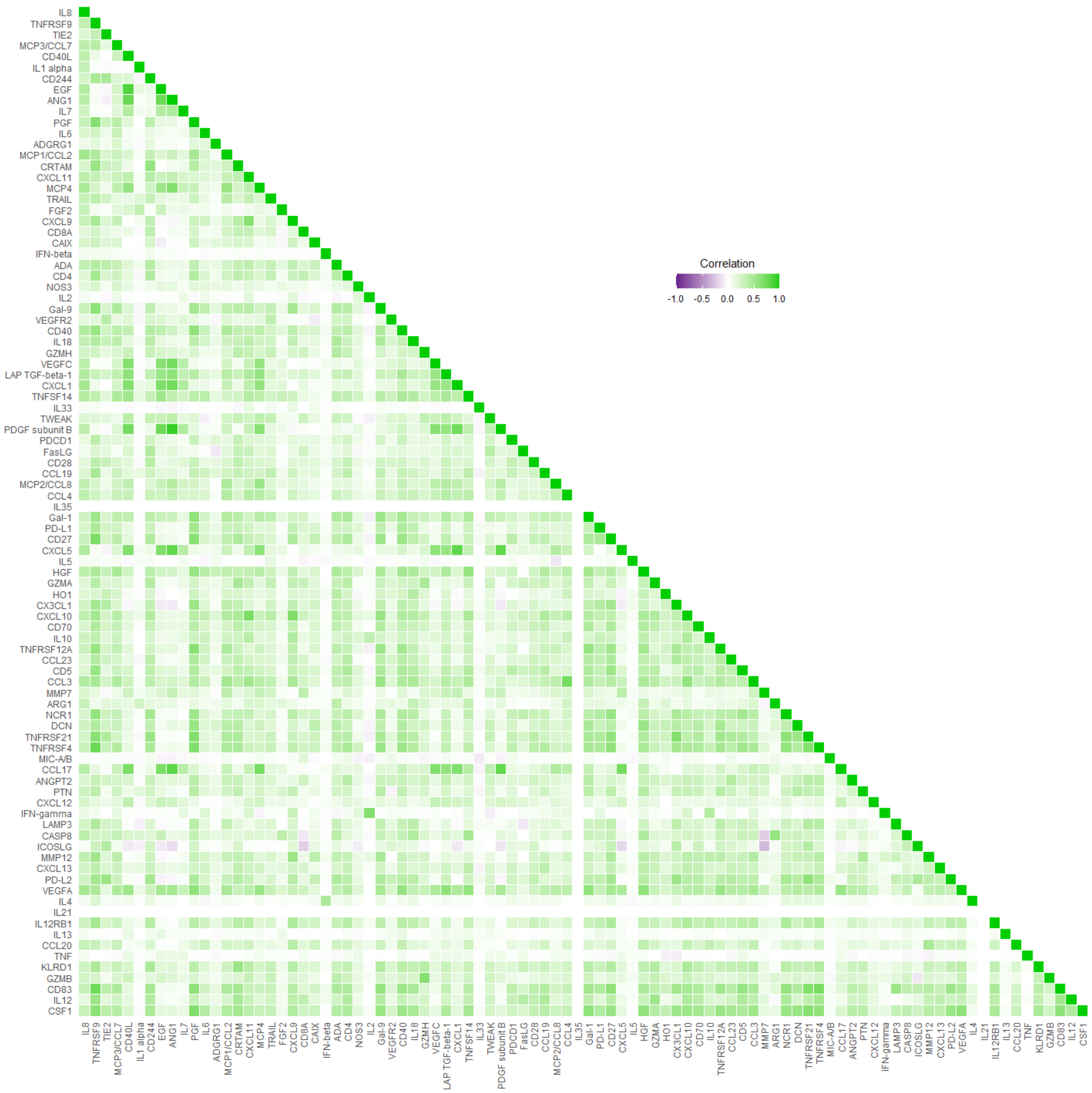

© 2020 Pang et al. *JAMA Cardiology.*

eFigure 4. Observational associations of BMI with protein biomarkers and of protein biomarkers with CVD

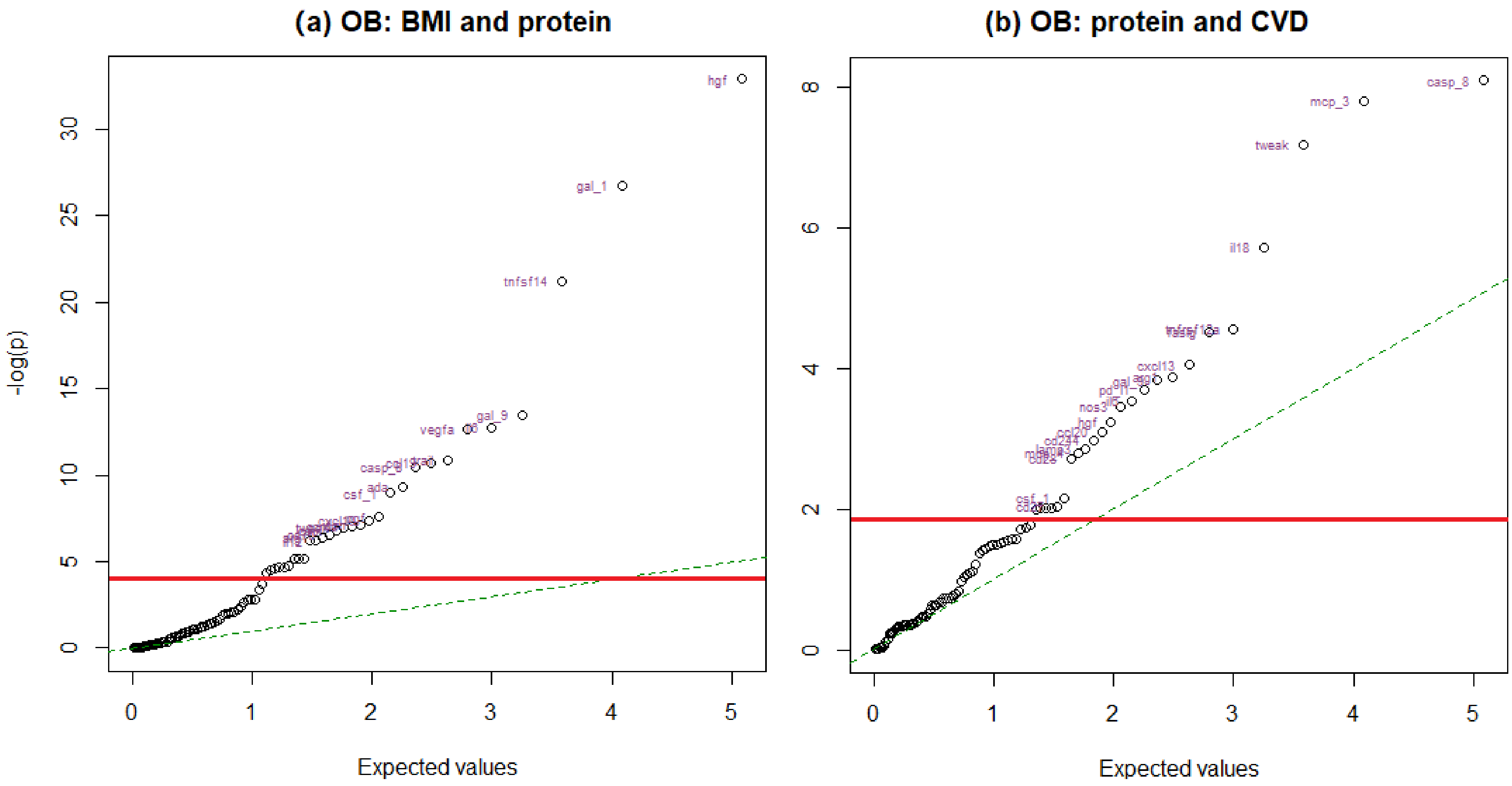

The isolation of protein biomarkers in the Rényi plot above the expected unit line suggests significant associations taking into account multiple testing. In both plots, the red line denotes the cut-off but its choice is somewhat subjective. Number of proteins above the cut-off: n=31 in the left panel and n=23 in the right panel.

© 2020 Pang et al. *JAMA Cardiology.*

eFigure 5. Associations of BMI with 21 selected protein biomarkers

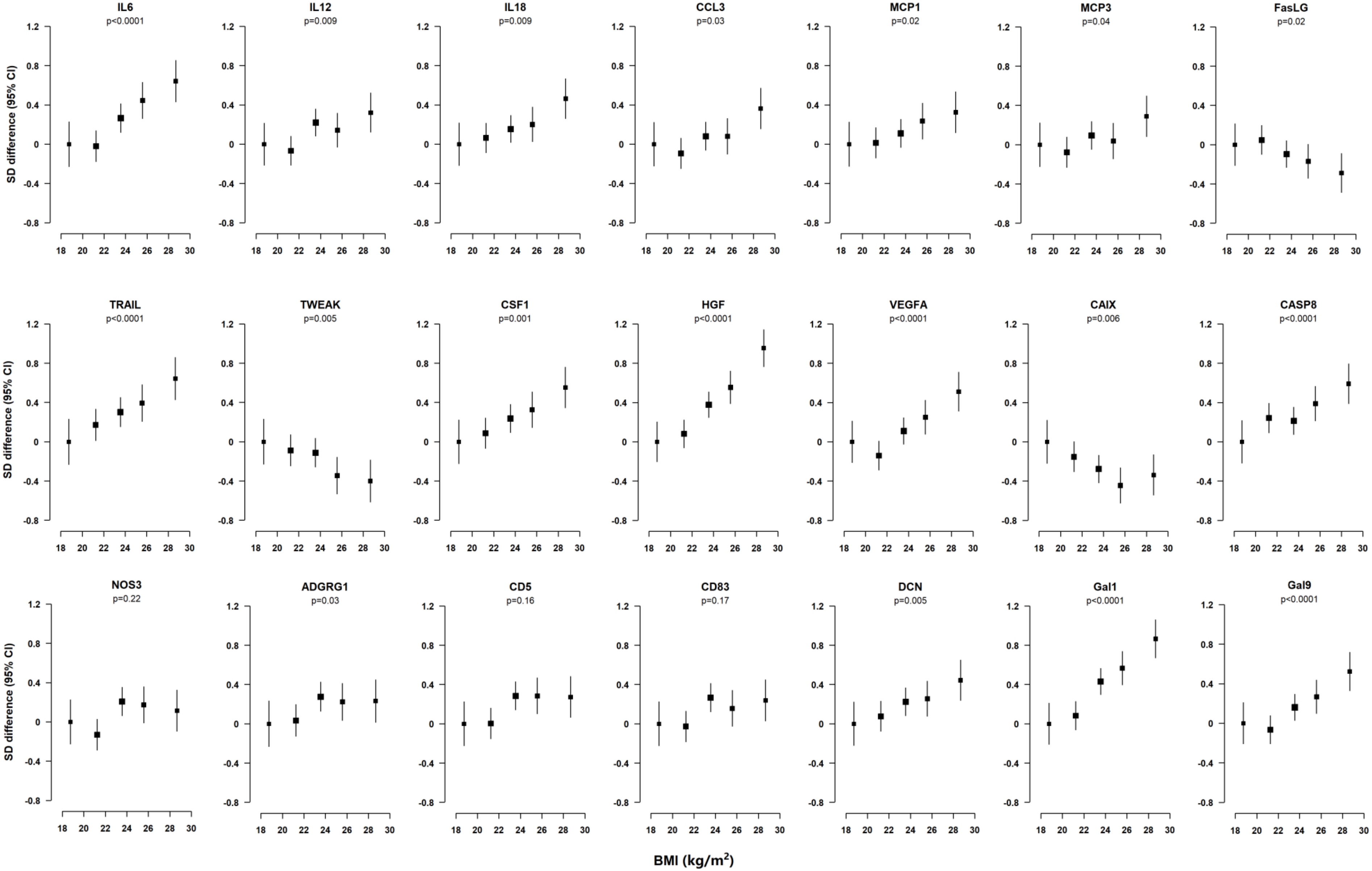

© 2020 Pang et al. *JAMA Cardiology.*

eFigure 6. Global comparison of 1-SD differences of 90 protein biomarkers associated with 1-SD higher BMI *versus* other adiposity traits

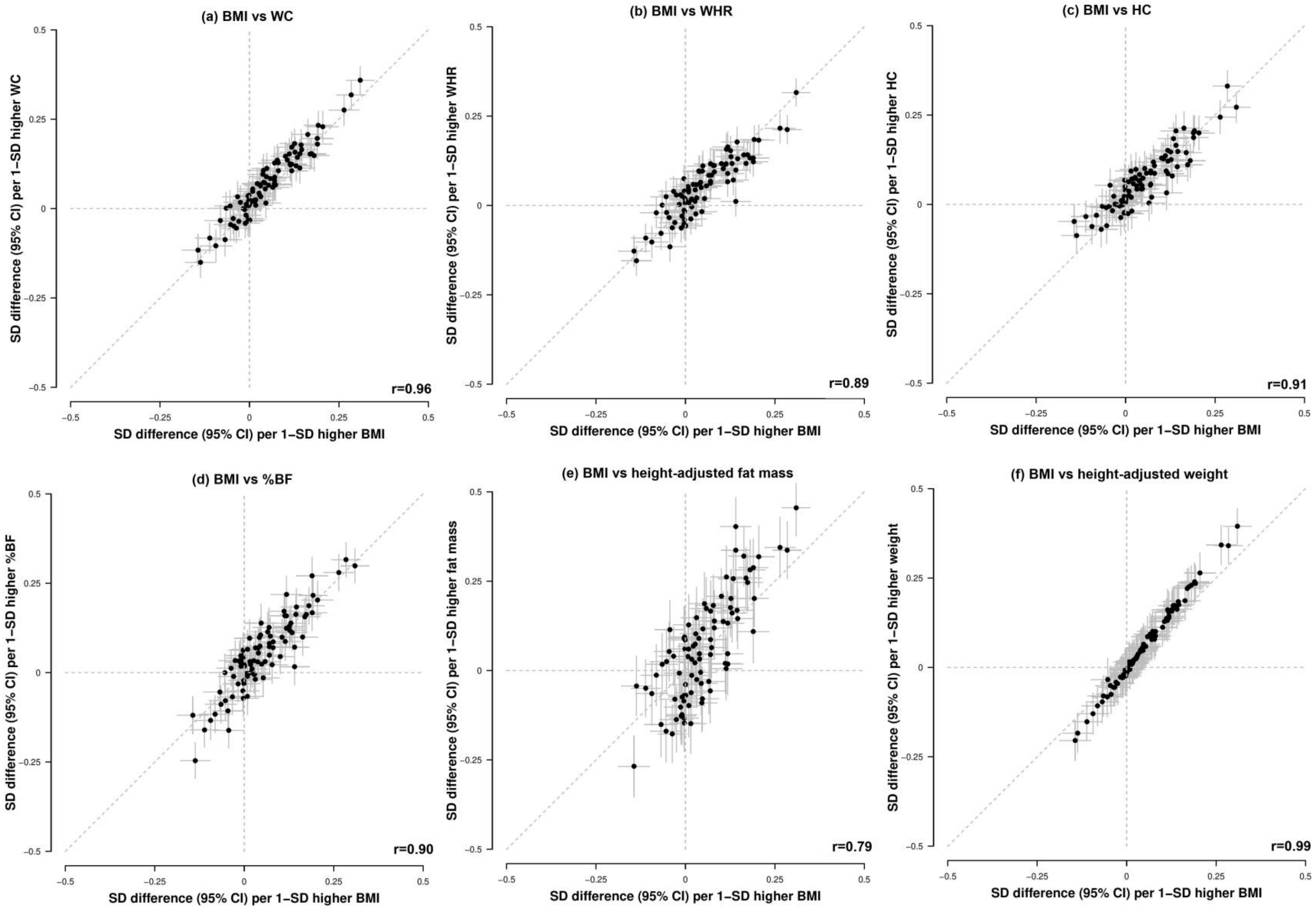

© 2020 Pang et al. *JAMA Cardiology.*

eFigure 7. Global comparison of 1-SD differences of 90 protein biomarkers associated with 1-SD higher observational BMI *versus* genetically elevated BMI

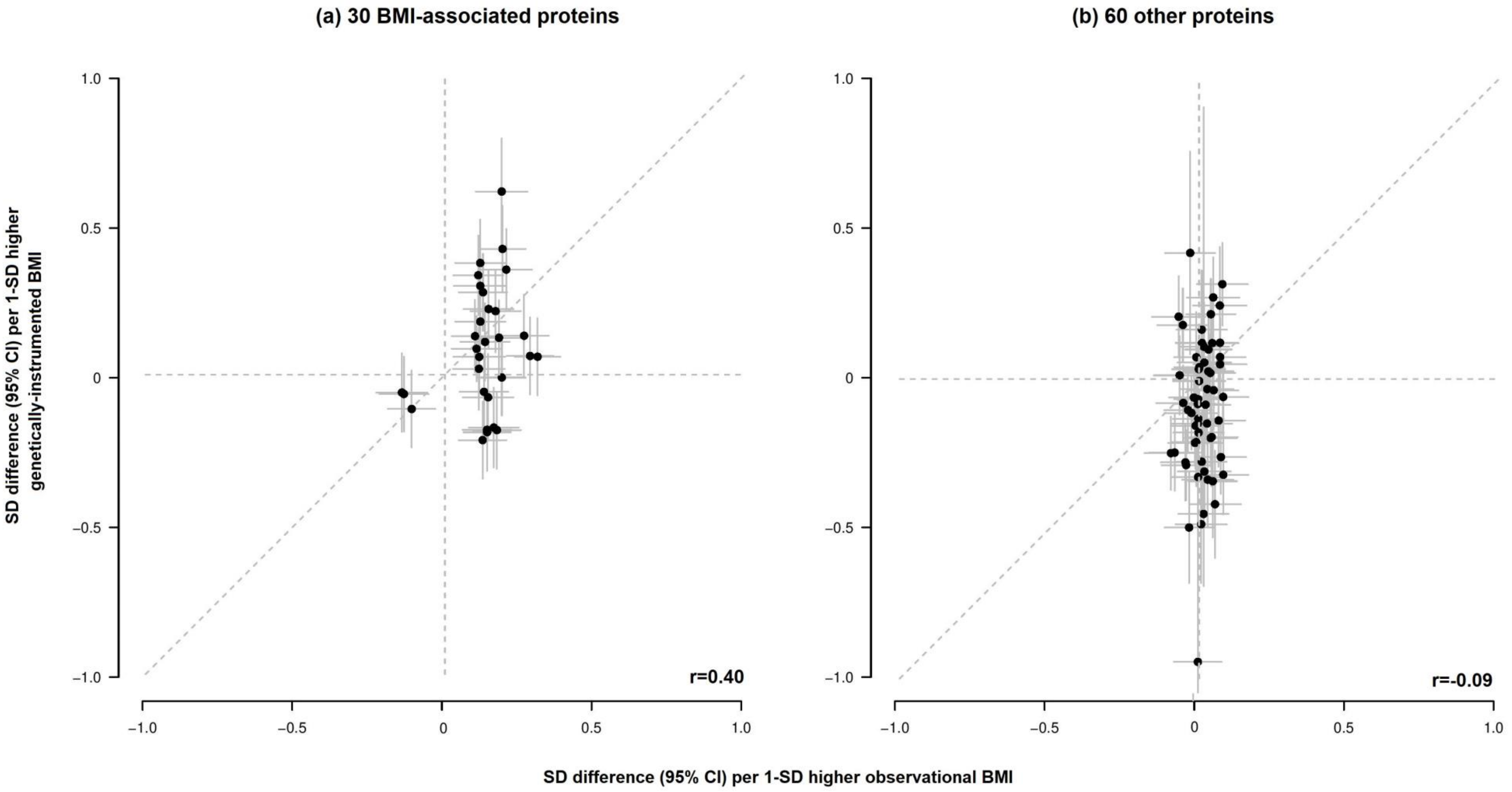

Panel (a) shows the 30 protein biomarkers which showed observational associations with BMI at FDR <5%, panel (b) shows the 60 other proteins.

© 2020 Pang et al. *JAMA Cardiology.*

eFigure 8. Meta-analysis of causal associations of BMI with selected protein biomarkers

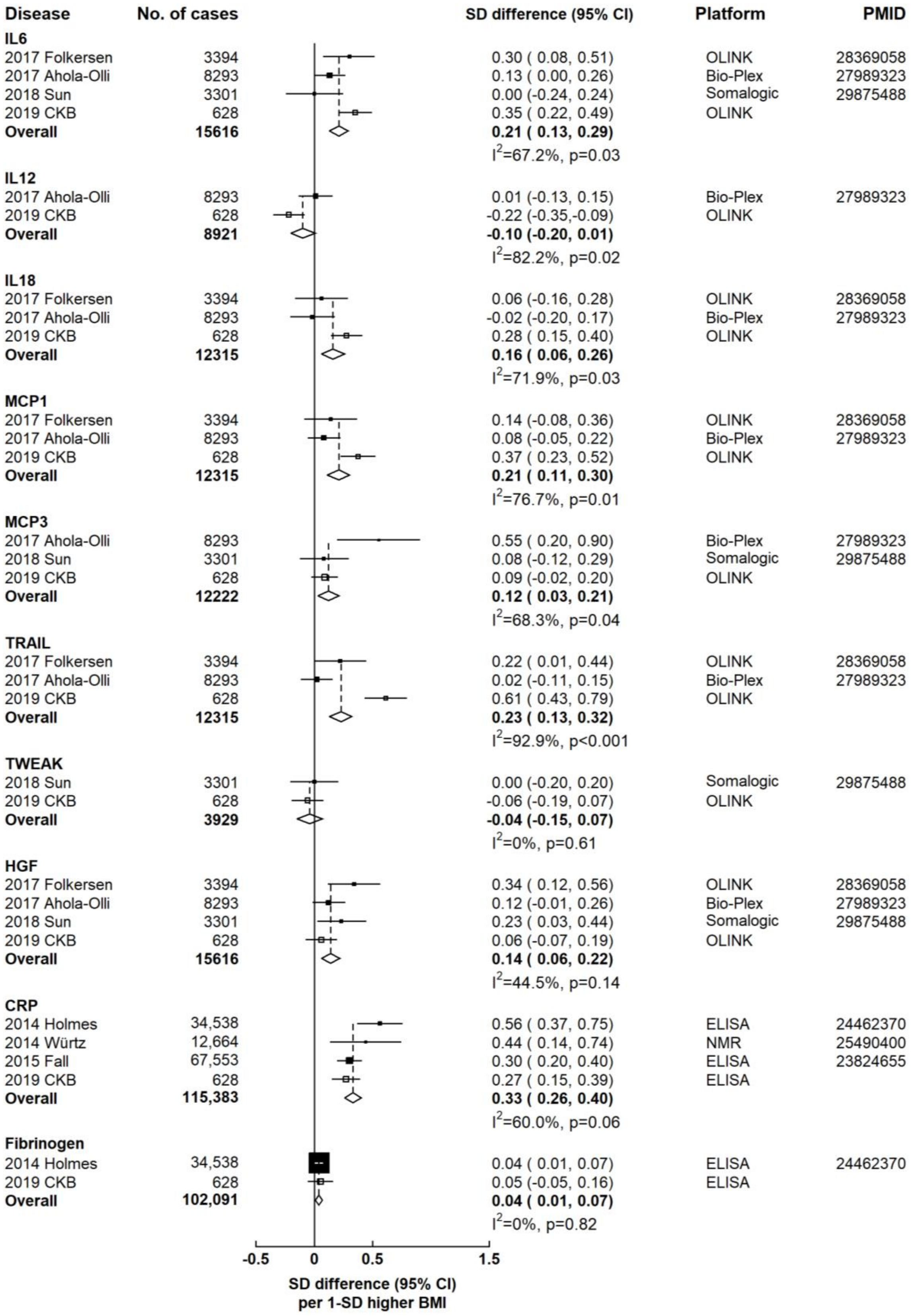

© 2020 Pang et al. *JAMA Cardiology.*

eFigure 9. Meta-analysis of genetic associations of BMI with proteins and of proteins with CVD

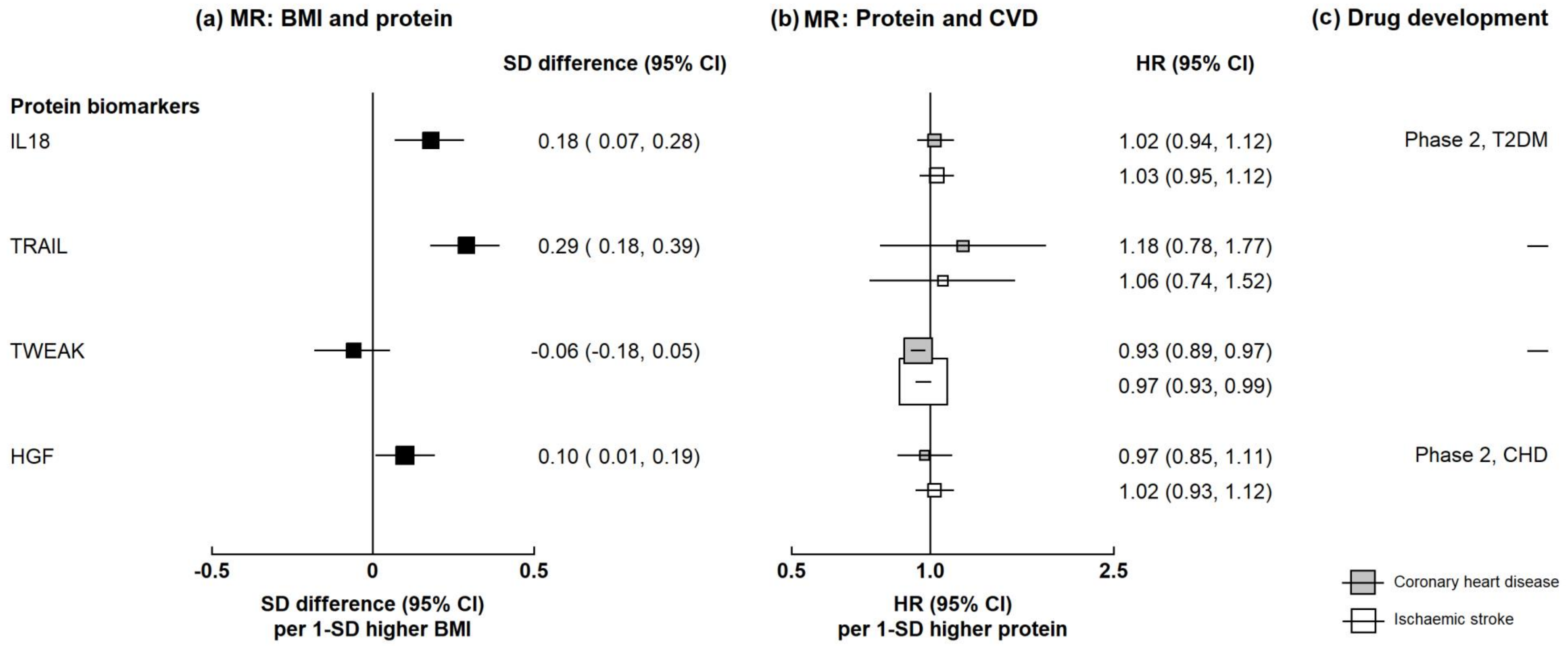

Column (a) shows genetic associations of BMI with selected proteins. Column (b) shows genetic associations of selected proteins with CVD (**eMethods**). Column (c) shows the stage of drug development for selected proteins. Abbreviations: CHD, coronary heart disease; T2DM, type 2 diabetes mellitus.

© 2020 Pang et al. *JAMA Cardiology.*

eFigure 10. Associations of *cis* and *trans* SNP in relation to protein biomarkers and CVD

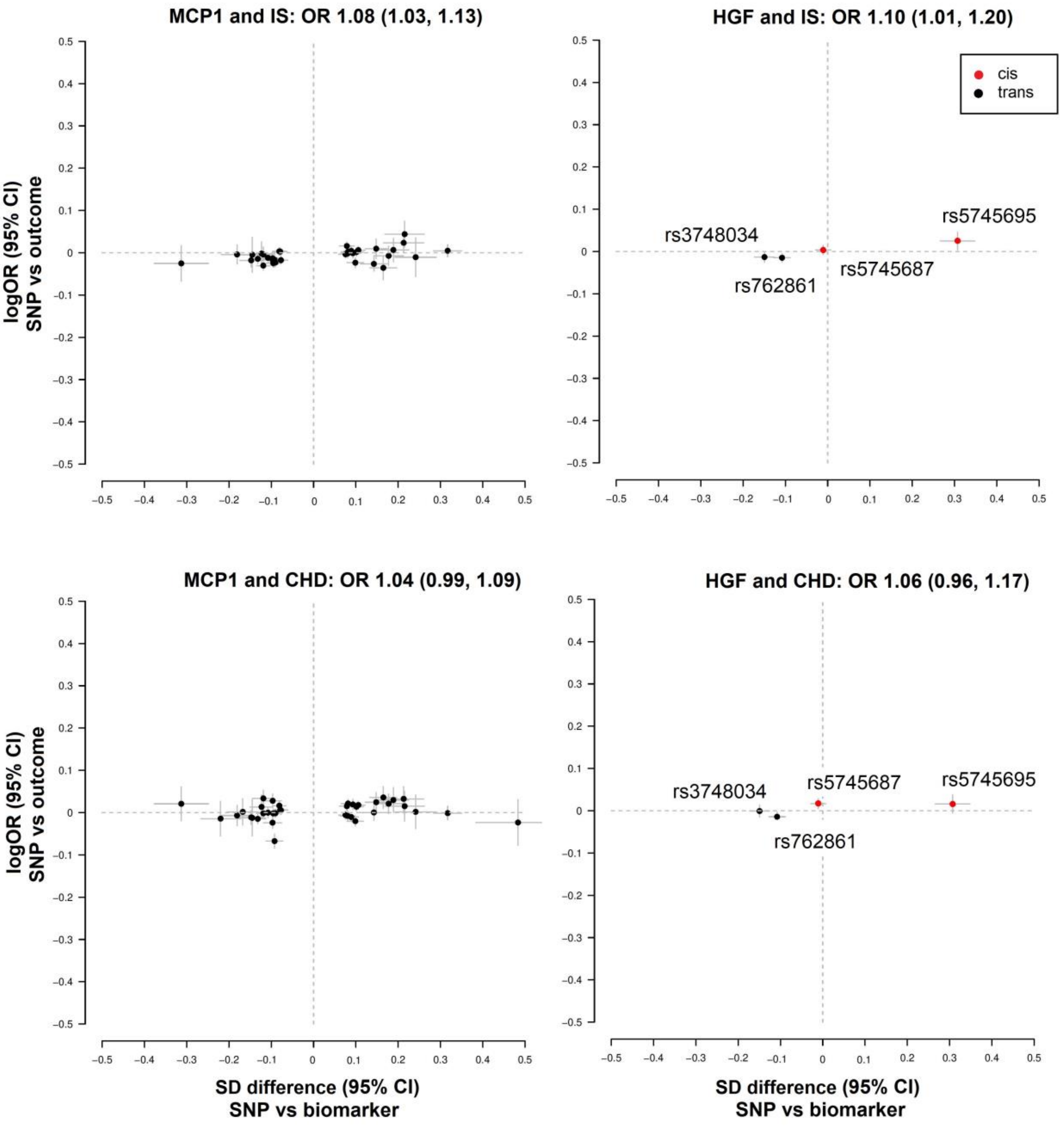

Abbreviations: IS, ischaemic stroke; CHD, coronary heart disease; OR, odds ratio.

© 2020 Pang et al. *JAMA Cardiology.*

eFigure 10. continued

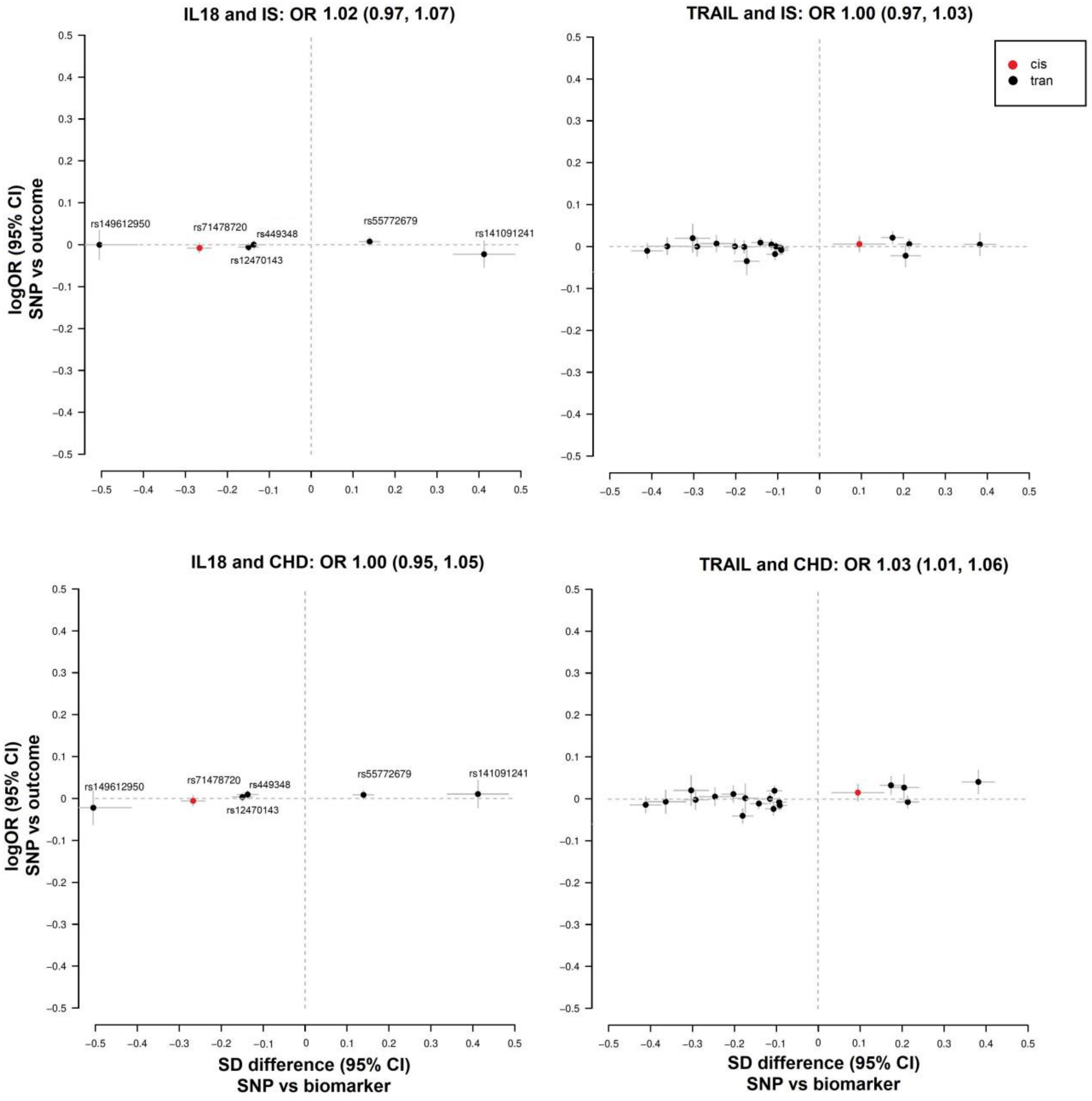

Abbreviations: IS, ischaemic stroke; CHD, coronary heart disease; OR, odds ratio.

© 2020 Pang et al. *JAMA Cardiology.*

eFigure 11. Colocalisation analysis of TWEAK, HGF, and ischaemic stroke

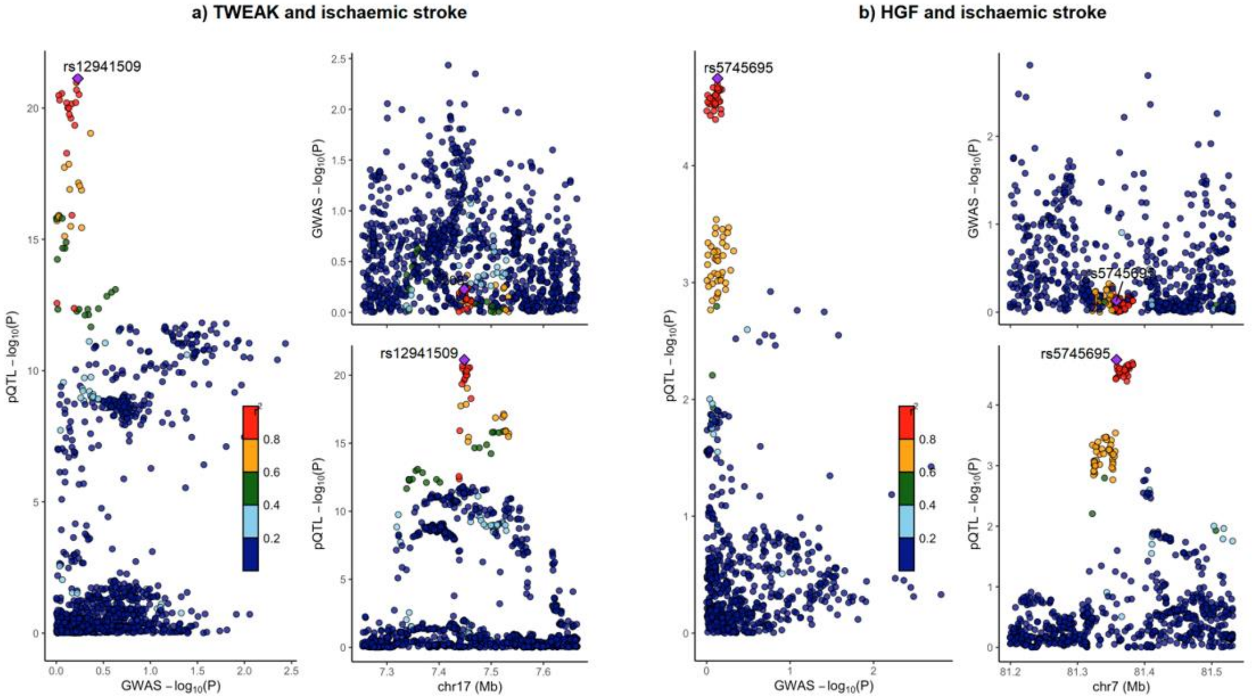

	TWEAK	HGF
**Lead SNP**	rs12941509	rs5745695
**No. of SNPs**	1125	792
**Size of the window**	chr17: 7452416-7464918	chr7:81331444-81399452
**PP for H0-H4**		
H0: No causal variant for either trait	$1.12\text{x}10^{-42}$	$6.30\text{x}10^{-7}$
H1: Causal variant for protein trait only	0.973	0.968
H2: Causal variant for ischaemic stroke only	$2.40\text{x}10^{-44}$	$1.77\text{x}10^{-8}$
H3: Two distinct causal variants	0.021	0.027
H4: One common causal variant	0.0059	0.0053

Abbreviation: H, hypothesis; PP, posterior probabilities.

© 2020 Pang et al. *JAMA Cardiology.*

eFigure 12. Genetic associations of BMI with proteomics comparing weighted and unweighted GRS

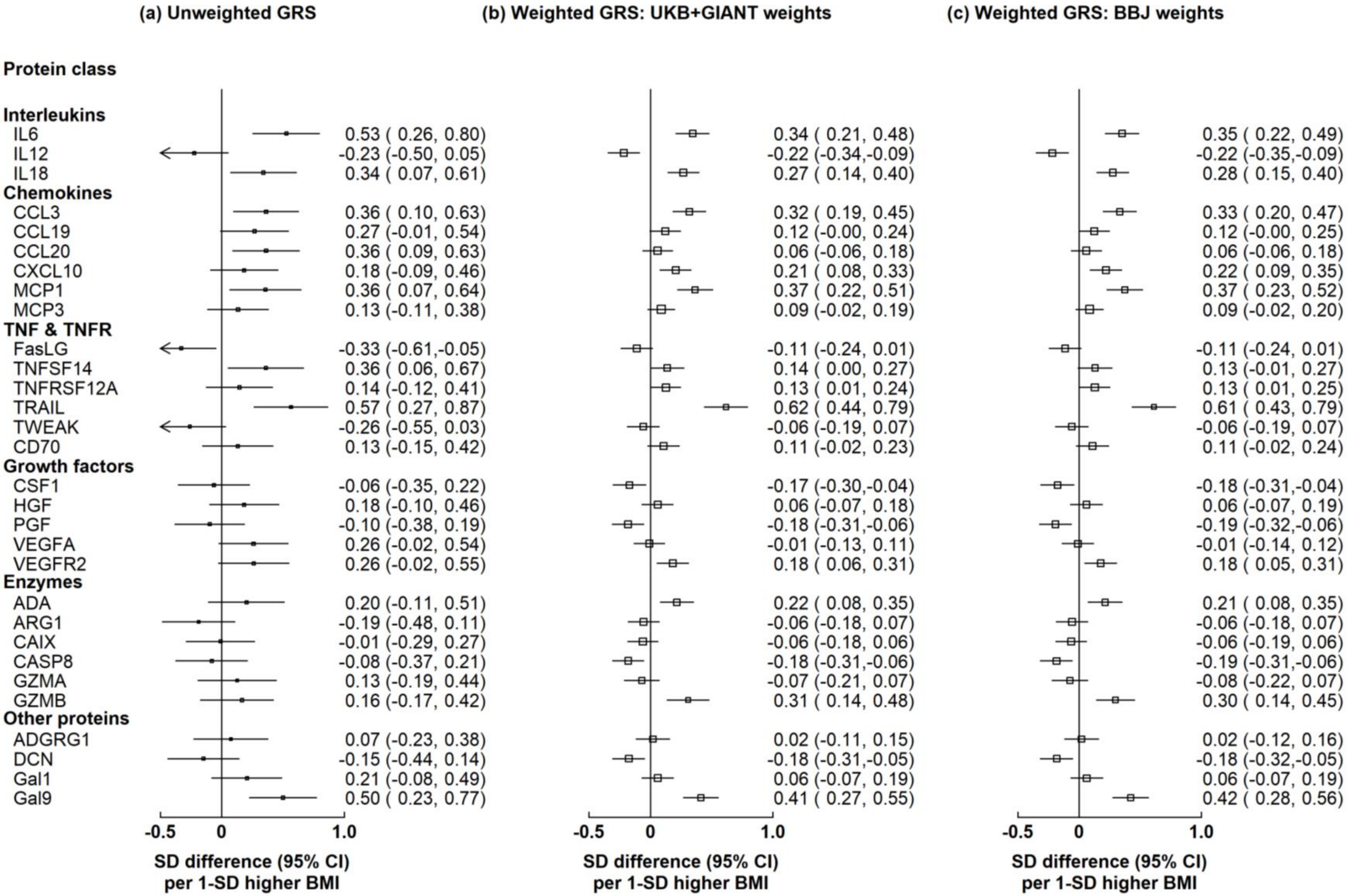

© 2020 Pang et al. *JAMA Cardiology.*

eFigure 13. Genetic associations of BMI with proteomics using different external weights

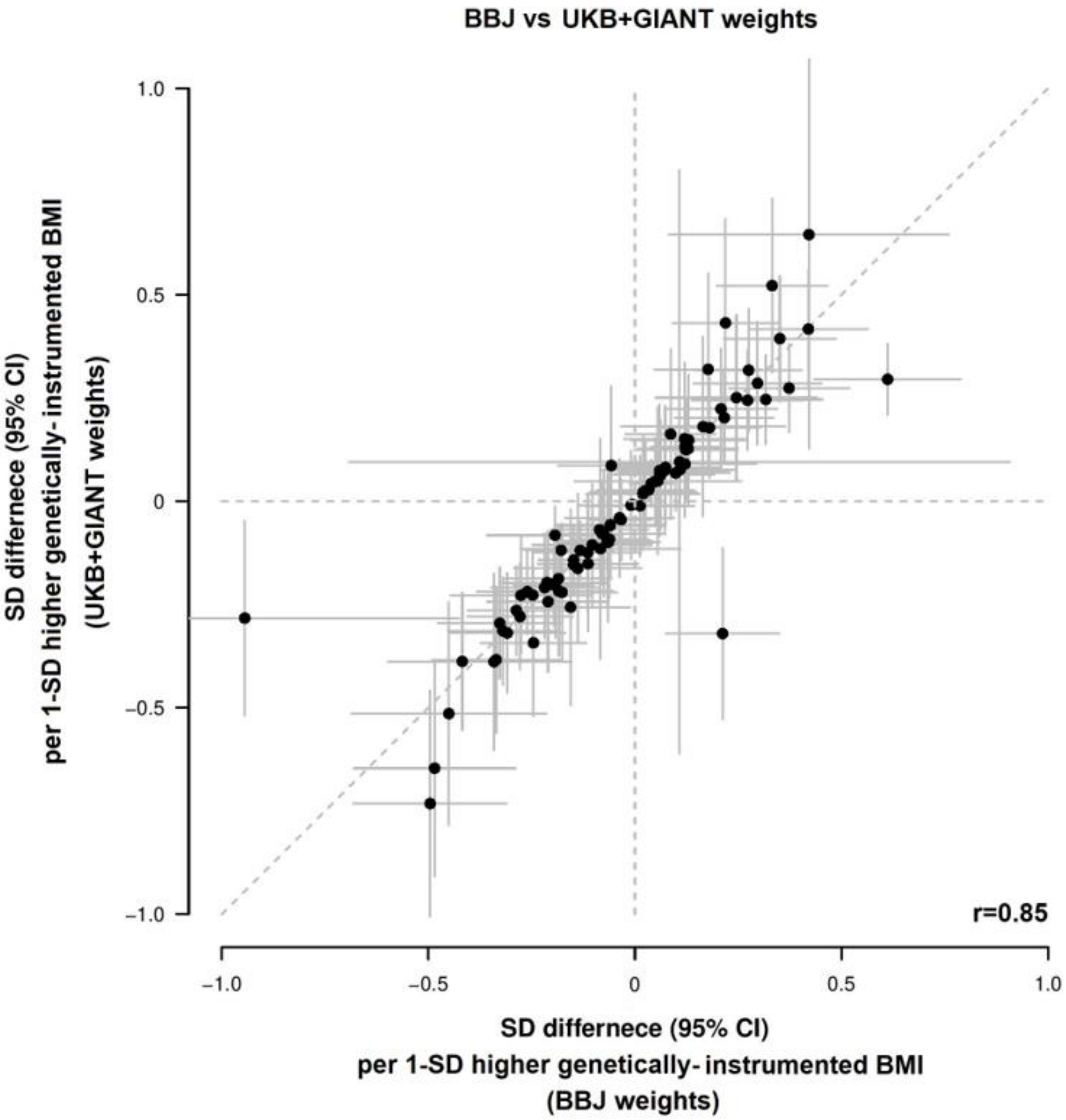

© 2020 Pang et al. *JAMA Cardiology.*

eFigure 14. Associations of BMI with proteomics by population subgroup

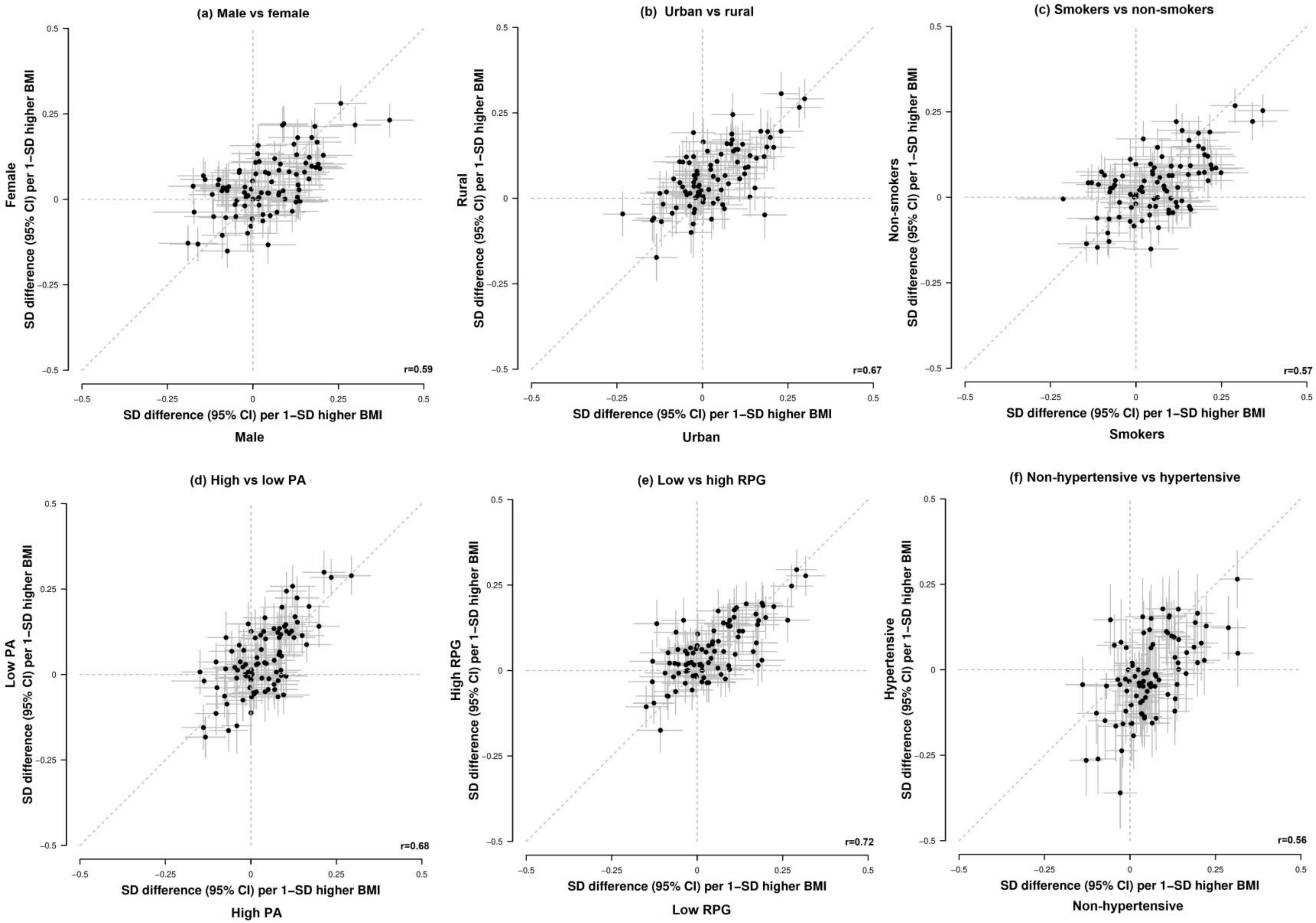

The basic model was adjusted for age at baseline, age squared, sex, area, education, household income, alcohol, self-rated health, systolic blood pressure (SBP), diabetes, statin treatment, prior kidney disease, and fasting time, where appropriate.

© 2020 Pang et al. *JAMA Cardiology.*

eFigure 15. Observational associations of protein biomarkers with CVD

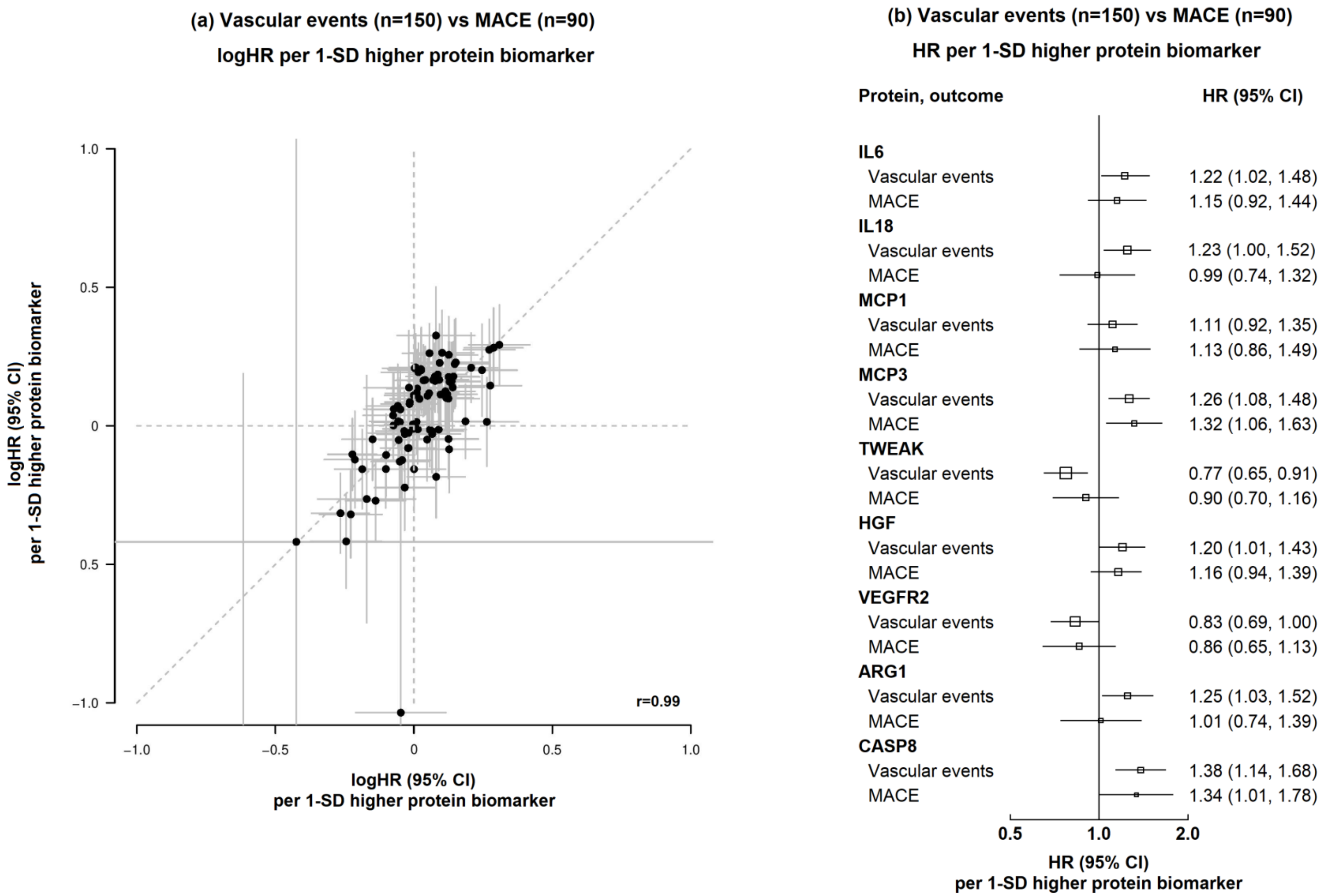

Abbreviation: MACE, major adverse coronary events.

© 2020 Pang et al. *JAMA Cardiology.*

eFigure 16. Causal associations of BMI and proteomics using different MR methods

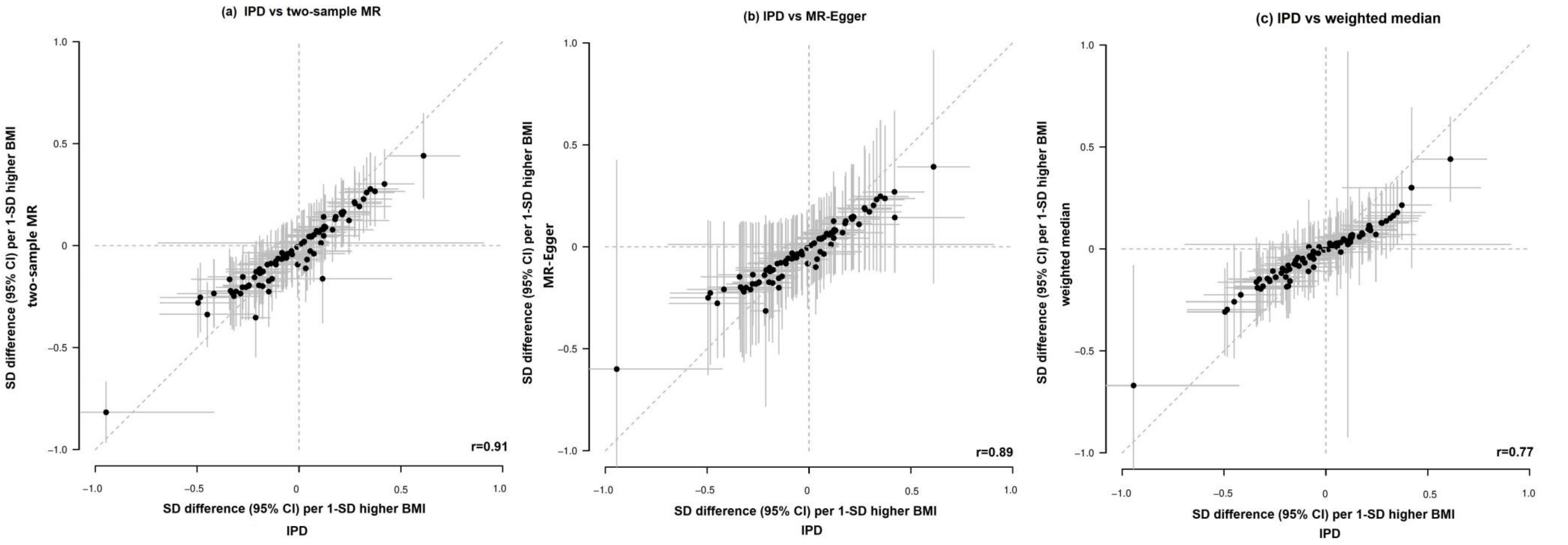

Abbreviation: IPD, individual participant-level data.

© 2020 Pang et al. *JAMA Cardiology.*

eFigure 17. Sensitivity analyses of genetic associations of BMI and proteomics in CKB

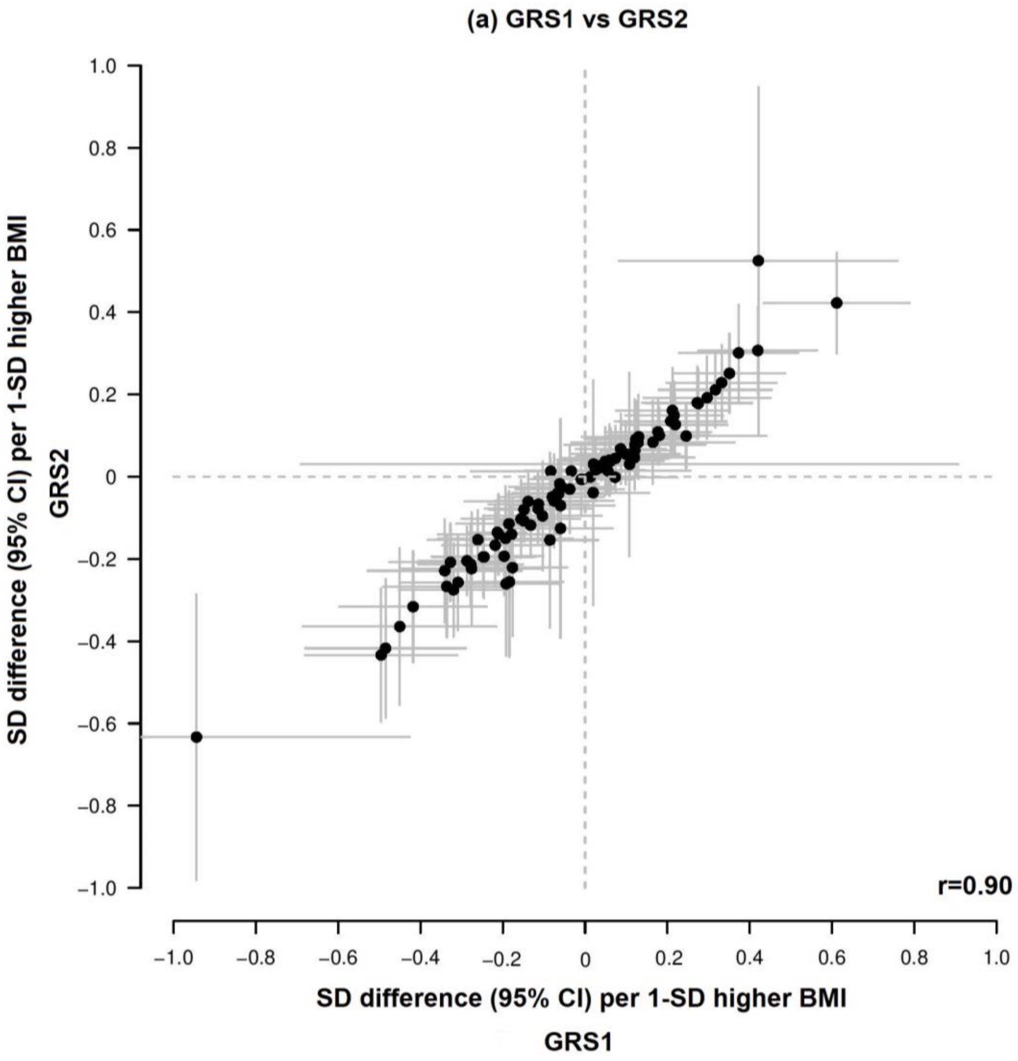

GRS1 refers to the genetic score comprising 571 SNPs. GRS2 refers to the genetic score excluding 51 SNPs within 1M bp of genes encoding protein biomarkers.

© 2020 Pang et al. *JAMA Cardiology.*

eFigure 18. Central illustration of BMI, protein biomarkers, and risk of CVD

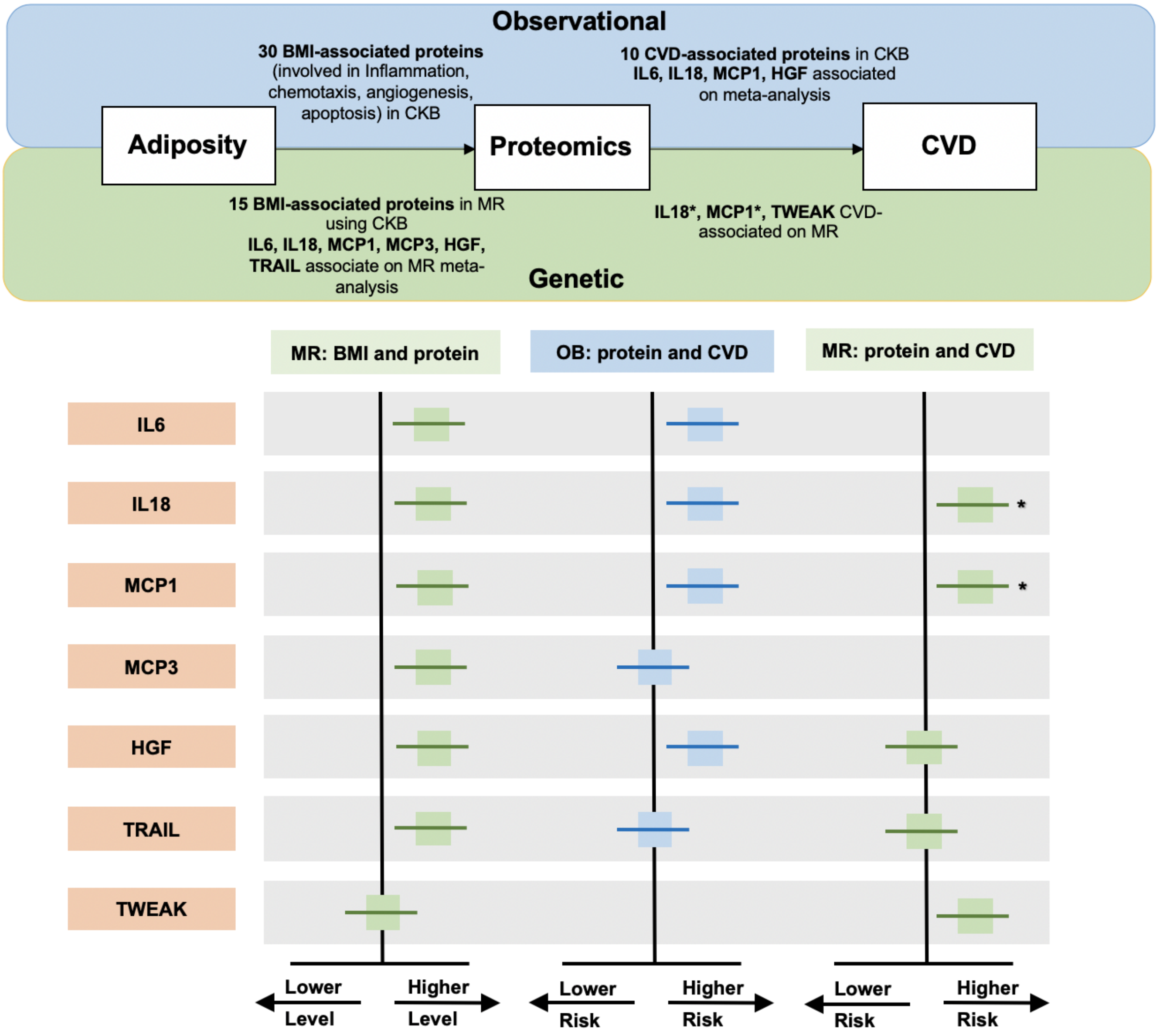

We assessed the associations of BMI with 90 protein biomarkers and of these protein biomarkers with risk of CVD. For the associations between BMI and protein biomarkers, the study showed that measured BMI was observationally associated with 30 protein biomarkers involved in inflammation, chemotaxis, angiogenesis, and apoptosis, with general concordance between the observational and genetic associations. When meta-analysed with MR estimates calculated form previous GWAS studies, the genetic associations were most robust for IL6, IL18, MCP1, MCP3, HGF, and TRAIL. For the associations between protein biomarkers and CVD, ten showed nominal observational associations with risk of CVD among the 30 BMI-associated proteins. When meta-analysed with previous prospective studies, observational associations were identified for IL6, IL18, MCP1, and HGF. Using available instruments in European populations, there was some evidence of genetic associations for MCP1 and TWEAK with CVD. The lower panel illustrates the associations of BMI with selected proteins demonstrating evidence of a potentially causal effect (other than for TWEAK), the observational association of these proteins with risk of CVD, and where possible, the MR estimate with CVD. Note that for IL18 while no association with CVD was seen we identified a potential association with type 2 diabetes. For MCP1, the MR was driven entirely by *trans*-pQTL.

© 2020 Pang et al. *JAMA Cardiology.*

eTable 1. List of 92 proteomics quantified by the OLINK immuno-oncology assay

Label	Name	Biological process			
ADA	Adenosine deaminase	M			V
ADGRG1	Adhesion G-protein coupled receptor G1				
ANG1	Angiopoietin-1				V
ANGPT2	Angiopoietin-2				V
ARG1	Arginase-1				
CAIX	Carbonic anhydrase IX	M			V
CASP8	Caspase-8	A			
CCL3	C-C motif chemokine 3	C			
CCL4	C-C motif chemokine 4	C			
CCL17	C-C motif chemokine 17	C		S	
CCL19	C-C motif chemokine 19	C		S	
CCL20	C-C motif chemokine 20	C		S	
CCL23	C-C motif chemokine 23	C			V
CD4	T-cell surface glycoprotein CD4		P	S	
CD5	T-cell surface glycoprotein CD5		P	S	
CD8A	T-cell surface glycoprotein CD8 alpha chain		P		
CD27	CD27 antigen		P		
CD28	T-cell surface glycoprotein CD28		P		
CD40	CD40L receptor		P		
CD40L	CD40-L	A	P		
CD70	CD70 antigen		P		
CD83	CD83 antigen		P		
CD244	Natural killer cell receptor 2B4		P		
CRTAM	Cytotoxic and regulatory T-cell molecule		P		
CSF1	Macrophage colony-stimulating factor 1			S	
CX3CL1	Fractalkine	C	P		
CXCL1	C-X-C motif chemokine 1	C		S	V
CXCL5	C-X-C motif chemokine 5	C		S	V
CXCL9	C-X-C motif chemokine 9	C	P		V
CXCL10	C-X-C motif chemokine 10	C	P		V
CXCL11	C-X-C motif chemokine 11	C	P	S	V
CXCL12	Stromal cell-derived factor 1	C		S	V
CXCL13	C-X-C motif chemokine 13	C	P	S	
DCN	Decorin				V
EGF	Pro-epidermal growth factor				V
FasL	Fas antigen ligand	A			
FGF2	Fibroblast growth factor 2				V
Gal1	Galectin-1			S	V
Gal9	Galectin-9	A		S	V
GZMA	Granzyme A	A			
GZMB	Granzyme B	A			
GZMH	Granzyme H	A			
HGF	Hepatocyte growth factor				V
HO1	Heme oxygenase 1	M			
ICOSLG	ICOS ligand		P		

© 2020 Pang et al. *JAMA Cardiology.*

Label	Name	Biological process			
IFN beta	Interferon beta		P		
IFN gamma	Interferon gamma		P		
IL1 alpha	Interleukin-1 alpha		P	S	V
IL2	Interleukin-2		P		
IL4	Interleukin-4			S	
IL5	Interleukin-5			S	
IL6	Interleukin-6		P	S	
IL7	Interleukin-7		P		
IL8	Interleukin-8	C		S	V
IL10	Interleukin-10			S	
IL12	Interleukin-12				
IL12RB1	Interleukin-12 receptor subunit beta-1		P		
IL13	Interleukin-13			S	
IL18	Interleukin-18		P	S	
IL21	Interleukin-21		P		
IL33	Interleukin-33			S	
IL35	Interleukin-35			S	
KLRD1	Natural killer cells antigen CD94		P		
LAMP3	Lysosome-associated membrane glycoprotein 3			S	
LAP TGF-beta-1	Latency-associated peptide transforming growth factor beta-1			S	
MCP1	Monocyte chemotactic protein 1	C			V
MCP2	Monocyte chemotactic protein 2	C			
MCP3	Monocyte chemotactic protein 3	C			
MCP4	Monocyte chemotactic protein 4	C		S	V
MIC-A/B	MHC class I polypeptide-related sequence A/B			S	
MMP7	Matrix metalloproteinase-7	A		S	
MMP12	Matrix metalloproteinase-12			S	V
NCR1	Natural cytotoxicity triggering receptor 1		P		
NOS3	Nitric oxide synthase, endothelial				V
PD L1	Programmed cell death 1 ligand 1			S	
PD L2	Programmed cell death 1 ligand 2			S	
PDCD1	Programmed cell death protein 1			S	
PDGF subunit B	Platelet-derived growth factor subunit B				V
PGF	Placenta growth factor				V
PTN	Pleiotrophin				V
TIE2	Angiopoietin-1 receptor				V
TNF	Tumour necrosis factor alpha		P	S	V
TNFRSF4	Tumour necrosis factor receptor superfamily member 4		P		
TNFRSF9	Tumour necrosis factor receptor superfamily member 9		P		
TNFRSF12A	Tumour necrosis factor receptor superfamily member 12A	A			V
TNFRSF21	Tumour necrosis factor receptor superfamily member 21	A			
TNFSF14	Tumour necrosis factor ligand superfamily member 14		P		
TRAIL	TNF-related apoptosis-inducing ligand	A			
TWEAK	Tumour necrosis factor (Ligand) superfamily, member 12	A			V
VEGFA	Vascular endothelial growth factor A				V

© 2020 Pang et al. *JAMA Cardiology.*

Label	Name	Biological process
VEGFC	Vascular endothelial growth factor C	V
VEGFR2	Vascular endothelial growth factor receptor 2	V

Abbreviations: A, apoptosis/cell killing; C, chemotaxis; M, metabolism/autophagy; P, promote tumour immunity; S, supress tumour immunity; V, vascular & tissue remodelling.

© 2020 Pang et al. *JAMA Cardiology.*

eTable 2. Grouping of the 92 proteins measured by OLINK Immuno-Oncology assay

Label, group	Name	Protein type
**Chemokines**		
CCL3	C-C motif chemokine 3	Chemokine CC family
CCL4	C-C motif chemokine 4	Chemokine CC family
CCL17	C-C motif chemokine 17	Chemokine CC family
CCL19	C-C motif chemokine 19	Chemokine CC family
CCL20	C-C motif chemokine 20	Chemokine CC family
CCL23	C-C motif chemokine 23	Chemokine CC family
MCP1/CCL2	Monocyte chemotactic protein 1	Chemokine CC family
MCP2/CCL8	Monocyte chemotactic protein 2	Chemokine CC family
MCP3/CCL7	Monocyte chemotactic protein 3	Chemokine CC family
MCP4	Monocyte chemotactic protein 4	Chemokine CC family
CXCL1	C-X-C motif chemokine 1	Chemokine CxC family, growth factor
CXCL5	C-X-C motif chemokine 5	Chemokine CxC family
CXCL9	C-X-C motif chemokine 9	Chemokine CxC family
CXCL10	C-X-C motif chemokine 10	Chemokine CxC family
CXCL11	C-X-C motif chemokine 11	Chemokine CxC family
CXCL12	Stromal cell-derived factor 1	Chemokine CxC family, growth factor
CXCL13	C-X-C motif chemokine 13	Chemokine CxC family
CX3CL1	Fractalkine	Intercrine delta family
**Inflammatory cytokines**		
Interleukins		
IL1 alpha	Interleukin-1 alpha	IL1 family, mitogen
IL2	Interleukin-2	IL2 family, growth factor
IL4	Interleukin-4	IL4/IL13 family, growth factor
IL5	Interleukin-5	IL5 family, growth factor
IL6	Interleukin-6	IL6 superfamily, growth factor

© 2020 Pang et al. *JAMA Cardiology.*

IL7	Interleukin-7	IL7/IL9 family, growth factor
**Label, group**	**Name**	**Protein type**
IL8	Interleukin-8	Chemokine CxC family
IL10	Interleukin-10	IL10 family
IL12	Interleukin-12	IL6 superfamily, growth factor
IL12RB1	Interleukin-12 receptor subunit beta-1	Type I cytokine receptor family, cytokine receptor
IL13	Interleukin-13	IL4/IL13 family
IL18	Interleukin-18	IL1 family
IL21	Interleukin-21	IL15/IL21 family, growth factor
IL33	Interleukin-33	IL1 family
IL35	Interleukin-35	IL12 family
Interferon		
IFN-beta	Interferon beta	Alpha/beta interferon family
IFN-gamma	Interferon gamma	Gamma interferon family
TNF(R) superfamily		
TNF	Tumor necrosis factor	TNF superfamily
TNFSF14	Tumor necrosis factor ligand superfamily member 14	TNF superfamily
TRAIL	TNF-related apoptosis-inducing ligand	TNF superfamily
TWEAK	Tumor necrosis factor (ligand) superfamily, member 12	TNF superfamily
TNFRSF4	Tumor necrosis factor receptor superfamily member 4	TNFR superfamily
TNFRSF9	Tumor necrosis factor receptor superfamily member 9	TNFR superfamily
TNFRSF12A	Tumor necrosis factor receptor superfamily member 12A	TNFR superfamily
TNFRSF21	Tumor necrosis factor receptor superfamily member 21	TNFR superfamily
CD27	CD27 antigen	TNFR superfamily
CD40	CD40 ligand receptor	TNFR superfamily
CD40L	CD40 ligand	TNF superfamily
CD70	CD70 antigen	TNF superfamily
FasLG	Fas antigen ligand	TNF superfamily

© 2020 Pang et al. *JAMA Cardiology.*

**Growth factors (receptors)**		
ANG1	Angiopoietin-1	Growth factor, VEGF family
**Label, group**	**Name**	**Protein type**
ANGPT2	Angiopoietin-2	Growth factor
CSF1	Macrophage colony-stimulating factor 1	Growth factor, cytokine
EGF	Pro-epidermal growth factor	Growth factor
FGF2	Fibroblast growth factor 2	Growth factor
HGF	Hepatocyte growth factor	Growth factor
LAP TGF-beta-1	Latency-associated peptide transforming growth factor beta-1	Growth factor, TGF beta superfamily
PDGF subunit B	Platelet-derived growth factor subunit B	Growth factor, PDGF/VEGF family
PGF	Placenta growth factor	Growth factor, PDGF/VEGF family
PTN	Pleiotrophin	Growth factor, pleiotrophin
VEGFA	Vascular endothelial growth factor A	Growth factor, VEGF family
VEGFC	Vascular endothelial growth factor C	Growth factor, VEGF family
VEGFR2	Vascular endothelial growth factor receptor 2	Growth factor, PDGF/VEGF receptor family
**Other proteins**		
Enzymes		
ADA	Adenosine deaminase	Purine metabolism, breaking down adenosine into nucleic acids
ARG1	Arginase-1	Urea metabolism, converting L-arginine to urea and L-ornithine
CAIX	Carbonic anhydrase IX	Reversible hydration of carbon dioxide
CASP8	Caspase-8	Cysteine protease, activating the TNFRSF6/FAS mediated and TNFRSF1A induced cell death
GZMA	Granzyme A	Protease, involved in granzyme-mediated apoptotic signaling pathway
GZMB	Granzyme B	Protease, involved in granzyme-mediated apoptotic signaling pathway
GZMH	Granzyme H	Protease, involved in granzyme-mediated apoptotic signaling pathway
HO1	Heme oxygenase 1	Heme catabolism, cleaving the heme ring to form biliverdin
MMP7	Matrix metalloproteinase-7	Protease, activating procollagenase
MMP12	Matrix metalloproteinase-12	Protease, with elastolytic activity

© 2020 Pang et al. *JAMA Cardiology.*

NOS3	Nitric oxide synthase, endothelial	Mediating VEGF-induced angiogenesis
Membrane proteins		
CD4	T-cell surface glycoprotein CD4	T-cell surface glycoprotein (adaptive)
CD5	T-cell surface glycoprotein CD5	T-cell surface glycoprotein (adaptive)
**Label, group**	**Name**	**Protein type**
CD8A	T-cell surface glycoprotein CD8 alpha chain	T-cell surface glycoprotein (adaptive)
CD28	T-cell surface glycoprotein CD28	T-cell surface glycoprotein (adaptive)
CD83	CD83 antigen	Dendritic cell surface protein (innate)
CD244	Natural killer cell receptor 2B4	Glycoprotein receptor on NK, monocytes, basophils (innate)
CRTAM	Cytotoxic and regulatory T-cell molecule	T-cell molecule (innate and adaptive)
ICOSLG	ICOS ligand	T-cell surface receptor ICOS (adaptive)
KLRD1	Natural killer cells antigen CD94	Receptor on NK cells and some cytotoxic T-cells (for MHC class MHC class I HLA-E molecules) (innate and adaptive)
LAMP3	Lysosome-associated membrane glycoprotein 3	Lysosome membrane protein (adaptive immunity)
NCR1	Natural cytotoxicity triggering receptor 1	Cell surface receptor (innate immunity)
MIC-A/B	MHC class I polypeptide-related sequence A/B	MHC class I, expressed on the cell surface of all nucleated cells and also platelets (widely expressed)
ADGRG1	Adhesion G-protein coupled receptor G1	Receptor expressed in liver, muscle, neural, cytotoxic lymphoid cells (widely expressed)
TIE2	Angiopoietin-1 receptor	Enzyme/ cell surface protein (widely expressed)
PD-L1	Programmed cell death 1 ligand 1	Ligand of the inhibitory receptor PD-1 (adaptive immunity)
PD-L2	Programmed cell death 1 ligand 2	Ligand of the inhibitory receptor PD-2 (adaptive immunity)
PDCD1	Programmed cell death protein 1	Cell surface receptor (adaptive immunity)
Extracellular proteins		
Gal-1	Galectin-1	Galectin
Gal-9	Galectin-9	Galectin
DCN	Decorin	Proteoglycan

© 2020 Pang et al. *JAMA Cardiology.*

eTable 3. Limit of detection and coefficient of variation of 92 protein biomarkers

Protein	Limit of detection (LOD)	% below LOD	Coefficient of variation (CV, %)*		Mean	SD
	NPX units		Inter-assay	Intra-assay	NPX units	
ADA	0.27	0	8.40	7.05	2.41	0.35
ADGRG1	1.15	67.40	12.53	10.81	1.30	0.41
ANG1	0.80	0	15.63	4.37	6.77	1.38
ANGPT2	0.62	0	8.18	5.99	3.80	0.42
ARG1	1.59	11.60	10.26	5.87	2.60	0.80
CAIX	0.15	0	9.77	5.01	3.37	0.63
CASP8	1.30	0	13.52	4.37	3.59	0.69
CCL3	0.93	0	7.91	6.06	4.58	0.59
CCL4	0.65	0	11.14	6.50	6.78	0.62
CCL17	0.48	0	12.53	6.11	6.17	1.11
CCL19	1.10	0	8.83	5.85	8.52	0.82
CCL20	1.22	0	13.00	7.04	6.39	1.14
CCL23	0.34	0	10.62	6.60	9.34	0.49
CD4	-1.61	0	8.54	4.93	0.10	0.34
CD5	-0.07	0	6.40	5.72	4.35	0.41
CD8A	0.78	0	13.06	6.50	8.60	0.81
CD27	0.39	0	10.66	6.05	7.52	0.39
CD28	0.54	5.00	6.77	6.37	0.90	0.30
CD40	0.81	0	8.19	5.11	9.88	0.39
CD40L	1.51	0.20	14.57	7.35	3.53	1.07
CD70	1.07	0	8.40	6.38	2.73	0.37
CD83	0.11	0	8.16	6.25	1.73	0.34
CD244	1.48	0	7.67	6.67	5.49	0.33
CRTAM	0.75	0	11.12	6.67	4.06	0.55
CSF1	1.01	0	7.82	6.05	7.00	0.24
CX3CL1	1.04	0	7.56	5.27	5.42	0.40
CXCL1	0.99	0	13.14	4.85	7.52	1.35
CXCL5	1.56	0	26.92	7.09	9.04	2.04
CXCL9	0.72	0	12.17	6.24	6.94	0.96
CXCL10	0.88	0	12.41	6.84	7.46	0.81
CXCL11	0.79	0	9.98	7.77	6.27	0.93
CXCL12	0.92	46.20	14.33	11.25	1.04	0.23
CXCL13	1.38	0	9.68	6.41	8.26	0.59
DCN	0.37	0	11.18	6.04	3.81	0.26
EGF	0.29	0	15.54	5.16	5.09	1.37
FasL	0.48	0	7.52	5.68	5.92	0.44
FGF2	0.48	82.60	12.06	10.07	0.56	0.30
Gal1	1.54	0	9.16	6.64	5.64	0.25
Gal9	0.61	0	4.46	3.11	7.00	0.34
GZMA	0.99	0.30	8.34	5.80	4.91	0.50
GZMB	0.76	0	7.14	5.44	3.09	0.66
GZMH	1.60	0	10.04	5.71	4.37	0.69
HGF	0.61	0	7.48	5.39	7.03	0.41
HO1	1.43	0	8.44	6.74	11.53	0.42
ICOSLG	0.90	0	7.74	5.44	4.57	0.35
IFN beta	0.44	97.80	11.10	7.45	0.46	0.15
IFN gamma	0.76	98.00	9.28	7.61	0.77	0.07
IL1 alpha	0.42	99.10	8.55	5.46	0.45	0.35
IL2	0.92	99.80	19.76	16.30	0.92	0.001
IL4	1.00	98.60	14.48	11.19	1.01	0.15
IL5	2.20	70.70	13.83	10.65	2.68	1.10

© 2020 Pang et al. *JAMA Cardiology.*

Protein	LOD	% LOD	Coefficient of variation (CV, %)*		Mean	SD
	NPX units		Inter-assay	Intra-assay	NPX units	
IL6	1.12	0	13.13	8.07	2.59	0.84
IL7	0.92	0	9.60	3.16	3.36	0.99
IL8	1.03	0	12.29	5.75	4.31	0.87
IL10	1.47	7.40	12.84	10.79	1.98	0.46
IL12	1.30	0	13.88	5.60	5.48	0.71
IL12RB1	1.10	1.30	9.91	7.71	1.71	0.34
IL13	1.43	99.20	24.12	21.75	1.45	0.24
IL18	1.64	0	9.70	6.48	8.07	0.56
IL21	1.44	99.80	13.91	8.81	1.44	--
IL33	0.93	97.80	10.45	8.34	0.94	0.07
IL35	2.46	99.00	26.02	22.44	2.46	--
KLRD1	0.72	0	7.90	5.49	5.03	0.53
LAMP3	1.04	0	10.70	6.52	3.75	0.70
LAP TGF-beta-1	-0.71	0	7.96	5.96	0.38	0.37
MCP1	0.66	0	10.49	6.52	9.21	0.51
MCP2	0.90	0	11.55	6.21	6.49	0.92
MCP3	1.22	32.90	12.66	10.37	1.49	0.34
MCP4	0.13	0	11.51	7.58	6.54	0.82
MIC-A/B	0.55	23.40	8.17	5.06	2.94	1.47
MMP12	0.48	0	9.71	5.04	6.81	0.69
MMP7	1.56	0.80	13.41	6.52	8.43	1.44
NCR1	1.70	0	8.57	7.14	3.28	0.43
NOS3	1.38	59.70	16.03	10.75	1.48	0.18
PD L1	1.80	0	12.00	9.02	4.08	0.47
PD L2	0.73	0.20	14.50	7.47	1.61	0.29
PDCD1	1.43	0.20	10.21	9.06	2.73	0.59
PDGF subunit B	0.16	0	9.38	6.90	8.78	1.25
PGF	0.70	0	9.80	6.78	7.68	0.35
PTN	0.08	27.90	18.50	10.34	0.64	0.72
TIE2	0.55	0	6.71	5.66	6.89	0.28
TNF	1.80	94.20	12.80	10.43	1.88	0.53
TNFRSF4	1.55	0	9.79	6.05	2.64	0.37
TNFRSF9	-0.39	0	7.77	5.86	4.65	0.47
TNFRSF12A	0.50	0	10.93	8.15	5.20	0.49
TNFRSF21	0.14	0	6.62	4.44	7.22	0.24
TNFSF14	0.31	0	7.70	5.07	2.47	0.52
TRAIL	0.16	0	7.87	5.18	7.28	0.34
TWEAK	-0.42	0	8.01	5.89	7.99	0.32
VEGFA	0.76	0	9.08	5.75	7.46	0.43
VEGFC	-0.28	27.70	14.91	12.70	0.26	0.61
VEGFR2	0.07	0	7.32	5.00	6.34	0.27

* Coefficient of variation was calculated using 2^NPX.

© 2020 Pang et al. *JAMA Cardiology.*

eTable 4. Genetic variants associated with BMI in the GIANT consortium

SNP	UKB+GIANT			CKB (orientated to BMI-increasing allele in CEU)					BBJ (orientated to BMI-increasing allele in CEU)			
	BMI-increasing allele	EAF		EAF	SD difference	SE	*p*-value		EAF	SD difference	SE	*p*-value
rs1000096	C	0.38		0.19	0.001	0.006	0.913		0.17	-0.004	0.005	0.443
rs10002111	A	0.22		0.04	0.011	0.011	0.326		0.06	0.012	0.007	0.108
rs1000940	G	0.28		0.38	0.004	0.004	0.38		0.58	0.009	0.004	0.016
rs10044136	G	0.57		0.03	-0.009	0.012	0.447		0.03	0.011	0.011	0.355
rs1006893	C	0.76		0.71	-0.019	0.005	4.54E-05		0.76	0.016	0.004	<5E-08
rs1009188	T	0.71		0.86	0.011	0.006	0.0682		0.83	-0.024	0.005	<5E-08
rs10092723	C	0.25		0.23	-0.004	0.005	0.387		0.26	0.005	0.004	0.275
rs10099330	G	0.54		0.5	-0.011	0.004	0.0113		0.54	0.001	0.004	0.812
rs10101364	T	0.68		0.99	0.021	0.02	0.28		0.98	-0.022	0.013	0.086
rs10116186	G	0.46		0.79	-0.007	0.005	0.161		0.80	0.010	0.004	0.029
rs10132280	C	0.31		0.09	-0.024	0.008	0.00157		0.07	0.018	0.007	0.01
rs10197031	C	0.27		0.41	-0.014	0.005	0.00235		0.54	0.009	0.004	0.015
rs10198345	C	0.66		0.75	0.006	0.005	0.213		0.72	-0.001	0.004	0.736
rs10203386	A	0.45		0.43	0.034	0.004	3.78E-15		0.47	-0.023	0.004	<5E-08
rs10269783	A	0.42		0.6	0.013	0.004	0.00208		0.58	-0.015	0.004	<5E-08
rs1030015	T	0.54		0.34	0.012	0.004	0.0062		0.38	-0.005	0.004	0.205
rs1031670	C	0.52		0.47	-0.008	0.004	0.0593		0.58	0.000	0.004	0.89
rs1035010	T	0.25		0.29	0.007	0.005	0.125		0.28	-0.023	0.004	<5E-08
rs1038088	G	0.49		0.68	-0.014	0.005	0.00265		0.68	0.016	0.004	<5E-08
rs10402145	A	0.3		0.13	0.005	0.006	0.413		0.17	0.004	0.005	0.366
rs1045411	C	0.28		0.2	-0.009	0.005	0.0984		0.15	0.002	0.005	0.736
rs1048303	C	0.58		0.43	-0.009	0.004	0.0441		0.33	0.010	0.004	0.011
rs10497870	A	0.61		0.33	0.008	0.004	0.071		0.30	-0.003	0.004	0.385
rs10498767	G	0.45		0.38	-0.01	0.005	0.0287		0.40	0.008	0.004	0.033

© 2020 Pang et al. *JAMA Cardiology.*

rs10498891	T	0.93		0.99	0.068	0.061	0.27		Low MAF*			
rs10499276	T	0.11		0.06	0.015	0.009	0.0963		0.05	-0.008	0.009	0.377
rs10499694	A	0.49		0.61	0.006	0.004	0.149		0.60	-0.005	0.004	0.214
rs10510419	G	0.15		0.003	0.05	0.044	0.263		Low MAF*			
rs10518269	C	0.17		0.001	-0.031	0.022	0.155		Low MAF*			
rs10744146	G	0.54		0.68	-0.013	0.005	0.00542		0.69	0.015	0.004	<5E-08
rs1075901	C	0.45		0.79	-0.011	0.005	0.0276		0.74	0.011	0.004	0.53
rs10761247	G	0.59		0.44	0.004	0.004	0.344		0.54	0.011	0.004	0.001
rs10761785	G	0.51		0.66	-0.011	0.004	0.0153		0.69	0.000	0.00393	0.001465
rs10783779	T	0.6		0.7	-0.001	0.005	0.812		0.66	0.002	0.004	0.593
rs10792006	T	0.45		0.71	0.006	0.005	0.183		0.66	-0.004	0.004	0.257
rs10797987	C	0.51		0.32	-0.003	0.005	0.557		0.38	0.010	0.004	0.008
rs10823893	A	0.41		0.08	0.001	0.008	0.9		0.04	-0.002	0.009	0.825
rs10840606	G	0.82		0.95	-0.023	0.01	0.0156		0.96	0.032	0.009	0.001
rs10850185	A	0.3		0.4	0.014	0.004	0.00112		0.36	-0.006	0.004	0.13
rs10850777	A	0.44		0.43	-0.004	0.004	0.368		0.43	-0.010	0.004	0.005
rs10858334	G	0.85		0.9	-0.011	0.007	0.111		0.88	-0.001	0.006	0.815
rs10871589	G	0.68		0.64	-0.009	0.004	0.0394		0.62	0.016	0.004	<5E-08
rs10886017	A	0.24		0.4	0.005	0.004	0.28		0.36	-0.006	0.004	0.125
rs10887584	A	0.45		0.27	0.017	0.005	0.000262		0.36	-0.002	0.004	0.627
rs10892873	C	0.36		0.18	0.009	0.006	0.0954		0.05	-0.006	0.008	0.358
rs10896012	C	0.78		0.85	0.009	0.006	0.133		0.82	0.002	0.005	0.716
rs10920678	A	0.43		0.34	0.01	0.004	0.0219		0.35	-0.021	0.004	<5E-08
rs10923724	C	0.56		0.6	-0.006	0.004	0.169		0.61	0.015	0.004	<5E-08
rs10929925	C	0.41		0.39	-0.016	0.004	0.00021		0.42	0.006	0.004	0.103
rs10930502	A	0.69		0.31	-0.003	0.005	0.585		0.29	-0.002	0.004	0.606
rs10937094	A	0.71		0.87	0.004	0.006	0.512		0.77	-0.002	0.004	0.693
rs10938397	G	0.57		0.7	-0.037	0.005	1.45E-15		0.7	0.028	0.004	<5E-08
rs10942267	A	0.69		0.82	0.008	0.005	0.134		0.85	-0.017	0.005	0.001

© 2020 Pang et al. *JAMA Cardiology.*

rs10950289	A	0.84		0.76	-0.005	0.005	0.382		0.76	-0.001	0.005	0.84
rs10954772	T	0.32		0.04	-0.015	0.011	0.192		Low MAF*			
rs10965780	G	0.41		0.15	-0.008	0.006	0.191		0.27	0.019	0.004	<5E-08
rs10971721	C	0.1		0.01	0.04	0.025	0.117		Low MAF*			
rs10984756	G	0.91		0.99	0.086	0.032	0.00761		Low MAF*			
rs10985968	C	0.5		0.52	0.003	0.004	0.531		0.57	-0.002	0.004	0.673
rs11001963	T	0.55		0.34	0.01	0.005	0.0389		0.35	-0.013	0.004	<5E-08
rs11047132	G	0.92		0.78	-0.002	0.005	0.656		0.71	0.007	0.004	0.078
rs1106761	A	0.37		0.16	0	0.006	0.98		0.14	-0.017	0.005	0.001
rs1106908	G	0.45		0.33	-0.016	0.004	0.000402		0.41	0.014	0.004	<5E-08
rs11074446	T	0.85		0.73	0.035	0.005	1.10E-13		0.72	-0.030	0.005	<5E-08
rs11075489	C	0.47		0.55	-0.009	0.004	0.0437		0.58	0.003	0.004	0.42
rs11105839	T	0.37		0.5	-0.019	0.004	2.24E-05		0.48	0.013	0.004	<5E-08
rs11115176	T	0.78		0.79	0.001	0.005	0.824		0.84	-0.001	0.005	0.888
rs11121210	C	0.34		0.17	-0.003	0.006	0.57		0.16	0.010	0.005	0.039
rs11126822	A	0.31		0.16	0.012	0.006	0.0359		0.2	-0.002	0.004	0.685
rs11138313	A	0.9		0.99	-0.009	0.053	0.865		Low MAF*			
rs11158434	A	0.2		0.42	0.003	0.004	0.485		0.55	-0.003	0.004	0.331
rs11170468	A	0.78		0.99	0.026	0.021	0.204		Low MAF*			
rs11181001	A	0.48		0.41	0.002	0.004	0.629		0.4	-0.008	0.004	0.029
rs11185111	G	0.29		0.47	-0.004	0.004	0.406		0.5	0.008	0.004	0.018
rs1119950	G	0.57		0.3	-0.015	0.005	0.00121		0.45	0.006	0.004	0.12
rs11218510	G	0.39		0.28	-0.006	0.005	0.233		0.26	0.008	0.004	0.049
rs112566467	T	0.21		0.19	0.012	0.005	0.0207		0.17	-0.008	0.005	0.12
rs113397893	G	0.92		0.99	-0.144	0.096	0.131		Low MAF*			
rs11525873	T	0.9		0.75	0.02	0.005	6.56E-05		0.64	-0.013	0.004	0.001
rs11577094	T	0.08		0.01	0.009	0.024	0.697		Low MAF*			
rs11608710	G	0.94		0.99	-0.329	0.141	0.0191		Low MAF*			
rs11611246	T	0.2		0.33	0.014	0.005	0.0016		0.3	-0.002	0.004	0.565

© 2020 Pang et al. *JAMA Cardiology.*

rs11629783	C	0.77		0.86	0.009	0.006	0.146		0.93	-0.026	0.007	<5E-08
rs11633626	C	0.63		0.68	-0.007	0.004	0.107		0.72	0.006	0.004	0.101
rs11636611	T	0.5		0.8	0.007	0.005	0.193		0.82	-0.003	0.005	0.482
rs11638950	A	0.59		0.71	0.008	0.005	0.0818		0.71	-0.006	0.004	0.137
rs11649864	A	0.09		0.01	0.037	0.031	0.229		Low MAF*			
rs11655587	C	0.36		0.17	-0.011	0.006	0.0537		0.17	0.014	0.005	0.005
rs11672660	C	0.19		0.19	-0.024	0.005	6.43E-06		0.23	0.025	0.004	<5E-08
rs1167821	T	0.55		0.95	-0.02	0.01	0.0408		Low MAF*			
rs11692326	T	0.23		0.11	0.022	0.007	0.00125		0.16	-0.016	0.005	0.001
rs11702843	A	0.25		0.03	0.008	0.012	0.52		0.03	-0.004	0.011	0.753
rs11739877	T	0.62		0.55	-0.003	0.004	0.415		0.6	-0.001	0.004	0.758
rs11757278	T	0.7		0.38	0.011	0.004	0.0105		0.49	-0.009	0.004	0.008
rs11761528	C	0.09		0.0001	-0.033	0.059	0.571		Low MAF*			
rs11781222	T	0.85		0.72	0.001	0.005	0.805		0.70	-0.008	0.004	0.038
rs11836108	A	0.3		0.19	0.01	0.005	0.0655		0.17	-0.011	0.005	0.027
rs11856579	G	0.26		0.03	-0.006	0.013	0.644		0.01	0.017	0.018	0.337
rs11866815	C	0.26		0.21	-0.011	0.005	0.0314		0.20	0.000	0.004	0.963
rs1187352	C	0.34		0.18	-0.013	0.005	0.0188		0.17	0.010	0.005	0.039
rs11874040	A	0.71		0.49	0.006	0.004	0.136		0.57	-0.008	0.004	0.025
rs11915371	C	0.8		0.93	0.009	0.008	0.245		0.97	0.006	0.01	0.567
rs11931941	A	0.22		0.39	0.011	0.004	0.015		0.36	0.0001	0.004	0.934
rs11971098	G	0.91		0.99	-0.022	0.032	0.496		Low MAF*			
rs1199334	A	0.19		0.03	0.027	0.012	0.0308		0.04	-0.024	0.009	0.01
rs11997238	G	0.91		0.83	-0.011	0.006	0.0532		0.84	0.012	0.005	0.012
rs12022461	G	0.17		0.21	-0.015	0.005	0.00347		0.15	0.013	0.005	0.008
rs12049202	T	0.19		0.36	-0.005	0.004	0.293		0.39	-0.005	0.004	0.212
rs12072739	G	0.78		0.92	0.009	0.007	0.241		0.97	0.013	0.01	0.205
rs12098284	T	0.12		0.13	-0.004	0.006	0.499		0.12	-0.004	0.006	0.468
rs12121950	T	0.34		0.76	0.009	0.005	0.0652		0.65	-0.013	0.004	0.001

© 2020 Pang et al. *JAMA Cardiology.*

rs12147845	T	0.11		0.0001	0.006	0.039	0.875		Low MAF*			
rs12151152	G	0.41		0.31	-0.027	0.005	9.49E-09		Low MAF*			
rs12193797	A	0.87		0.99	-0.017	0.022	0.431		Low MAF*			
rs12206094	C	0.29		0.2	-0.02	0.005	0.000148		0.17	0.015	0.005	0.002
rs12209887	A	0.46		0.29	0.019	0.005	4.50E-05		0.27	-0.004	0.004	0.359
rs12222235	C	0.33		0.16	-0.014	0.006	0.0182		0.09	0.002	0.006	0.725
rs12238336	C	0.38		0.43	-0.004	0.004	0.299		0.34	0.001	0.004	0.703
rs12282785	C	0.22		0.17	0.003	0.006	0.615		0.15	0.004	0.005	0.427
rs12286929	G	0.5		0.73	-0.01	0.005	0.027		0.80	0.015	0.005	0.001
rs12316047	G	0.24		0.01	-0.057	0.05	0.252		Low MAF*			
rs12321904	T	0.5		0.24	0	0.005	0.978		0.23	-0.005	0.004	0.221
rs12336441	A	0.62		0.99	-0.008	0.027	0.775		Low MAF*			
rs12364470	G	0.85		0.99	-0.049	0.027	0.0646		Low MAF*			
rs12422552	G	0.27		0.3	0.001	0.005	0.812		0.24	-0.006	0.004	0.152
rs12429545	A	0.12		0.25	0.034	0.005	1.97E-12		0.22	-0.031	0.004	<5E-08
rs12431682	C	0.34		0.46	-0.003	0.004	0.501		0.49	0.009	0.004	0.014
rs12438629	C	0.97		0.87	0.024	0.006	0.000127		0.85	-0.018	0.005	<5E-08
rs12439798	T	0.44		0.3	0.007	0.005	0.157		0.3	-0.009	0.004	0.023
rs12446632	G	0.14		0.0001	-0.123	0.071	0.0834		Low MAF*			
rs12448257	A	0.22		0.28	0.029	0.005	1.29E-09		0.3	-0.023	0.004	<5E-08
rs12449219	G	0.86		0.92	-0.022	0.008	0.00469		0.88	0.007	0.006	0.21
rs12449442	A	0.22		0.61	0.019	0.004	9.08E-06		0.7	-0.021	0.004	<5E-08
rs12454712	C	0.62		0.55	-0.016	0.004	0.000168		0.52	0.019	0.004	<5E-08
rs12462975	A	0.32		0.19	0.004	0.006	0.437		0.23	-0.009	0.004	0.037
rs12470698	A	0.57		0.8	0.01	0.005	0.0636		0.77	-0.011	0.004	0.016
rs12488237	C	0.94		0.94	0.003	0.008	0.737		0.93	0.012	0.007	0.1
rs12509234	C	0.72		0.63	-0.009	0.004	0.0377		0.94	0.0001	0.00371	0.009134
rs12522567	T	0.49		0.17	0.007	0.006	0.236		0.15	-0.007	0.005	0.165
rs12546331	T	0.51		0.6	0.005	0.004	0.267		0.63	-0.014	0.004	<5E-08

© 2020 Pang et al. *JAMA Cardiology.*

rs12551906	G	0.29		0.09	-0.001	0.007	0.838		0.1	0.010	0.006	0.097
rs12628051	T	0.64		0.6	0.018	0.004	3.23E-05		0.53	-0.024	0.004	<5E-08
rs12628891	C	0.32		0.46	-0.003	0.004	0.514		0.41	0.002	0.004	0.613
rs12635553	A	0.49		0.92	0.007	0.008	0.399		0.85	-0.003	0.005	0.563
rs1263627	T	0.76		0.59	-0.008	0.004	0.0599		0.68	0.006	0.004	0.114
rs12651833	C	0.81		0.57	0.002	0.004	0.658		0.42	-0.007	0.004	0.046
rs12680842	A	0.68		0.51	0.013	0.004	0.00312		0.5	-0.019	0.004	<5E-08
rs12681792	A	0.2		0.28	0.007	0.005	0.151		0.31	-0.001	0.004	0.759
rs1269175	A	0.51		0.86	0.011	0.006	0.0873		0.81	-0.009	0.005	0.047
rs12692596	T	0.36		0.26	-0.003	0.005	0.519		0.34	0.003	0.004	0.374
rs12705916	C	0.62		0.71	-0.005	0.005	0.277		0.69	0.007	0.004	0.086
rs12714199	C	0.61		0.79	-0.01	0.005	0.0423		0.67	0.004	0.004	0.334
rs12765914	T	0.08		0.25	0.016	0.005	0.00149		0.18	-0.016	0.005	0.001
rs1277733	T	0.77		0.76	0.014	0.005	0.00425		0.66	-0.017	0.004	<5E-08
rs1285245	G	0.39		0.34	-0.014	0.004	0.0015		0.33	0.018	0.004	<5E-08
rs12868881	A	0.41		0.41	0.013	0.004	0.00259		0.39	-0.003	0.004	0.352
rs12885454	C	0.36		0.56	-0.013	0.004	0.0019		0.52	0.001	0.004	0.848
rs12889085	G	0.58		0.81	-0.009	0.005	0.0993		0.83	0.007	0.005	0.149
rs12890931	G	0.63		0.36	-0.002	0.004	0.572		0.38	0.002	0.004	0.646
rs12899905	C	0.26		0.04	-0.017	0.011	0.115		0.03	0.027	0.011	0.01
rs12912380	C	0.11		0.05	-0.014	0.01	0.165		0.03	0.014	0.011	0.196
rs12914623	G	0.27		0.14	-0.002	0.006	0.767		0.14	0.012	0.006	0.035
rs12922346	C	0.26		0.16	0.014	0.006	0.0153		0.2	0.014	0.005	0.003
rs12939549	A	0.56		0.7	0.02	0.005	1.39E-05		0.65	-0.008	0.004	0.041
rs13002946	T	0.27		0.55	0	0.004	0.915		0.46	0.002	0.004	0.59
rs13021737	G	0.16		0.09	-0.082	0.007	4.38E-28		0.1	0.049	0.006	<5E-08
rs13033310	A	0.25		0.31	0.007	0.005	0.139		0.31	0.004	0.004	0.241
rs13062093	G	0.63		0.85	0.005	0.006	0.442		0.85	-0.003	0.005	0.573
rs13110266	G	0.4		0.51	0.001	0.004	0.771		0.45	0.0001	0.004	0.975

© 2020 Pang et al. *JAMA Cardiology.*

rs13155259	G	0.67		0.9	0.007	0.007	0.332		0.92	0.0001	0.007	0.962
rs13174863	G	0.85		0.95	-0.003	0.01	0.794		0.94	0.025	0.008	0.001
rs13191362	A	0.86		0.99	0.017	0.056	0.768		Low MAF*			
rs1320251	C	0.46		0.58	-0.012	0.004	0.00743		0.66	0.013	0.004	0.001
rs13209872	G	0.35		0.45	0	0.004	0.995		0.36	0.009	0.004	0.018
rs13264909	A	0.57		0.34	0	0.004	0.955		0.28	-0.001	0.004	0.89
rs13298062	A	0.81		0.99	0.031	0.02	0.12		0.94	-0.008	0.007	0.288
rs13299788	C	0.85		0.83	0.002	0.006	0.774		0.80	-0.015	0.004	<5E-08
rs13303252	T	0.18		0.4	0.004	0.004	0.316		0.36	0.003	0.004	0.403
rs13417156	C	0.42		0.34	-0.007	0.004	0.139		0.29	0.005	0.004	0.172
rs13432055	C	0.71		0.95	-0.009	0.01	0.352		0.95	0.017	0.009	0.053
rs1346841	G	0.41		0.55	-0.012	0.004	0.008		0.6	0.007	0.004	0.07
rs1355459	A	0.32		0.09	-0.002	0.008	0.783		0.04	-0.010	0.009	0.23
rs1356506	T	0.63		0.5	0.019	0.004	9.61E-06		0.61	-0.017	0.004	<5E-08
rs1363695	C	0.24		0.21	-0.006	0.005	0.241		0.16	0.0001	0.005	0.993
rs1383592	A	0.21		0.22	0.005	0.005	0.377		0.14	-0.004	0.005	0.426
rs1394879	C	0.4		0.12	0.006	0.006	0.385		0.05	-0.016	0.008	0.042
rs1409818	T	0.11		0.15	0.011	0.006	0.0735		0.13	-0.011	0.005	0.036
rs1411431	A	0.16		0.27	0.011	0.005	0.0211		0.35	-0.016	0.004	<5E-08
rs1417665	T	0.8		0.9	0.005	0.007	0.506		0.95	0.002	0.008	0.839
rs1426652	A	0.17		0.31	0.021	0.005	6.92E-06		0.32	-0.004	0.004	0.277
rs1431659	A	0.27		0.31	0.001	0.005	0.893		0.29	-0.009	0.004	0.026
rs1436344	C	0.57		0.36	0.004	0.004	0.337		0.35	-0.011	0.004	0.004
rs1437842	G	0.49		0.31	-0.002	0.005	0.6		0.33	0.011	0.004	0.003
rs1451077	G	0.42		0.41	-0.007	0.004	0.118		0.32	0.006	0.004	0.122
rs1451109	G	0.68		0.54	-0.002	0.004	0.641		0.47	0.002	0.004	0.592
rs1452075	T	0.73		0.71	0.007	0.005	0.119		0.8	0.001	0.004	0.289
rs1460676	C	0.82		0.62	-0.005	0.004	0.23		0.6	0.009	0.004	0.015
rs1470545	T	0.04		0.01	0.037	0.038	0.327		Low MAF*			

© 2020 Pang et al. *JAMA Cardiology.*

rs1471212	A	0.47		0.91	-0.003	0.007	0.652		0.96	-0.007	0.009	0.44
rs1477890	G	0.49		0.5	-0.004	0.004	0.318		0.47	0.007	0.004	0.044
rs1481012	A	0.89		0.71	0.012	0.005	0.0109		0.7	-0.012	0.004	0.002
rs1491905	T	0.53		0.74	0.011	0.005	0.0212		0.7	-0.011	0.004	0.005
rs1501673	A	0.14		0.49	0.023	0.004	4.18E-08		0.44	-0.020	0.004	<5E-08
rs1506662	A	0.53		0.62	0.005	0.004	0.26		0.66	-0.002	0.004	0.658
rs1514177	C	0.43		0.77	0.032	0.005	1.01E-10		0.83	-0.017	0.005	<5E-08
rs1522569	T	0.82		0.99	0.037	0.032	0.256		Low MAF*			
rs1523768	G	0.67		0.83	-0.011	0.006	0.0556		0.82	-0.002	0.005	0.675
rs1536053	C	0.31		0.05	0.016	0.01	0.106		0.02	0.018	0.011	0.108
rs1541777	G	0.54		0.85	-0.004	0.006	0.485		0.88	0.008	0.005	0.145
rs1546924	T	0.5		0.34	0.002	0.005	0.695		0.38	-0.007	0.004	0.079
rs1554194	C	0.5		0.53	0.023	0.004	6.27E-08		0.5	0.009	0.004	0.019
rs1561554	G	0.61		0.92	-0.006	0.007	0.416		0.94	0.004	0.008	0.593
rs1580099	C	0.58		0.68	-0.013	0.005	0.00305		0.74	0.014	0.004	0.001
rs1582931	G	0.47		0.6	-0.022	0.004	4.66E-07		0.53	0.017	0.004	0.511
rs159032	T	0.23		0.13	0.004	0.006	0.542		0.18	0.005	0.005	0.303
rs1601817	C	0.31		0.16	-0.003	0.006	0.563		0.13	0.011	0.005	0.034
rs1634350	A	0.44		0.7	0.016	0.005	0.000756		0.64	-0.008	0.004	0.039
rs1635853	G	0.41		0.33	-0.01	0.004	0.0227		0.3	-0.003	0.004	0.502
rs1658820	T	0.24		0.38	0.014	0.004	0.00226		0.29	-0.013	0.004	0.001
rs16834431	T	0.21		0.01	-0.019	0.03	0.518		Low MAF*			
rs16851483	T	0.07		0.24	0.025	0.005	4.90E-07		0.29	-0.016	0.004	<5E-08
rs16882001	G	0.95		0.96	-0.021	0.01	0.0317		0.98	0.006	0.011	0.606
rs16906845	G	0.06		0.12	-0.018	0.006	0.0043		0.15	0.016	0.005	0.002
rs16907751	C	0.09		0.19	-0.016	0.005	0.00238		0.14	0.028	0.005	<5E-08
rs16940823	C	0.17		0.09	0.001	0.007	0.845		0.09	-0.001	0.006	0.836
rs16978350	G	0.28		0.11	0.008	0.007	0.221		0.12	0.010	0.005	0.066
rs16989232	A	0.39		0.52	-0.001	0.004	0.823		0.42	-0.002	0.004	0.509

© 2020 Pang et al. *JAMA Cardiology.*

rs17001561	A	0.15		0.02	0.003	0.014	0.82		0.01	0.008	0.018	0.644
rs17014375	G	0.87		0.87	-0.006	0.006	0.295		0.88	0.008	0.006	0.158
rs17019336	T	0.23		0.35	-0.01	0.004	0.0215		0.27	0.011	0.004	0.006
rs1707322	G	0.32		0.3	-0.014	0.005	0.0019		0.32	0.004	0.004	0.354
rs17105272	T	0.32		0.47	0.011	0.004	0.0109		0.43	-0.011	0.004	0.003
rs17193211	C	0.07		0.07	-0.018	0.008	0.0326		0.07	0.013	0.008	0.092
rs17201143	C	0.31		0.48	-0.004	0.004	0.411		0.44	0.013	0.004	<5E-08
rs1721447	G	0.51		0.22	-0.001	0.005	0.848		0.32	0.002	0.004	0.553
rs17276464	T	0.41		0.02	-0.005	0.016	0.751	Low MAF*				
rs1730859	G	0.65		0.74	-0.004	0.005	0.451		0.72	0.006	0.004	0.158
rs17405819	T	0.68		0.55	0.023	0.004	6.40E-08		0.39	-0.021	0.004	<5E-08
rs17425707	C	0.9		0.99	-0.03	0.043	0.489		Low MAF*			
rs17448885	C	0.65		0.57	0.012	0.004	0.00672		0.62	-0.006	0.004	0.096
rs17522122	T	0.47		0.36	0.012	0.004	0.00555		0.35	-0.010	0.004	0.014
rs17535749	A	0.11		0.001	0.075	0.068	0.27		Low MAF*			
rs17551974	C	0.19		0.19	-0.013	0.005	0.0141		0.19	0.008	0.005	0.069
rs17574378	T	0.73		0.85	0.009	0.006	0.138		0.84	-0.007	0.005	0.122
rs17609108	C	0.85		0.85	-0.011	0.006	0.0552		0.81	-0.011	0.005	0.019
rs17636031	C	0.72		0.98	-0.018	0.019	0.35		Low MAF*			
rs17681451	G	0.08		0.01	0.032	0.029	0.262		Low MAF*			
rs17720922	T	0.19		0.08	0.001	0.008	0.857		0.11	0.002	0.006	0.773
rs17757975	T	0.85		0.99	0.006	0.028	0.836		Low MAF*			
rs17806224	G	0.18		0.15	-0.012	0.006	0.0479		0.14	0.011	0.005	0.033
rs1819844	A	0.17		0.28	0.019	0.005	8.24E-05		0.19	-0.013	0.005	0.006
rs1852006	G	0.38		0.44	-0.028	0.004	6.50E-11		0.43	0.014	0.004	<5E-08
rs1871329	G	0.76		0.71	-0.008	0.005	0.106		0.68	-0.006	0.004	0.136
rs1876359	T	0.37		0.35	0	0.004	0.951		0.42	-0.007	0.004	0.044
rs1877875	C	0.43		0.3	-0.009	0.005	0.0534		0.34	0.002	0.004	0.64
rs1884389	C	0.44		0.8	0.015	0.005	0.00413		0.67	0.010	0.004	0.008

© 2020 Pang et al. *JAMA Cardiology.*

rs1884429	T	0.23		0.07	0.019	0.008	0.0258		0.06	-0.011	0.008	0.163
rs1884897	G	0.37		0.14	-0.021	0.006	0.000737		0.09	0.014	0.006	0.024
rs1891215	C	0.54		0.66	0.004	0.004	0.426		0.53	0.003	0.004	0.491
rs1899689	T	0.39		0.5	0.01	0.004	0.0141		0.41	-0.010	0.004	0.005
rs1911746	T	0.21		0.26	-0.006	0.005	0.246		0.34	-0.008	0.004	0.045
rs1916801	A	0.62		0.43	0.003	0.004	0.491		0.31	-0.011	0.004	0.009
rs1927790	C	0.61		0.66	-0.016	0.004	0.000294		0.58	0.010	0.004	0.005
rs1928295	T	0.57		0.63	0.012	0.004	0.00771		0.56	-0.015	0.004	<5E-08
rs1941697	A	0.45		0.8	0.002	0.005	0.765		0.76	-0.013	0.004	0.001
rs194809	A	0.19		0.27	0.004	0.005	0.406		0.19	-0.012	0.005	0.009
rs1951455	C	0.28		0.11	-0.01	0.007	0.167		0.12	0.004	0.007	0.56
rs1954494	T	0.56		0.48	0.007	0.004	0.129		0.49	-0.003	0.004	0.343
rs1967772	G	0.27		0.06	-0.002	0.009	0.837		0.03	0.031	0.01	0.002
rs1982350	G	0.64		0.62	0.004	0.005	0.354		0.67	0.007	0.004	0.065
rs1982441	T	0.13		0.0001	0.069	0.042	0.0993		Low MAF*			
rs1987960	C	0.05		0.0001	-0.304	0.155	0.0499		Low MAF*			
rs1990573	G	0.31		0.34	0.005	0.005	0.255		0.46	0.005	0.004	0.203
rs1996120	G	0.59		0.65	-0.03	0.004	2.38E-11		0.65	0.007	0.004	0.066
rs2012502	A	0.38		0.18	0.013	0.006	0.0171		0.19	-0.013	0.005	0.006
rs2033529	G	0.73		0.81	-0.006	0.005	0.266		0.7	0.011	0.004	0.006
rs2044469	G	0.66		0.77	-0.006	0.005	0.218		0.73	0.004	0.004	0.281
rs2051559	C	0.86		0.85	-0.003	0.006	0.554		0.8	-0.001	0.004	0.831
rs2053682	A	0.68		0.83	0.008	0.006	0.132		0.91	-0.011	0.006	0.07
rs2058527	G	0.27		0.28	-0.014	0.005	0.00316		0.33	0.003	0.004	0.49
rs2063177	A	0.35		0.11	-0.003	0.007	0.64		0.12	-0.001	0.005	0.873
rs2065418	T	0.65		0.63	0.016	0.004	0.000425		0.64	-0.012	0.004	0.001
rs2070929	C	0.29		0.51	0.007	0.004	0.0876		0.55	-0.002	0.004	0.607
rs2072518	A	0.57		0.46	0.004	0.004	0.398		0.51	0.0001	0.004	0.911
rs2072597	G	0.31		0.39	-0.01	0.004	0.016		0.29	0.004	0.004	0.344

© 2020 Pang et al. *JAMA Cardiology.*

rs2074881	C	0.19		0.28	-0.014	0.005	0.00408		0.29	0.007	0.005	0.104
rs2081493	C	0.3		0.19	-0.003	0.005	0.531		0.26	0.003	0.004	0.438
rs2105808	C	0.67		0.97	-0.029	0.012	0.0147		0.95	0.006	0.008	0.434
rs2107118	A	0.5		0.48	0.012	0.004	0.00443		0.57	-0.011	0.004	0.004
rs2112347	T	0.63		0.43	0.02	0.004	2.73E-06		0.44	-0.024	0.004	<5E-08
rs2134858	C	0.51		0.42	-0.005	0.004	0.26		0.47	0.010	0.004	0.004
rs2155645	C	0.26		0.38	-0.01	0.004	0.028		0.35	0.007	0.004	0.054
rs215614	G	0.38		0.39	0.002	0.004	0.573		0.34	0.011	0.004	0.002
rs2170382	T	0.11		0.0001	0.053	0.029	0.0674		Low MAF*			
rs217669	C	0.72		0.57	-0.005	0.004	0.236		0.61	0.001	0.004	0.775
rs2178899	A	0.88		0.98	-0.012	0.014	0.402		0.99	0.009	0.017	0.587
rs2183824	T	0.31		0.3	0.002	0.005	0.71		0.27	0.001	0.004	0.843
rs2187449	A	0.77		0.93	0.001	0.008	0.862		0.93	-0.008	0.007	0.266
rs2192158	A	0.55		0.6	-0.003	0.004	0.549		0.63	-0.004	0.004	0.325
rs2196618	G	0.26		0.38	-0.007	0.004	0.0972		0.29	0.011	0.004	0.017
rs2206277	T	0.16		0.23	0.037	0.005	1.60E-13		0.02	-0.029	0.015	0.873
rs2228213	G	0.34		0.23	-0.017	0.005	0.000684		0.27	0.017	0.004	<5E-08
rs223058	G	0.33		0.04	-0.009	0.011	0.424		0.02	0.002	0.014	0.916
rs2237403	C	0.64		0.55	-0.001	0.004	0.755		0.52	0.009	0.004	0.014
rs2241743	G	0.43		0.36	-0.003	0.004	0.532		0.35	0.005	0.004	0.188
rs2246012	C	0.85		0.65	-0.003	0.004	0.549		0.65	0.006	0.004	0.099
rs2299383	T	0.4		0.45	0.016	0.004	0.000154		0.38	-0.010	0.004	0.009
rs2307022	A	0.33		0.14	0.013	0.006	0.0385		0.12	-0.015	0.006	0.008
rs2322622	C	0.35		0.14	0.007	0.006	0.228		0.08	0.008	0.005	0.09
rs2342892	T	0.49		0.58	0.021	0.004	1.04E-06		0.63	-0.015	0.004	<5E-08
rs2357760	A	0.67		0.76	0.014	0.005	0.00632		0.69	-0.012	0.004	0.002
rs2391540	T	0.67		0.79	0.008	0.005	0.148		0.83	0.006	0.005	0.22
rs2396625	T	0.58		0.63	-0.01	0.004	0.028		0.63	-0.002	0.004	0.646
rs2400414	C	0.35		0.25	-0.018	0.005	0.000217		0.22	0.013	0.004	0.002

© 2020 Pang et al. *JAMA Cardiology.*

rs2425857	A	0.43		0.38	0.014	0.004	0.00138		0.36	-0.005	0.004	0.174
rs2434467	T	0.34		0.27	0.014	0.005	0.0047		0.35	-0.013	0.004	<5E-08
rs2439823	G	0.45		0.74	-0.004	0.005	0.46		0.71	0.003	0.004	0.438
rs2447832	T	0.44		0.33	0.007	0.005	0.12		0.37	-0.004	0.004	0.276
rs2450448	A	0.69		0.93	0.003	0.008	0.668		0.92	0.0001	0.007	0.954
rs2455793	A	0.42		0.63	-0.005	0.004	0.291		0.61	-0.005	0.004	0.199
rs2470397	C	0.81		0.99	-0.002	0.022	0.937		Low MAF*			
rs249293	C	0.7		0.43	0.003	0.004	0.525		0.5	-0.003	0.004	0.42
rs249612	T	0.71		0.46	0.009	0.004	0.0426		0.42	-0.005	0.004	0.151
rs2577947	C	0.82		0.88	-0.006	0.006	0.357		0.89	0.013	0.006	0.027
rs2596902	G	0.35		0.39	-0.014	0.004	0.00146		0.41	0.005	0.004	0.15
rs2601777	G	0.39		0.44	-0.019	0.004	9.37E-06		0.47	0.009	0.004	0.011
rs2605603	G	0.48		0.87	-0.009	0.006	0.158		0.85	0.007	0.005	0.178
rs2619976	T	0.4		0.27	0.004	0.005	0.412		0.23	-0.004	0.004	0.341
rs2622274	G	0.46		0.43	-0.016	0.004	0.000269		0.41	0.012	0.004	0.001
rs264962	C	0.54		0.35	-0.01	0.004	0.0288		0.28	-0.001	0.004	0.795
rs2670854	A	0.72		0.83	0.012	0.006	0.0388		0.86	-0.003	0.005	0.5
rs2682406	T	0.59		0.64	-0.013	0.004	0.00265		0.61	0.010	0.004	0.006
rs2693686	G	0.61		0.74	0	0.005	0.999		0.8	-0.005	0.004	0.275
rs2715423	G	0.28		0.06	0	0.009	0.991		0.04	-0.007	0.009	0.45
rs273505	C	0.58		0.66	-0.004	0.004	0.431		0.7	0.012	0.004	0.004
rs2777768	A	0.72		0.96	0.004	0.01	0.677		0.98	-0.019	0.012	0.12
rs2803316	G	0.46		0.39	-0.003	0.004	0.455		0.41	0.007	0.004	0.057
rs2820295	A	0.33		0.12	0.006	0.007	0.394		0.1	-0.021	0.006	<5E-08
rs2832283	A	0.23		0.05	0.003	0.01	0.772		0.04	-0.012	0.01	0.07
rs28350	A	0.17		0.08	0.017	0.008	0.0343		0.08	-0.015	0.007	0.022
rs2837398	C	0.61		0.47	-0.011	0.004	0.00753		0.47	-0.001	0.004	0.849
rs284227	C	0.73		0.95	-0.021	0.009	0.0274		0.95	0.005	0.008	0.548
rs2861089	A	0.38		0.1	0.007	0.007	0.324		0.1	-0.006	0.006	0.287

© 2020 Pang et al. *JAMA Cardiology.*

rs2861685	T	0.59		0.41	0.006	0.004	0.166		0.37	-0.010	0.004	0.006
rs2862996	G	0.7		0.84	-0.003	0.006	0.6		0.86	0.003	0.005	0.597
rs2870710	C	0.81		0.74	0.02	0.005	2.99E-05		0.68	-0.013	0.004	0.001
rs288230	T	0.84		0.84	0.006	0.006	0.304		0.85	-0.003	0.005	0.597
rs2899663	G	0.49		0.57	0.002	0.004	0.712		0.43	0.004	0.004	0.264
rs2907948	G	0.24		0.05	-0.006	0.01	0.571		0.04	0.009	0.009	0.336
rs2933451	C	0.67		0.75	-0.005	0.005	0.32		0.74	0.008	0.004	0.062
rs2954021	G	0.51		0.43	0.005	0.004	0.238		0.45	0.002	0.004	0.564
rs2968487	T	0.28		0.41	0.01	0.004	0.024		0.43	-0.006	0.004	0.085
rs29938	C	0.66		0.76	-0.027	0.005	7.52E-08		0.79	0.014	0.004	0.001
rs3101336	C	0.38		0.08	-0.022	0.008	0.00379		0.07	0.025	0.007	<5E-08
rs3134358	T	0.62		0.64	0.006	0.004	0.202		0.56	-0.003	0.004	0.366
rs325220	G	0.3		0.06	-0.007	0.009	0.476		0.11	0.014	0.006	0.016
rs326845	G	0.38		0.48	-0.001	0.004	0.872		0.5	-0.003	0.004	0.38
rs329124	A	0.59		0.6	0.018	0.004	3.21E-05		0.63	-0.019	0.004	<5E-08
rs340025	C	0.41		0.27	-0.01	0.005	0.0466		0.17	0.004	0.005	0.38
rs34277166	A	0.44		0.11	0.011	0.007	0.117		0.08	-0.015	0.006	0.017
rs34720381	T	0.09		0.08	0.006	0.008	0.433		0.07	-0.008	0.007	0.265
rs34801745	C	0.36		0.84	0.002	0.006	0.776		0.84	0.001	0.005	0.836
rs349088	C	0.48		0.34	-0.001	0.005	0.767		0.44	0.004	0.004	0.257
rs354508	C	0.16		0.21	-0.008	0.005	0.113		0.2	0.003	0.004	0.488
rs355777	C	0.4		0.43	0.007	0.004	0.11		0.53	-0.009	0.004	0.008
rs355914	C	0.63		0.52	0.001	0.004	0.868		0.47	-0.007	0.004	0.05
rs35809007	G	0.36		0.48	-0.001	0.004	0.877		0.55	-0.002	0.004	0.566
rs35851183	G	0.64		0.57	-0.005	0.004	0.203		0.52	0.013	0.004	<5E-08
rs36061954	T	0.4		0.33	0.019	0.005	2.45E-05		0.3	0.0001	0.004	0.989
rs3738476	C	0.88		0.63	-0.004	0.005	0.38		0.91	0.004	0.006	0.519
rs3739514	A	0.34		0.55	0.009	0.004	0.0353		0.55	-0.004	0.004	0.315
rs3744017	A	0.19		0.14	0.003	0.006	0.646		0.15	-0.001	0.005	0.864

© 2020 Pang et al. *JAMA Cardiology.*

rs3746038	C	0.22		0.41	-0.007	0.004	0.0997		0.5	0.008	0.004	0.043
rs3750944	G	0.56		0.5	-0.009	0.004	0.0287		0.63	0.004	0.004	0.291
rs3752904	C	0.51		0.46	-0.003	0.004	0.464		0.48	0.0001	0.004	0.995
rs3766160	G	0.77		0.53	-0.011	0.004	0.00805		0.52	0.003	0.004	0.438
rs3770799	G	0.64		0.78	-0.008	0.005	0.111		0.81	0.008	0.004	0.068
rs3774573	T	0.74		0.84	0.012	0.006	0.0413		0.81	-0.011	0.005	0.014
rs3806114	G	0.69		0.8	-0.004	0.006	0.493		0.76	0.003	0.004	0.45
rs3808477	C	0.27		0.3	-0.019	0.005	4.32E-05		0.27	0.015	0.004	<5E-08
rs3811125	C	0.28		0.24	-0.008	0.005	0.127		0.27	0.010	0.004	0.024
rs3822683	A	0.79		0.58	0	0.004	0.984		0.76	0.002	0.004	0.254
rs3825061	T	0.39		0.17	0.009	0.006	0.127		0.12	-0.010	0.006	0.07
rs3914628	T	0.86		0.9	0.003	0.007	0.696		0.1	-0.007	0.005	0.218
rs3922980	G	0.5		0.84	0.001	0.006	0.875		Low MAF*			
rs3923783	C	0.18		0.13	-0.005	0.006	0.405		0.11	0.008	0.006	0.172
rs3930349	C	0.21		0.29	-0.011	0.005	0.0235		0.36	0.006	0.004	0.113
rs3935190	G	0.54		0.69	-0.016	0.005	0.000684		0.6	0.015	0.004	<5E-08
rs39654	G	0.45		0.4	-0.009	0.004	0.0363		0.41	0.009	0.004	0.015
rs4097319	T	0.56		0.42	0.001	0.004	0.798		0.41	-0.002	0.004	0.652
rs4123853	T	0.37		0.25	0.003	0.005	0.55		0.18	-0.011	0.005	0.018
rs41290587	T	0.01		0.0001	0.085	0.192	0.659		Low MAF*			
rs4239020	T	0.68		0.93	-0.005	0.008	0.588		0.95	0.010	0.008	0.251
rs4242244	T	0.58		0.37	0.014	0.004	0.00218		0.28	-0.007	0.004	0.092
rs4256980	G	0.67		0.62	-0.022	0.004	2.91E-07		0.62	0.019	0.004	<5E-08
rs4270551	A	0.13		0.003	0.086	0.05	0.0864		Low MAF*			
rs4273371	C	0.51		0.82	-0.01	0.005	0.0573		0.79	0.001	0.004	0.787
rs427943	C	0.43		0.49	-0.014	0.004	0.00113		0.56	0.010	0.004	0.008
rs4290163	T	0.4		0.58	0.015	0.004	0.000442		0.46	-0.017	0.004	<5E-08
rs4307239	G	0.46		0.3	-0.003	0.005	0.544		0.19	0.005	0.005	0.231
rs4372836	T	0.33		0.27	0.013	0.005	0.0085		0.32	-0.007	0.004	0.065

© 2020 Pang et al. *JAMA Cardiology.*

rs4382152	T	0.62		0.2	0.01	0.005	0.068		0.19	-0.010	0.004	0.011
rs4414033	A	0.6		0.69	0.006	0.005	0.19		0.69	-0.014	0.004	<5E-08
rs4421883	C	0.49		0.67	0.002	0.005	0.733		0.74	0.002	0.004	0.683
rs4581940	T	0.59		0.75	0.02	0.005	3.22E-05		0.75	-0.002	0.004	0.632
rs459552	T	0.77		0.91	-0.001	0.007	0.895		0.9	0.0001	0.006	0.969
rs4609871	T	0.54		0.4	0.014	0.004	0.00112		0.31	-0.012	0.004	0.001
rs4671328	T	0.45		0.38	0.02	0.004	4.96E-06		0.41	-0.020	0.004	<5E-08
rs4700608	C	0.48		0.15	-0.015	0.006	0.0147		0.15	0.004	0.005	0.41
rs4703019	A	0.39		0.4	-0.001	0.004	0.738		0.35	-0.007	0.004	0.044
rs4721089	T	0.78		0.89	0.008	0.007	0.231		0.88	0.005	0.006	0.399
rs4721823	G	0.14		0.44	-0.012	0.004	0.00404		0.51	0.004	0.004	0.241
rs4722398	T	0.15		0.02	0.012	0.014	0.418		0.06	-0.006	0.007	0.408
rs4739570	A	0.62		0.59	0.01	0.004	0.021		0.54	-0.013	0.004	<5E-08
rs4740619	T	0.54		0.24	0.015	0.005	0.00199		0.23	-0.012	0.004	0.005
rs4746339	T	0.26		0.03	0.01	0.012	0.417		0.04	-0.026	0.009	0.004
rs4794977	C	0.57		0.76	-0.002	0.005	0.709		0.74	-0.002	0.004	0.682
rs4795195	A	0.29		0.41	0	0.004	0.983		0.37	-0.004	0.004	0.259
rs4802570	C	0.4		0.75	-0.001	0.005	0.88		0.75	-0.009	0.004	0.029
rs4814512	A	0.81		0.8	0.012	0.005	0.0278		0.71	-0.006	0.004	0.117
rs4818226	G	0.31		0.09	0.011	0.008	0.144		0.08	-0.004	0.007	0.522
rs4834272	C	0.69		0.46	-0.01	0.004	0.0236		0.53	0.014	0.004	<5E-08
rs4841504	C	0.5		0.04	-0.014	0.011	0.221		0.03	-0.014	0.011	0.223
rs4851057	T	0.13		0.03	0.011	0.013	0.389		0.02	0.002	0.014	0.877
rs4857968	G	0.29		0.04	-0.018	0.012	0.118		0.03	0.008	0.011	0.473
rs487152	A	0.5		0.33	0.012	0.005	0.0072		0.29	-0.020	0.004	<5E-08
rs4880341	C	0.57		0.94	-0.01	0.008	0.234		0.92	0.0001	0.01	0.39
rs4895231	C	0.47		0.2	-0.005	0.006	0.398		0.13	0.0001	0.00555	0.4097
rs4906263	G	0.66		0.58	-0.028	0.004	4.90E-11		0.54	0.011	0.004	0.003
rs4973618	G	0.66		0.5	-0.004	0.004	0.404		0.49	0.009	0.004	0.014

© 2020 Pang et al. *JAMA Cardiology.*

rs4985155	A	0.67		0.55	0.01	0.004	0.0151		0.55	-0.016	0.004	<5E-08
rs5017416	T	0.05		0.01	0.028	0.029	0.324		Low MAF*			
rs507856	T	0.56		0.44	0.01	0.004	0.0235		0.36	-0.008	0.004	0.041
rs5215	T	0.63		0.62	0.013	0.004	0.00301		0.63	-0.018	0.004	<5E-08
rs539100	A	0.22		0.02	0.013	0.015	0.379		0.04	-0.001	0.01	0.893
rs543874	G	0.78		0.82	-0.056	0.005	3.01E-25		0.78	0.047	0.004	<5E-08
rs544722	T	0.45		0.76	0.004	0.005	0.395		0.79	-0.002	0.004	0.604
rs55676934	G	0.67		0.81	-0.005	0.005	0.349		0.81	0.004	0.005	0.416
rs559267	G	0.67		0.58	-0.015	0.004	0.000416		0.56	0.018	0.004	<5E-08
rs55966114	T	0.19		0.16	0.01	0.006	0.0938		0.14	-0.013	0.005	0.014
rs561136	C	0.87		0.92	-0.021	0.008	0.00826		0.81	0.009	0.004	0.041
rs56356382	T	0.81		0.99	0.033	0.037	0.372		Low MAF*			
rs57800857	A	0.63		0.73	-0.014	0.005	0.0036		0.76	-0.011	0.004	0.009
rs58139454	G	0.22		0.19	-0.007	0.005	0.18		0.22	0.010	0.004	0.026
rs592483	C	0.41		0.21	-0.02	0.005	0.000221		0.19	0.004	0.005	0.386
rs59302296	A	0.1		0.002	0.06	0.054	0.271		Low MAF*			
rs594821	C	0.09		0.06	-0.031	0.009	0.000535		0.12	0.010	0.006	0.075
rs6019483	T	0.17		0.08	-0.015	0.008	0.0632		0.12	0.004	0.006	0.44
rs6023633	G	0.78		0.99	0.002	0.04	0.956		Low MAF*			
rs6092179	C	0.2		0.15	-0.027	0.006	5.23E-06		0.12	0.020	0.005	<5E-08
rs61051952	A	0.75		0.62	0	0.004	0.939		0.67	-0.003	0.004	0.383
rs6130360	A	0.85		0.75	0.014	0.005	0.00417		0.8	-0.017	0.005	<5E-08
rs6142067	T	0.6		0.75	0.014	0.005	0.00411		0.65	-0.009	0.004	0.019
rs615568	G	0.52		0.9	0.012	0.007	0.0765		0.9	0.005	0.006	0.385
rs61740466	G	0.77		0.51	-0.02	0.004	3.26E-06		0.65	0.007	0.004	0.079
rs61813324	T	0.13		0.003	0.021	0.051	0.68		Low MAF*			
rs61828641	A	0.11		0.26	0.023	0.005	1.44E-06		0.21	-0.021	0.004	<5E-08
rs61903695	G	0.75		0.9	-0.008	0.007	0.247		0.89	0.013	0.006	0.017
rs62118504	G	0.61		0.98	-0.005	0.015	0.723		0.99	-0.010	0.028	0.731

© 2020 Pang et al. *JAMA Cardiology.*

rs6265	C	0.18		0.49	-0.036	0.004	1.75E-17		0.41	0.038	0.004	<5E-08
rs630602	C	0.61		0.33	0.002	0.005	0.628		0.38	0.001	0.004	0.804
rs6419734	T	0.14		0.01	-0.008	0.023	0.745		Low MAF*			
rs6435622	C	0.32		0.33	0.015	0.005	0.00138		0.48	-0.013	0.004	<5E-08
rs6443750	C	0.2		0.01	-0.039	0.048	0.416		Low MAF*			
rs6451675	G	0.32		0.18	-0.019	0.006	0.000672		0.19	0.004	0.004	0.312
rs6463489	T	0.1		0.08	0.004	0.008	0.619		0.04	-0.003	0.009	0.74
rs6476617	G	0.38		0.2	-0.01	0.005	0.0642		0.26	0.011	0.004	0.006
rs6477694	C	0.36		0.43	-0.014	0.004	0.00148		0.39	0.016	0.004	<5E-08
rs6493498	T	0.45		0.48	0.007	0.004	0.0947		0.59	0.002	0.004	0.586
rs6500208	A	0.2		0.42	0.002	0.004	0.689		0.39	-0.010	0.004	0.005
rs650198	C	0.73		0.76	0.008	0.005	0.107		0.72	0.003	0.004	0.464
rs6511027	C	0.17		0.02	-0.027	0.015	0.0708		0.01	-0.004	0.016	0.813
rs6512302	C	0.74		0.72	0.005	0.005	0.304		0.73	-0.003	0.004	0.438
rs651548	A	0.36		0.38	0.008	0.004	0.0651		0.41	0.006	0.004	0.121
rs6539064	C	0.75		0.66	0.005	0.005	0.3		0.64	-0.009	0.004	0.02
rs6540498	T	0.62		0.55	-0.002	0.004	0.61		0.42	0.003	0.004	0.473
rs6545714	G	0.61		0.64	0.008	0.004	0.0603		0.7	0.009	0.004	0.018
rs6556301	G	0.63		0.55	-0.011	0.004	0.00766		0.57	0.007	0.004	0.066
rs6561766	A	0.13		0.11	0	0.007	0.949		0.12	-0.012	0.005	0.028
rs6567160	C	0.75		0.8	-0.06	0.005	3.45E-30		0.78	0.051	0.004	<5E-08
rs6574100	A	0.59		0.9	0.011	0.007	0.123		0.9	-0.006	0.006	0.314
rs6577584	G	0.66		0.82	-0.009	0.005	0.11		0.82	0.014	0.005	0.002
rs6591407	C	0.2		0.1	-0.006	0.007	0.421		0.15	0.008	0.005	0.09
rs6602411	T	0.18		0.02	0.014	0.014	0.334		0.01	0.008	0.016	0.634
rs6604015	C	0.91		0.97	-0.016	0.011	0.155		0.98	0.019	0.014	0.163
rs6606686	G	0.7		0.99	-0.031	0.025	0.204		0.98	0.009	0.013	0.691
rs6607337	C	0.3		0.25	-0.01	0.005	0.0521		0.75	0.004	0.004	0.359
rs6661316	T	0.59		0.44	0.013	0.004	0.00322		0.42	-0.013	0.004	<5E-08

© 2020 Pang et al. *JAMA Cardiology.*

rs6690398	A	0.55		0.51	0.012	0.004	0.00364		0.44	-0.009	0.004	0.017
rs6696828	C	0.3		0.28	0.008	0.005	0.079		0.25	-0.004	0.004	0.341
rs6710871	A	0.14		0.1	0.025	0.007	0.000328		0.14	-0.011	0.005	0.034
rs6720868	T	0.31		0.23	0	0.005	0.925		0.23	-0.007	0.004	0.1
rs6733834	C	0.81		0.71	0.019	0.005	5.98E-05		0.78	-0.018	0.004	<5E-08
rs6781254	T	0.3		0.34	0.006	0.004	0.201		0.32	-0.006	0.004	0.15
rs6804181	A	0.82		0.84	0.02	0.006	0.000617		0.76	-0.016	0.004	<5E-08
rs6804842	G	0.43		0.37	-0.008	0.005	0.0782		0.38	0.013	0.004	0.001
rs6804915	A	0.29		0.25	0.02	0.005	4.50E-05		0.23	-0.011	0.004	0.012
rs6809307	T	0.26		0.36	0.011	0.004	0.0124		0.39	-0.013	0.004	<5E-08
rs6818414	C	0.52		0.69	-0.003	0.005	0.469		0.72	0.011	0.004	0.007
rs6832762	G	0.51		0.08	0.003	0.008	0.689		0.05	0.002	0.008	0.82
rs6850421	A	0.46		0.54	0.003	0.004	0.442		0.44	0.0001	0.004	0.99
rs6857	C	0.17		0.1	-0.004	0.007	0.616		0.17	0.014	0.005	0.004
rs6864049	G	0.49		0.3	-0.005	0.005	0.25		0.31	0.015	0.004	<5E-08
rs6890310	G	0.29		0.09	-0.01	0.008	0.211		0.08	0.0001	0.006	0.993
rs6905288	G	0.57		0.73	-0.012	0.005	0.0144		0.79	-0.005	0.005	0.244
rs6916553	G	0.24		0.02	-0.015	0.016	0.341		0.03	0.028	0.011	0.013
rs6922607	G	0.81		0.92	0.001	0.007	0.886		0.89	0.0001	0.006	0.979
rs6931385	A	0.48		0.4	-0.001	0.004	0.904		0.48	-0.008	0.004	0.028
rs6932930	G	0.79		0.65	-0.006	0.004	0.171		0.25	0.004	0.004	0.33
rs6934973	G	0.7		0.58	-0.006	0.004	0.138		0.68	0.003	0.004	0.356
rs6954694	G	0.84		0.63	-0.007	0.005	0.108		Low MAF*			
rs6962280	G	0.44		0.19	-0.009	0.005	0.0996		0.19	0.013	0.005	0.011
rs6968554	G	0.61		0.58	-0.005	0.004	0.248		0.66	0.004	0.004	0.236
rs6970595	C	0.58		0.8	0.002	0.005	0.638		0.77	0.005	0.004	0.279
rs698147	A	0.45		0.45	0.005	0.004	0.244		0.51	-0.002	0.004	0.551
rs7024334	T	0.23		0.57	0.009	0.004	0.0362		0.58	-0.014	0.004	<5E-08
rs7031064	A	0.53		0.59	0.004	0.004	0.3		0.42	0.0001	0.004	0.927

© 2020 Pang et al. *JAMA Cardiology.*

rs7037266	C	0.36		0.69	-0.003	0.005	0.483		0.63	0.008	0.004	0.035
rs704061	C	0.55		0.62	-0.006	0.004	0.159		0.95	0.005	0.009	0.059
rs7070670	C	0.32		0.18	-0.001	0.006	0.878		0.13	0.0001	0.006	0.97
rs7084454	A	0.31		0.01	0.018	0.018	0.322		Low MAF*			
rs709400	A	0.62		0.93	-0.006	0.008	0.486		0.87	-0.010	0.005	0.054
rs7123876	C	0.75		0.84	-0.007	0.006	0.24		0.76	0.015	0.004	<5E-08
rs7124681	A	0.42		0.31	0.021	0.005	4.48E-06		0.31	-0.022	0.004	<5E-08
rs7133378	A	0.33		0.13	0.011	0.006	0.0826		0.16	-0.008	0.005	0.117
rs7134628	A	0.1		0.01	-0.008	0.032	0.814		Low MAF*			
rs713763	A	0.42		0.19	-0.001	0.005	0.91		0.19	-0.004	0.005	0.459
rs7138803	A	0.39		0.28	0.023	0.005	1.63E-06		0.35	-0.010	0.004	0.007
rs7141307	C	0.74		0.86	-0.016	0.006	0.00996		0.79	0.008	0.005	0.083
rs7144011	T	0.23		0.002	-0.028	0.044	0.525		Low MAF*			
rs715	C	0.7		0.82	-0.011	0.005	0.0504		0.84	0.014	0.005	0.005
rs7181610	A	0.86		0.99	-0.028	0.062	0.656		0.84	-0.002	0.005	0.607
rs7186893	G	0.26		0.1	0	0.007	0.997		0.08	0.014	0.006	0.03
rs7189122	C	0.83		0.66	-0.005	0.004	0.258		0.66	0.009	0.004	0.012
rs719802	T	0.4		0.48	0.001	0.004	0.82		0.56	-0.007	0.004	0.052
rs7206608	G	0.68		0.94	-0.015	0.009	0.0776		0.94	0.001	0.008	0.88
rs7217226	G	0.66		0.74	-0.016	0.005	0.000727		0.77	0.020	0.004	<5E-08
rs7219230	T	0.34		0.74	0.015	0.005	0.00134		0.85	-0.005	0.005	0.56
rs7238896	G	0.86		0.99	-0.036	0.044	0.418		Low MAF*			
rs7239114	A	0.54		0.12	0.017	0.007	0.0104		0.11	-0.014	0.006	0.01
rs7243357	T	0.83		0.8	0.029	0.005	4.75E-08		0.77	-0.015	0.005	0.005
rs7259325	G	0.48		0.7	-0.004	0.005	0.402		0.7	-0.003	0.004	0.515
rs72673947	G	0.89		0.92	-0.006	0.008	0.437		0.9	0.004	0.006	0.486
rs730180	A	0.79		0.46	0.006	0.004	0.163		0.49	-0.005	0.004	0.145
rs7318817	C	0.63		0.83	-0.018	0.006	0.00129		0.86	0.012	0.005	0.014
rs73213501	A	0.83		0.83	-0.002	0.006	0.659		0.9	-0.004	0.006	0.541

© 2020 Pang et al. *JAMA Cardiology.*

rs73249175	G	0.88		0.99	0.006	0.066	0.93		Low MAF*			
rs7357754	G	0.5		0.72	0.001	0.005	0.864		0.65	0.005	0.004	0.175
rs740157	A	0.45		0.37	0.01	0.004	0.0185		0.37	-0.010	0.004	0.005
rs7498044	G	0.21		0.01	-0.016	0.025	0.513		Low MAF*			
rs7498665	G	0.62		0.88	-0.017	0.006	0.00793		0.86	0.030	0.005	<5E-08
rs7523668	G	0.44		0.72	0	0.005	0.981		0.65	0.005	0.004	0.17
rs7534091	G	0.72		0.66	-0.017	0.004	0.000104		0.51	0.015	0.004	<5E-08
rs7537581	A	0.53		0.53	0.013	0.004	0.00324		0.48	0.0001	0.00411	0.03179
rs7561278	T	0.77		0.67	0.016	0.005	0.000322		0.56	-0.016	0.004	<5E-08
rs7570446	A	0.54		0.59	0	0.004	0.962		0.43	0.000	0.004	0.988
rs7575118	T	0.16		0.02	0.002	0.021	0.916		0.02	-0.024	0.012	0.051
rs7588437	G	0.37		0.25	-0.016	0.005	0.000979		0.22	0.018	0.004	<5E-08
rs7589023	T	0.84		0.96	0.017	0.011	0.106		0.96	0.003	0.009	0.753
rs7599312	G	0.27		0.03	-0.018	0.012	0.134		0.04	0.015	0.01	0.111
rs7601895	C	0.69		0.94	0.014	0.009	0.11		0.91	0.003	0.006	0.675
rs761423	T	0.54		0.73	0.006	0.005	0.221		0.71	-0.011	0.004	0.005
rs7616009	G	0.16		0.42	0.004	0.004	0.336		0.5	0.012	0.004	0.001
rs7621025	C	0.26		0.13	-0.005	0.006	0.427		0.18	0.015	0.005	0.002
rs7631156	A	0.31		0.13	0.025	0.006	7.41E-05		0.08	-0.021	0.006	0.001
rs765875	C	0.47		0.55	-0.009	0.004	0.04		0.55	0.0001	0.004	0.918
rs7678054	G	0.48		0.7	-0.007	0.005	0.124		0.79	0.013	0.004	0.003
rs76942203	A	0.06		0.1	0.019	0.007	0.00628		0.1	-0.019	0.006	0.002
rs7713317	G	0.72		0.67	-0.019	0.004	3.21E-05		0.78	0.021	0.004	<5E-08
rs7714712	C	0.37		0.19	-0.021	0.005	0.000116		0.14	0.012	0.005	0.023
rs7734385	G	0.44		0.23	0.005	0.005	0.303		0.26	0.005	0.004	0.199
rs7740107	T	0.74		0.97	-0.035	0.011	0.00171		0.98	0.030	0.012	0.013
rs77432547	G	0.72		0.95	0.011	0.009	0.232		0.95	0.020	0.008	0.013
rs7769594	T	0.16		0.14	-0.005	0.006	0.41		0.07	-0.004	0.007	0.541
rs7777084	A	0.46		0.41	0.003	0.004	0.417		0.37	-0.012	0.004	0.002

© 2020 Pang et al. *JAMA Cardiology.*

rs7784465	C	0.86		0.99	-0.006	0.016	0.724		0.99	0.009	0.018	0.624
rs779206	G	0.72		0.67	0.006	0.004	0.16		0.64	0.004	0.004	0.249
rs7802342	G	0.7		0.78	-0.013	0.005	0.0106		0.74	0.010	0.004	0.016
rs7826312	C	0.43		0.83	0	0.006	0.969		0.84	-0.003	0.005	0.514
rs786420	T	0.77		0.36	0.006	0.004	0.179		0.42	0.001	0.004	0.842
rs7871866	C	0.16		0.19	0.014	0.006	0.0102		0.22	-0.016	0.005	<5E-08
rs7893571	T	0.67		0.89	-0.005	0.007	0.485		0.9	-0.017	0.006	0.003
rs7899106	G	0.95		0.99	-0.061	0.035	0.0791		Low MAF*			
rs7903146	C	0.28		0.04	-0.012	0.011	0.291		0.04	-0.057	0.009	<5E-08
rs79113395	G	0.27		0.15	-0.008	0.006	0.221		Low MAF*			
rs7944782	G	0.5		0.45	-0.013	0.004	0.00231		0.55	0.005	0.004	0.15
rs7947143	G	0.15		0.13	0.003	0.006	0.583		0.09	0.001	0.006	0.85
rs7961979	A	0.13		0.002	-0.042	0.051	0.411		Low MAF*			
rs7968390	A	0.68		0.57	0.008	0.004	0.076		0.41	-0.010	0.004	0.004
rs7975187	G	0.77		0.69	0.001	0.005	0.877		0.62	0.013	0.004	0.001
rs7976757	C	0.18		0.28	0.001	0.005	0.816		0.23	0.007	0.004	0.091
rs8016859	C	0.04		0.001	0.063	0.084	0.453		Low MAF*			
rs8024806	T	0.94		0.98	-0.006	0.015	0.667		0.99	0.007	0.017	0.696
rs8033510	T	0.36		0.39	0.008	0.004	0.0703		0.4	-0.009	0.004	0.017
rs8038464	T	0.4		0.5	-0.013	0.004	0.00318		0.47	0.008	0.004	0.033
rs8075273	C	0.29		0.07	-0.007	0.008	0.392		0.05	-0.004	0.008	0.633
rs8081039	T	0.06		0.005	0.034	0.033	0.298		0.03	0.016	0.011	0.149
rs8092503	G	0.77		0.57	-0.007	0.004	0.0858		0.7	0.018	0.004	<5E-08
rs8122855	A	0.34		0.62	0.01	0.004	0.0185		0.62	0.001	0.004	0.756
rs8134638	C	0.63		0.75	0.003	0.005	0.59		0.77	0.005	0.005	0.286
rs816367	G	0.65		0.67	-0.008	0.005	0.0766		0.64	0.008	0.004	0.023
rs8181823	C	0.23		0.08	-0.011	0.008	0.136		0.09	0.005	0.006	0.423
rs845084	A	0.27		0.43	0.022	0.004	2.20E-07		0.35	-0.013	0.004	0.001
rs857601	T	0.71		0.96	0.001	0.01	0.906		0.97	0.030	0.01	0.003

© 2020 Pang et al. *JAMA Cardiology.*

rs867560	G	0.57		0.15	-0.018	0.006	0.0027		0.1	0.014	0.006	0.019
rs872281	C	0.18		0.32	-0.004	0.005	0.324		0.25	0.006	0.004	0.143
rs889398	C	0.42		0.12	-0.004	0.007	0.5		0.14	0.006	0.005	0.258
rs891387	T	0.5		0.08	0.014	0.008	0.0662		0.06	-0.018	0.007	0.015
rs9077	G	0.27		0.06	-0.014	0.009	0.117		0.05	-0.002	0.008	0.771
rs930295	A	0.15		0.19	0.008	0.005	0.142		0.17	-0.011	0.005	0.029
rs9317002	A	0.51		0.32	0.008	0.005	0.0718		0.3	-0.010	0.004	0.014
rs9320823	C	0.41		0.42	0.005	0.004	0.216		0.37	0.007	0.004	0.048
rs934515	A	0.12		0.2	0.014	0.005	0.0109		0.18	0.005	0.005	0.265
rs9376609	A	0.38		0.28	0.006	0.005	0.224		0.21	0.007	0.004	0.091
rs942066	G	0.63		0.8	-0.007	0.005	0.202		0.82	0.018	0.005	<5E-08
rs944340	T	0.42		0.64	0.003	0.004	0.47		0.6	0.0001	0.004	0.992
rs945211	C	0.63		0.32	0.007	0.005	0.12		0.3	-0.011	0.004	0.004
rs946824	T	0.14		0.11	0.008	0.007	0.236		0.09	-0.007	0.006	0.257
rs947088	T	0.74		0.99	0.029	0.029	0.319		Low MAF*			
rs9479509	A	0.29		0.62	0.008	0.004	0.0583		0.5	-0.015	0.004	<5E-08
rs9514131	G	0.13		0.1	-0.009	0.007	0.219		0.1	0.006	0.006	0.31
rs9527455	C	0.23		0.08	-0.019	0.008	0.0156		Low MAF*			
rs9540493	A	0.46		0.75	0.01	0.005	0.0615		0.67	-0.016	0.005	0.001
rs9571687	C	0.34		0.38	-0.016	0.004	0.000326		0.37	0.003	0.004	0.424
rs9595908	T	0.64		0.83	0.009	0.006	0.0989		0.84	-0.014	0.005	0.006
rs9603697	T	0.32		0.48	0.01	0.004	0.0176		0.49	-0.007	0.004	0.061
rs972540	G	0.72		0.8	-0.005	0.005	0.327		0.83	0.013	0.005	0.008
rs9808302	G	0.53		0.52	0.004	0.004	0.315		0.47	-0.007	0.004	0.038
rs9816029	C	0.34		0.08	0.015	0.008	0.047		0.09	-0.005	0.006	0.403
rs9816226	T	0.18		0.05	-0.061	0.01	1.27E-09		0.02	0.040	0.015	0.008
rs9818122	C	0.79		0.99	-0.013	0.031	0.673		Low MAF*			
rs9826775	A	0.85		0.86	-0.007	0.006	0.24		0.87	-0.005	0.005	0.338
rs9839267	T	0.89		0.85	0.028	0.006	2.60E-06		0.83	-0.011	0.005	0.018

© 2020 Pang et al. *JAMA Cardiology.*

rs9839653	G	0.83		0.99	-0.071	0.046	0.122		Low MAF*			
rs985060	G	0.27		0.43	-0.002	0.004	0.601		0.39	0.001	0.004	0.846
rs9862795	T	0.52		0.85	0.005	0.006	0.388		0.84	0.015	0.005	0.002
rs987071	C	0.77		0.99	0.012	0.026	0.648		Low MAF*			
rs9929792	A	0.5		0.86	0.013	0.006	0.0269		0.91	0.013	0.006	0.042
rs9937053	A	0.44		0.17	0.072	0.006	2.09E-38		0.23	-0.069	0.004	<5E-08
rs9965170	G	0.43		0.14	-0.009	0.006	0.152		0.15	0.012	0.005	0.016

Abbreviation: EAF, effect allele frequency.
*Low minor allele frequency (MAF, <1%) in the BBJ population.

© 2020 Pang et al. *JAMA Cardiology.*

eTable 5. Associations of potential confounders with BMI GRS

Confounder	OR (95% CI) per 1-SD higher genetically-instrumented BMI
**Current smoking**	1.03 (0.94, 1.13)
**Weekly drinking**	1.09 (0.98, 1.21)
**Physical activity**	0.93 (0.82, 1.04)
**Education**	0.99 (0.89, 1.10)
**Income**	1.02 (0.95, 1.09)

The model was adjusted for age at baseline, age squared, sex, 10 regions, and 12 PCs, education, smoking, and alcohol.
Potential confounders were dichotomised: current smoking (yes vs no), weekly drinking (yes vs no), total PA (≥30 vs <30 MET-h/day), education (≥9 vs <9 years), and household income (≥10,000 vs <10,000 RMB/year).
The analyses of smoking and alcohol were conducted in men due to the small number of women who smoked (3%) or drank (2%).

© 2020 Pang et al. *JAMA Cardiology.*

eTable 6. Baseline characteristics of study participants by BMI in all CKB participants

		BMI categories (kg/m^2)				
**Variable***	**<20**	**20 to <22.5**	**22.5 to <25**	**25 to <27.5**	**≥27.5**	**All**
	(n=65,986)	(n=131,198)	(n=144,703)	(n=85,427)	(n=82,578)	**(n=512,715)**
Age (SD), year	52.4 (13.0)	50.7 (10.9)	51.0 (10.3)	51.5 (10.2)	51.6 (10.9)	**51.5 (10.7)**
Female, %	55.8	57.0	59.5	59.5	64.1	**59.2**
**Socioeconomic and lifestyle factors**						
Urban resident, %	31.5	37.2	45.4	51.1	55.7	**44.1**
≥9 years of education, %	20	21.1	21.8	21.5	20.4	**20.9**
Household income ≥35 000 RMB/year, %	14.8	16.7	18.6	19.7	20.1	**17.9**
Ever regular smoking, %						
Male	76	71.4	65.7	62.8	61.4	**67.7**
Female	4.2	3	2.7	2.4	2.6	**2.8**
Weekly drinking %						
Male	31.5	34.1	33.8	32.7	32.4	**33.5**
Female	2.1	2.1	2.1	2.1	1.9	**2.1**
Total physical activity (SD), MET h/day	21.4 (13.9)	21.8 (14.3)	21.3 (14.0)	20.6 (13.6)	19.8 (13.0)	**21.1 (13.9)**
**Blood pressure, blood sugar and anthropometry**						
SBP (SD), mmHg	123.0 (20.9)	127.2 (20.4)	131.2 (20.6)	134.9 (20.8)	139.4 (21.3)	**131.1 (21.3)**
RPG (SD), mmol/L	5.9 (2.1)	5.9 (2.1)	6.0 (2.3)	6.2 (2.5)	6.4 (2.7)	**6.1 (2.3)**
BMI (SD), kg/m^2	18.7 (1.1)	21.3 (0.7)	23.7 (0.7)	25.9 (0.6)	29.0 (2.0)	**23.7 (3.4)**
Waist circumference (SD), cm	68.3 (5.2)	74.5 (5.3)	80.6 (5.8)	86.0 (5.9)	93.0 (7.4)	**80.3 (9.8)**
Hip circumference (SD), cm	83.6 (4.3)	87.4 (4.2)	90.9 (4.4)	94.2 (4.5)	99.0 (5.9)	**90.9 (6.9)**
Waist-to-hip ratio (SD)	0.82 (0.06)	0.85 (0.06)	0.89 (0.06)	0.91 (0.06)	0.94 (0.07)	**0.90 (0.07)**
**Prior disease history, %**						
Diabetes	3.4	4.3	5.8	7.2	9.1	**5.9**
CHD	1.9	2.2	2.9	3.5	4.3	**5.9**
Stroke or TIA	1.0	1.4	1.8	2.0	2.3	**1.7**
Hypertension	4.6	7.3	11.2	15.5	21.5	**11.6**
Family history of diabetes	3.7	4.2	5.0	5.4	6.0	**4.9**
Family history of cancer	13.0	13.5	14.2	14.4	14.6	**13.9**

* Results were adjusted for age, region, and sex (where appropriate). Values are means unless otherwise stated.
Abbreviations: MET=metabolic equivalent of task; SBP=systolic blood pressure; RPG=random plasma glucose; BMI=body mass index; HBsAg=hepatitis B surface antigen;

© 2020 Pang et al. *JAMA Cardiology.*

CHD=coronary heart disease; TIA=transient ischaemic attack.

© 2020 Pang et al. *JAMA Cardiology.*

eTable 7. Associations of BMI with proteomics and of proteomics with CVD

Protein	Observational				MR-IPD	MR-Egger		Weighted median		Observational: proteins and CVD		
	SD difference (95% CI)[1]	Uncorrected *p*-value	FDR-adjusted *p*-value		SD difference (95% CI)[2]	SD difference (95% CI)[2]	*p* for intercept	SD difference (95% CI)[2]		HR (95% CI)[3]	Uncorrected *p*-value	FDR-adjusted *p*-value
**Chemokines**												
CCL3	**0.11 (0.03,0.19)**	8.97E-03	2.88E-02		0.33 (0.20,0.47)	0.21 (-0.11,0.53)	0.06	0.24 (0.08,0.39)		1.10 (0.90,1.33)	0.349	0.777
CCL4	0.05 (-0.04,0.13)	2.91E-01	5.15E-01		0.27 (0.14,0.41)	0.17 (-0.10,0.45)	0.07	0.19 (0.04,0.35)		0.96 (0.79,1.16)	0.679	0.902
CCL17	-0.03 (-0.11,0.04)	3.94E-01	5.96E-01		-0.10 (-0.25,0.04)	-0.07 (-0.28,0.15)	0.48	-0.07 (-0.24,0.10)		0.84 (0.69,1.03)	0.099	0.547
CCL19	**0.18 (0.10,0.27)**	1.85E-05	2.01E-04		0.12 (-0.00,0.25)	0.08 (-0.12,0.28)	0.34	0.09 (-0.06,0.23)		1.02 (0.85,1.21)	0.869	0.978
CCL20	**0.12 (0.03,0.20)**	8.67E-03	2.88E-02		0.06 (-0.06,0.18)	0.04 (-0.14,0.22)	0.62	0.04 (-0.10,0.19)		**1.19 (1.01,1.41)**	0.041	0.372
CCL23	-0.06 (-0.15,0.02)	1.53E-01	3.29E-01		0.01 (-0.12,0.14)	0.01 (-0.18,0.20)	0.91	0.01 (-0.15,0.16)		0.99 (0.82,1.19)	0.903	0.984
MCP1	**0.12 (0.04,0.20)**	4.78E-03	1.96E-02		0.37 (0.23,0.52)	0.24 (-0.12,0.59)	0.08	0.27 (0.10,0.43)		1.12 (0.93,1.36)	0.236	0.733
MCP2	-0.05 (-0.14,0.03)	2.20E-01	4.22E-01		0.18 (0.06,0.30)	0.11 (-0.10,0.33)	0.12	0.13 (-0.01,0.27)		1.03 (0.85,1.27)	0.738	0.921
MCP3	**0.11 (0.02,0.19)**	1.15E-02	3.46E-02		0.09 (-0.02,0.20)	0.05 (-0.11,0.22)	0.44	0.06 (-0.07,0.19)		**1.28 (1.09,1.50)**	0.002	0.068
MCP4	0.03 (-0.04,0.11)	3.97E-01	5.96E-01		0.10 (-0.03,0.23)	0.06 (-0.14,0.26)	0.46	0.07 (-0.08,0.22)		1.13 (0.92,1.39)	0.258	0.748
CXCL1	-0.01 (-0.09,0.07)	8.68E-01	9.77E-01		-0.21 (-0.36,-0.06)	-0.13 (-0.40,0.13)	0.13	-0.15 (-0.32,0.02)		0.97 (0.79,1.20)	0.804	0.952
CXCL5	-0.00 (-0.08,0.08)	9.65E-01	9.91E-01		-0.07 (-0.18,0.04)	-0.04 (-0.21,0.12)	0.55	-0.05 (-0.18,0.08)		0.92 (0.75,1.14)	0.457	0.893
CXCL9	0.02 (-0.06,0.11)	6.09E-01	8.18E-01		-0.09 (-0.20,0.03)	-0.05 (-0.23,0.12)	0.48	-0.06 (-0.20,0.08)		1.13 (0.93,1.38)	0.219	0.733
CXCL10	**0.14 (0.06,0.23)**	7.35E-04	4.72E-03		0.22 (0.09,0.35)	0.14 (-0.10,0.38)	0.06	0.16 (0.01,0.30)		1.13 (0.93,1.37)	0.219	0.733
CXCL11	0.01 (-0.08,0.10)	8.32E-01	9.48E-01		0.12 (0.04,0.20)	0.08 (-0.07,0.22)	0.12	0.09 (-0.01,0.18)		0.89 (0.72,1.08)	0.231	0.733
CXCL12	-0.09 (-0.18,-0.00)	4.60E-02	1.25E-01		-0.25 (-0.37,-0.13)	-0.16 (-0.42,0.11)	0.08	-0.18 (-0.32,-0.03)		0.92 (0.75,1.12)	0.396	0.848
CXCL13	0.01 (-0.08,0.10)	8.19E-01	9.45E-01		-0.48 (-0.68,-0.29)	-0.31 (-0.78,0.17)	0.07	-0.35 (-0.57,-0.12)		**1.23 (1.01,1.50)**	0.037	0.365
CX3CL1	-0.04 (-0.12,0.04)	3.21E-01	5.45E-01		-0.29 (-0.40,-0.17)	-0.18 (-0.47,0.10)	0.08	-0.20 (-0.34,-0.07)		1.06 (0.87,1.29)	0.566	0.893
**Inflammatory cytokines**												
Interleukins												
IL1-alpha	0.02 (-0.07,0.11)	7.17E-01	9.05E-01		0.06 (0.26,-0.15)	0.03 (0.32,-0.25)	0.77	0.04 (0.28,-0.20)		1.12 (0.92,1.37)	0.995	0.997
IL2	-0.00 (-0.09,0.09)	9.36E-01	9.91E-01		-0.33 (-0.48,-0.18)	-0.21 (-0.54,0.12)	0.06	-0.23 (-0.41,-0.06)		0.63 (0.00, 1.01)	0.996	0.997
IL4	-0.00 (-0.09,0.08)	9.27E-01	9.91E-01		-0.15 (-0.23,-0.06)	-0.09 (-0.26,0.07)	0.06	-0.11 (-0.20,-0.01)		0.93 (0.82,1.06)	0.306	0.761
IL5	0.07 (-0.02,0.16)	1.37E-01	3.01E-01		0.25 (0.05,0.44)	0.16 (-0.17,0.49)	0.20	0.18 (-0.06,0.41)		1.04 (0.86,1.24)	0.706	0.904
IL6	**0.21 (0.12,0.29)**	2.12E-06	3.18E-05		0.35 (0.22,0.49)	0.22 (-0.11,0.56)	0.07	0.25 (0.09,0.41)		**1.21 (1.02,1.48)**	0.040	0.785
IL7	0.00 (-0.09,0.09)	9.61E-01	9.91E-01		-0.01 (-0.01,0.00)	-0.00 (-0.01,0.01)	0.36	-0.00 (-0.01,0.00)		1.08 (0.90,1.29)	0.415	0.868
IL8	0.02 (-0.07,0.10)	6.71E-01	8.75E-01		0.11 (-0.69,0.91)	0.07 (-1.07,1.21)	0.88	0.08 (-0.87,1.02)		1.06 (0.87,1.28)	0.584	0.893
IL10	0.07 (-0.02,0.16)	1.24E-01	2.86E-01		0.05 (-0.06,0.16)	0.03 (-0.13,0.19)	0.62	0.03 (-0.09,0.16)		1.07 (0.87,1.31)	0.542	0.893

© 2020 Pang et al. *JAMA Cardiology.*

Protein	Observational				MR-IPD	MR-Egger		Weighted median		Observational: proteins and CVD		
	SD difference (95% CI)[1]	Uncorrected	FDR-adjusted		SD difference (95% CI)[2]	SD difference (95% CI)[2]	*p* for intercept	SD difference (95% CI)[2]		HR (95% CI)[3]	Uncorrected	FDR-adjusted
		p-value	*p*-value								*p*-value	*p*-value
IL12	**0.13 (0.05,0.21)**	1.32E-03	6.27E-03		-0.22 (-0.35,-0.09)	-0.14 (-0.38,0.11)	0.07	-0.16 (-0.31,-0.01)		0.91 (0.75,1.10)	0.333	0.768
IL12-RB1	0.03 (-0.06,0.12)	4.94E-01	6.94E-01		-0.03 (-0.11,0.04)	-0.02 (-0.13,0.09)	0.62	-0.02 (-0.11,0.06)		1.03 (0.86,1.23)	0.757	0.921
IL13	-0.05 (-0.14,0.04)	2.53E-01	4.65E-01		-0.08 (-0.21,0.05)	-0.05 (-0.24,0.13)	0.53	-0.06 (-0.20,0.09)		0.98 (0.81,1.20)	0.863	0.978
IL18	**0.13 (0.05,0.21)**	1.96E-03	8.40E-03		0.28 (0.15,0.40)	0.17 (-0.10,0.45)	0.06	0.20 (0.05,0.35)		**1.25 (1.02,1.54)**	0.034	0.365
IL33	-0.06 (-0.15,0.02)	1.59E-01	3.33E-01		0.21 (0.07,0.34)	0.13 (-0.11,0.37)	0.10	0.15 (-0.01,0.30)		0.36 (0.07,1.94)	0.235	0.733
Interferon												
IFN-beta	-0.00 (-0.09,0.09)	9.93E-01	9.93E-01		0.03 (0.06,0.00)	0.02 (0.07,-0.03)	0.25	0.02 (0.06,-0.01)		0.85 (0.68,1.08)	0.184	0.733
IFN-gamma	-0.03 (-0.12,0.06)	5.77E-01	7.87E-01		-0.11 (-0.24,0.01)	-0.07 (-0.26,0.12)	0.36	-0.08 (-0.22,0.06)		0.92 (0.63,1.34)	0.665	0.902
TNF(R) superfamily												
TNF	-0.01 (-0.10,0.07)	7.48E-01	9.09E-01		-0.21 (-0.28,-0.14)	-0.14 (-0.34,0.07)	0.06	-0.15 (-0.23,-0.07)		0.75 (0.43,1.32)	0.321	0.761
TNFSF14	**0.27 (0.18,0.35)**	4.78E-10	1.43E-08		0.13 (-0.01,0.27)	0.08 (-0.14,0.30)	0.36	0.09 (-0.07,0.25)		1.04 (0.87,1.26)	0.646	0.902
TRAIL	**0.19 (0.10,0.28)**	1.80E-05	2.01E-04		0.61 (0.43,0.79)	0.39 (-0.17,0.95)	0.06	0.44 (0.23,0.64)		0.90 (0.74,1.10)	0.300	0.761
TWEAK	**-0.14 (-0.23,-0.06)**	1.26E-03	6.27E-03		-0.06 (-0.19,0.07)	-0.04 (-0.23,0.15)	0.62	-0.04 (-0.20,0.11)		**0.76 (0.64,0.91)**	0.002	0.068
TNFRSF4	0.04 (-0.05,0.12)	3.69E-01	5.72E-01		0.03 (-0.09,0.14)	0.02 (-0.15,0.19)	0.82	0.02 (-0.12,0.16)		1.05 (0.87,1.28)	0.611	0.893
TNFRSF9	0.05 (-0.03,0.13)	2.14E-01	4.19E-01		-0.04 (-0.17,0.09)	-0.02 (-0.21,0.17)	0.77	-0.03 (-0.18,0.13)		1.05 (0.87,1.27)	0.625	0.893
TNFRSF12A	**0.11 (0.03,0.18)**	6.57E-03	2.37E-02		0.13 (0.01,0.25)	0.08 (-0.11,0.28)	0.29	0.09 (-0.05,0.23)		1.20 (0.99,1.46)	0.065	0.456
TNFRSF21	0.00 (-0.08,0.09)	9.56E-01	9.91E-01		-0.13 (-0.21,-0.05)	-0.08 (-0.23,0.06)	0.07	-0.09 (-0.18,-0.00)		0.95 (0.78,1.15)	0.609	0.893
CD27	-0.04 (-0.12,0.04)	3.15E-01	5.45E-01		-0.28 (-0.41,-0.15)	-0.18 (-0.46,0.11)	0.07	-0.20 (-0.35,-0.05)		1.06 (0.87,1.29)	0.588	0.893
CD40	0.08 (-0.01,0.16)	7.94E-02	1.88E-01		0.07 (-0.06,0.20)	0.05 (-0.15,0.24)	0.59	0.05 (-0.10,0.21)		0.91 (0.75,1.10)	0.319	0.761
CD40L	0.00 (-0.08,0.09)	9.12E-01	9.91E-01		0.04 (0.09,-0.01)	0.03 (0.10,-0.05)	0.46	0.03 (0.09,-0.03)		0.94 (0.77,1.14)	0.530	0.893
CD70	**0.14 (0.06,0.22)**	9.61E-04	5.41E-03		0.11 (-0.02,0.24)	0.07 (-0.13,0.27)	0.42	0.08 (-0.07,0.23)		1.12 (0.92,1.36)	0.267	0.750
FasLG	**-0.11 (-0.19,-0.03)**	6.86E-03	2.37E-02		-0.11 (-0.24,0.01)	-0.07 (-0.27,0.13)	0.38	-0.08 (-0.23,0.07)		**0.77 (0.62,0.95)**	0.016	0.283
**Growth factors (receptors)**												
ANG1	-0.01 (-0.09,0.07)	8.07E-01	9.45E-01		-0.16 (-0.30,-0.01)	-0.10 (-0.33,0.14)	0.28	-0.11 (-0.28,0.06)		0.94 (0.76,1.15)	0.539	0.893
ANGPT2	0.01 (-0.07,0.10)	7.40E-01	9.09E-01		0.16 (-0.03,0.36)	0.10 (-0.20,0.41)	0.42	0.12 (-0.12,0.35)		0.96 (0.78,1.17)	0.681	0.902
CSF1	**0.17 (0.09,0.25)**	7.74E-05	6.33E-04		-0.18 (-0.31,-0.04)	-0.11 (-0.34,0.12)	0.16	-0.13 (-0.28,0.03)		1.12 (0.92,1.35)	0.255	0.748
EGF	0.05 (-0.03,0.13)	2.36E-01	4.43E-01		-0.34 (-0.53,-0.15)	-0.22 (-0.59,0.16)	0.06	-0.24 (-0.46,-0.03)		0.91 (0.74,1.11)	0.354	0.777
FGF2	0.07 (-0.02,0.16)	1.35E-01	3.01E-01		-0.14 (-0.29,0.02)	-0.09 (-0.33,0.15)	0.38	-0.10 (-0.28,0.08)		1.08 (0.89,1.30)	0.425	0.869
HGF	**0.31 (0.24,0.39)**	2.22E-15	1.99E-13		0.06 (-0.07,0.19)	0.04 (-0.15,0.23)	0.62	0.04 (-0.11,0.19)		**1.22 (1.02,1.44)**	0.045	0.863
LAP-TGF-beta1	0.03 (-0.05,0.11)	4.86E-01	6.94E-01		-0.15 (-0.22,-0.08)	-0.09 (-0.25,0.06)	0.09	-0.11 (-0.19,-0.03)		0.84 (0.67,1.05)	0.132	0.625
PDGF_subunit_b	-0.00 (-0.08,0.08)	9.51E-01	9.91E-01		-0.94 (-1.46,-0.43)	-0.60 (-1.62,0.42)	0.06	-0.67 (-1.26,-0.08)		0.99 (0.81,1.22)	0.948	0.997

© 2020 Pang et al. *JAMA Cardiology.*

Protein	Observational			MR-IPD	MR-Egger		Weighted median	Observational: proteins and CVD		
	SD difference (95% CI) [1]	Uncorrected *p*-value	FDR-adjusted *p*-value	SD difference (95% CI) [2]	SD difference (95% CI) [2]	*p* for intercept	SD difference (95% CI) [2]	HR (95% CI) [3]	Uncorrected *p*-value	FDR-adjusted *p*-value
PGF	**0.15 (0.07,0.23)**	2.38E-04	1.79E-03	-0.19 (-0.32,-0.06)	-0.12 (-0.36,0.11)	0.12	-0.14 (-0.29,0.01)	1.06 (0.88,1.28)	0.531	0.893
PTN	0.07 (0.01,0.13)	2.53E-02	7.36E-02	0.12 (-0.05,0.29)	0.08 (-0.18,0.33)	0.48	0.09 (-0.11,0.29)	1.08 (0.81,1.45)	0.595	0.893
VEGFA	**0.20 (0.12,0.27)**	1.13E-06	2.04E-05	-0.01 (-0.14,0.12)	-0.01 (-0.19,0.18)	0.93	-0.01 (-0.16,0.14)	1.02 (0.84,1.24)	0.851	0.978
VEGFC	-0.03 (-0.11,0.06)	5.23E-01	7.24E-01	0.42 (0.76,0.08)	0.27 (0.84,-0.31)	0.20	0.30 (0.70,-0.10)	0.86 (0.70,1.04)	0.125	0.625
VEGFR2	**0.12 (0.04,0.20)**	5.13E-03	2.01E-02	0.18 (0.05,0.31)	0.11 (-0.11,0.34)	0.15	0.13 (-0.03,0.28)	**0.83 (0.69,1.00)**	0.045	0.372
**Other proteins**										
Enzymes										
ADA	**0.17 (0.09,0.26)**	6.08E-05	5.48E-04	0.21 (0.08,0.35)	0.13 (-0.11,0.38)	0.10	0.15 (-0.01,0.31)	1.06 (0.87,1.30)	0.549	0.893
ARG1	**0.13 (0.05,0.21)**	1.50E-03	6.75E-03	-0.06 (-0.18,0.07)	-0.04 (-0.22,0.15)	0.62	-0.04 (-0.19,0.11)	**1.25 (1.02,1.51)**	0.029	0.365
CAIX	**-0.14 (-0.22,-0.05)**	1.29E-03	6.27E-03	-0.06 (-0.19,0.06)	-0.04 (-0.22,0.14)	0.62	-0.05 (-0.19,0.10)	1.00 (0.81,1.24)	0.974	0.997
CASP8	**0.18 (0.10,0.26)**	2.01E-05	2.01E-04	-0.19 (-0.31,-0.06)	-0.12 (-0.35,0.11)	0.13	-0.13 (-0.28,0.02)	**1.40 (1.16,1.70)**	0.001	0.052
GZMA	**0.14 (0.06,0.23)**	8.30E-04	4.98E-03	-0.08 (-0.22,0.07)	-0.05 (-0.26,0.17)	0.62	-0.05 (-0.22,0.12)	0.96 (0.79,1.16)	0.672	0.902
GZMB	**0.12 (0.03,0.21)**	6.76E-03	2.37E-02	0.30 (0.14,0.45)	0.19 (-0.13,0.50)	0.07	0.21 (0.03,0.39)	0.94 (0.77,1.14)	0.503	0.893
GZMH	0.05 (-0.04,0.13)	2.92E-01	5.15E-01	0.12 (-0.03,0.27)	0.08 (-0.15,0.30)	0.42	0.09 (-0.09,0.26)	0.98 (0.81,1.19)	0.860	0.978
HO1	0.08 (-0.00,0.17)	5.99E-02	1.54E-01	0.32 (0.18,0.45)	0.20 (-0.11,0.51)	0.08	0.23 (0.06,0.39)	1.06 (0.87,1.29)	0.565	0.893
MMP7	0.02 (-0.07,0.10)	7.24E-01	9.05E-01	-0.45 (-0.22,-0.69)	-0.29 (0.19,-0.76)	0.06	-0.32 (-0.04,-0.60)	1.00 (0.81,1.24)	0.979	0.997
MMP12	-0.00 (-0.08,0.07)	9.69E-01	9.91E-01	-0.08 (-0.28,0.11)	-0.05 (-0.34,0.23)	0.63	-0.06 (-0.29,0.17)	1.15 (0.91,1.45)	0.234	0.733
NOS3	0.08 (-0.01,0.16)	7.80E-02	1.88E-01	-0.26 (-0.38,-0.14)	-0.17 (-0.44,0.11)	0.07	-0.19 (-0.33,-0.04)	1.13 (0.94,1.35)	0.185	0.733
Membrane proteins										
CD4	0.06 (-0.03,0.15)	1.67E-01	3.41E-01	-4.42 (-2.49,-6.35)	-2.80 (1.57,-7.18)	0.08	-3.15 (-0.90,-5.40)	0.99 (0.82,1.20)	0.951	0.997
CD5	0.08 (-0.00,0.17)	5.32E-02	1.41E-01	-0.32 (-0.45,-0.19)	-0.20 (-0.52,0.11)	0.06	-0.23 (-0.38,-0.07)	0.93 (0.77,1.14)	0.494	0.893
CD8A	-0.02 (-0.10,0.07)	7.17E-01	9.05E-01	-0.06 (-0.16,0.03)	-0.04 (-0.18,0.10)	0.53	-0.04 (-0.16,0.07)	0.93 (0.76,1.14)	0.474	0.893
CD28	0.04 (-0.05,0.13)	3.48E-01	5.59E-01	-0.20 (-0.29,-0.10)	-0.13 (-0.33,0.08)	0.06	-0.14 (-0.25,-0.03)	**0.75 (0.59,0.96)**	0.021	0.308
CD83	0.09 (0.00,0.17)	4.26E-02	1.20E-01	-0.06 (-0.19,0.07)	-0.04 (-0.22,0.15)	0.62	-0.04 (-0.19,0.11)	1.06 (0.88,1.27)	0.551	0.893
CD244	0.04 (-0.04,0.12)	3.64E-01	5.72E-01	0.02 (-0.11,0.15)	0.01 (-0.17,0.20)	0.88	0.01 (-0.14,0.17)	**0.76 (0.62,0.92)**	0.005	0.121
CRTAM	-0.03 (-0.11,0.05)	4.35E-01	6.42E-01	-0.50 (-0.68,-0.31)	-0.31 (-0.79,0.16)	0.06	-0.35 (-0.57,-0.14)	0.97 (0.78,1.20)	0.775	0.929
ICOSLG	-0.08 (-0.16,0.01)	6.97E-02	1.74E-01	-0.25 (-0.37,-0.12)	-0.16 (-0.41,0.10)	0.07	-0.18 (-0.32,-0.03)	0.81 (0.62,1.05)	0.103	0.547
KLRD1	0.04 (-0.04,0.12)	3.42E-01	5.59E-01	0.22 (0.10,0.34)	0.14 (-0.10,0.37)	0.05	0.15 (0.01,0.29)	1.01 (0.83,1.23)	0.895	0.984
LAMP3	-0.01 (-0.08,0.06)	7.90E-01	9.45E-01	0.07 (0.22,-0.08)	0.05 (0.27,-0.18)	0.62	0.05 (0.23,-0.13)	1.24 (0.99,1.55)	0.066	0.456
NCR1	0.06 (-0.03,0.14)	1.94E-01	3.87E-01	-0.42 (-0.60,-0.24)	-0.27 (-0.69,0.15)	0.07	-0.30 (-0.51,-0.09)	0.96 (0.80,1.17)	0.713	0.904
MIC-A/B	0.04 (-0.05,0.13)	3.47E-01	5.59E-01	-0.19 (-0.36,-0.03)	-0.12 (-0.40,0.15)	0.23	-0.14 (-0.33,0.05)	1.00 (0.82,1.21)	0.975	0.997

© 2020 Pang et al. *JAMA Cardiology.*

Protein	Observational				MR-IPD	MR-Egger		Weighted median		Observational: proteins and CVD		
	SD difference (95% CI)[1]	Uncorrected *p*-value	FDR-adjusted *p*-value		SD difference (95% CI)[2]	SD difference (95% CI)[2]	*p* for intercept	SD difference (95% CI)[2]		HR (95% CI)[3]	Uncorrected *p*-value	FDR-adjusted *p*-value
ADGRG1	**0.11 (0.03,0.20)**	9.81E-03	3.04E-02		0.02 (-0.12,0.16)	0.01 (-0.18,0.21)	0.88	0.01 (-0.15,0.17)		1.09 (0.92,1.29)	0.304	0.761
TIE2	0.01 (-0.07,0.09)	8.18E-01	9.45E-01		-0.28 (-0.45,-0.11)	-0.18 (-0.49,0.14)	0.08	-0.20 (-0.39,0.00)		0.88 (0.72,1.07)	0.203	0.733
PD-L1	0.03 (-0.05,0.12)	4.66E-01	6.76E-01		-0.34 (-0.49,-0.18)	-0.21 (-0.55,0.13)	0.07	-0.24 (-0.42,-0.06)		1.16 (0.98,1.36)	0.082	0.526
PD-L2	0.00 (-0.08,0.08)	9.84E-01	9.93E-01		-0.18 (-0.27,-0.09)	-0.11 (-0.30,0.07)	0.08	-0.13 (-0.23,-0.03)		0.95 (0.78,1.15)	0.615	0.893
PDCD1	0.02 (-0.07,0.11)	6.69E-01	8.75E-01		-0.31 (-0.45,-0.17)	-0.20 (-0.51,0.12)	0.09	-0.22 (-0.38,-0.06)		0.97 (0.82,1.15)	0.708	0.904
Extracellular proteins												
Gal1	**0.29 (0.21,0.37)**	7.51E-13	3.38E-11		0.06 (-0.07,0.19)	0.04 (-0.15,0.23)	0.62	0.04 (-0.11,0.20)		1.04 (0.83,1.29)	0.750	0.921
Gal9	**0.20 (0.12,0.27)**	8.01E-07	1.80E-05		0.42 (0.28,0.56)	0.27 (-0.12,0.66)	0.06	0.30 (0.13,0.47)		1.19 (0.97,1.46)	0.101	0.547
DCN	**0.14 (0.06,0.23)**	5.92E-04	4.10E-03		-0.18 (-0.32,-0.05)	-0.12 (-0.35,0.12)	0.14	-0.13 (-0.29,0.02)		1.01 (0.84,1.22)	0.907	0.984

[1] SD difference of protein biomarkers per 1-SD higher baseline BMI.
[2] SD difference of protein biomarkers per 1-SD higher genetically-instrumented BMI.
[3] HR of CVD per 1-SD higher protein biomarkers.

© 2020 Pang et al. *JAMA Cardiology.*

eTable 8. Genetic associations of MCP1, HGF, IL18, and TRAIL with risk of CVD

	Ischaemic stroke		CHD
	OR (95% CI)		OR (95% CI)
MCP1			
**All 38 (*trans*) SNPs**	**1.08 (1.03, 1.13)**		**1.04 (0.99, 1.09)**
**Leave-one-out analysis**			
rs10145849	1.08 (1.03, 1.13)		1.04 (0.99, 1.10)
rs10744620	1.08 (1.03, 1.13)		1.04 (0.99, 1.10)
rs10888395	1.08 (1.03, 1.13)		1.03 (0.98, 1.08)
rs111995966	1.08 (1.03, 1.13)		1.04 (0.99, 1.09)
rs112313229	1.08 (1.03, 1.13)		1.03 (0.98, 1.09)
rs115936758	1.08 (1.03, 1.13)		1.04 (0.98, 1.09)
rs116425179	1.08 (1.03, 1.13)		1.04 (0.98, 1.09)
rs11710798	1.08 (1.03, 1.13)		1.03 (0.98, 1.07)
rs11720094	1.08 (1.03, 1.13)		1.03 (0.98, 1.07)
rs11926788	1.08 (1.03, 1.13)		1.03 (0.98, 1.07)
rs12047264	1.08 (1.03, 1.13)		1.03 (0.98, 1.09)
rs12073356	1.09 (1.04, 1.14)		1.04 (0.99, 1.10)
rs12727764	1.08 (1.03, 1.13)		1.03 (0.98, 1.09)
rs138591544	1.09 (1.04, 1.14)		1.04 (0.99, 1.10)
rs1386930	1.07 (1.02, 1.12)		1.04 (0.99, 1.10)
rs141676607	1.08 (1.03, 1.13)		1.04 (0.99, 1.10)
rs142043796	1.07 (1.02, 1.12)		1.03 (0.98, 1.09)
rs145155829	1.07 (1.02, 1.13)		1.04 (0.98, 1.09)
rs146522229	1.07 (1.02, 1.13)		1.04 (0.98, 1.09)
rs191688264	1.08 (1.03, 1.13)		1.04 (0.99, 1.10)
rs2036297	1.07 (1.02, 1.12)		1.04 (0.99, 1.10)
rs2281300	1.08 (1.03, 1.13)		1.04 (0.99, 1.10)
rs2712431	1.07 (1.02, 1.12)		1.03 (0.98, 1.09)
rs34190208	1.07 (1.02, 1.13)		1.04 (0.99, 1.10)
rs35060576	1.07 (1.02, 1.12)		1.04 (0.99, 1.10)
rs35333710	1.07 (1.02, 1.13)		1.04 (0.98, 1.09)
rs3774641	1.07 (1.02, 1.13)		1.03 (0.98, 1.09)
rs41338844	1.07 (1.02, 1.13)		1.04 (0.98, 1.09)
rs4682860	1.08 (1.03, 1.13)		1.05 (0.99, 1.10)
rs56212190	1.08 (1.03, 1.13)		1.04 (0.98, 1.09)
rs56300632	1.07 (1.02, 1.12)		1.04 (0.99, 1.10)
rs62242985	1.08 (1.03, 1.13)		1.03 (0.98, 1.09)
rs7019112	1.08 (1.02, 1.13)		1.04 (0.98, 1.09)
rs7197349	1.07 (1.02, 1.13)		1.04 (0.99, 1.10)
rs7519506	1.09 (1.04, 1.14)		1.05 (1.00, 1.10)
rs75265958	1.08 (1.03, 1.13)		1.04 (0.99, 1.09)
rs7527322	1.08 (1.03, 1.13)		1.04 (0.99, 1.10)
rs78629618	1.08 (1.03, 1.13)		1.04 (0.99, 1.10)
HGF			
**2 cis SNPs**	**1.02 (0.93, 1.12)**		**0.97 (0.85, 1.11)**
**2 trans SNPs***	**1.12 (0.99, 1.26)**		**1.07 (0.93, 1.22)**
**4 SNPs**	**1.06 (0.98, 1.14)**		**1.02 (0.93, 1.12)**
**Leave-one-out analysis**			
rs5745687	1.12 (0.99, 1.26)		1.07 (0.91, 1.26)
rs5745695	1.10 (1.01, 1.20)		1.06 (0.96, 1.17)
rs3748034	1.11 (1.00, 1.23)		1.09 (0.96, 1.23)
rs762861	1.09 (0.98, 1.20)		1.04 (0.93, 1.17)

*P-values for heterogeneity comparing SNPs in *cis* and *trans* loci: 0.73 for ischaemic stroke, 0.88 for CHD.
SNPs in *cis* loci are highlighted.

© 2020 Pang et al. *JAMA Cardiology.*

Abbreviation: CHD, coronary heart disease.

© 2020 Pang et al. *JAMA Cardiology.*

eTable 8. continued

	Ischaemic stroke		CHD
	**OR (95% CI)**		**OR (95% CI)**
IL18			
**1 cis SNP**	1.03 (0.95, 1.12)		1.02 (0.94, 1.12)
**5 trans SNPs***	1.01 (0.95, 1.07)		0.99 (0.93, 1.05)
**6 SNPs**	1.02 (0.97, 1.07)		1.00 (0.95, 1.05)
**Leave-one-out analysis**			
rs71478720	1.01 (0.95, 1.07)		0.99 (0.93, 1.05)
rs12470143	1.01 (0.96, 1.07)		1.01 (0.95, 1.06)
rs141091241	1.03 (0.98, 1.08)		1.00 (0.95, 1.05)
rs149612950	1.02 (0.97, 1.07)		1.00 (0.95, 1.05)
rs449348	1.02 (0.97, 1.08)		1.01 (0.96, 1.07)
rs55772679	1.01 (0.96, 1.07)		0.99 (0.94, 1.05)
TRAIL			
**1 cis SNP**	1.06 (0.74, 1.52)		1.18 (0.78, 1.77)
**22 trans SNPs***	1.00 (0.97, 1.03)		1.03 (1.01, 1.06)
**23 SNPs**	1.00 (0.97, 1.03)		1.03 (1.01, 1.06)
**Leave-one-out analysis**			
rs76769120	1.00 (0.97, 1.03)		1.03 (1.00, 1.06)
rs11081739	1.00 (0.98, 1.03)		1.03 (1.00, 1.06)
rs112324482	1.00 (0.97, 1.03)		1.04 (1.01, 1.07)
rs112821861	1.00 (0.98, 1.03)		1.02 (0.99, 1.05)
rs112827182	1.00 (0.97, 1.03)		1.04 (1.01, 1.07)
rs117153803	1.00 (0.97, 1.02)		1.03 (1.00, 1.06)
rs12457501	1.00 (0.97, 1.03)		1.04 (1.01, 1.07)
rs12607805	1.00 (0.97, 1.02)		1.03 (1.00, 1.06)
rs142552223	1.00 (0.97, 1.03)		1.03 (1.00, 1.06)
rs2912332	1.00 (0.97, 1.02)		1.04 (1.01, 1.07)
rs3136596	1.00 (0.97, 1.03)		1.03 (1.00, 1.07)
rs60035989	1.00 (0.97, 1.02)		1.03 (1.00, 1.06)
rs62093482	1.01 (0.98, 1.04)		1.04 (1.01, 1.07)
rs62093540	1.00 (0.97, 1.03)		1.03 (1.00, 1.07)
rs62093947	1.00 (0.97, 1.02)		1.04 (1.01, 1.07)
rs6506939	1.00 (0.97, 1.03)		1.03 (1.00, 1.06)
rs7233927	1.00 (0.97, 1.02)		1.03 (1.00, 1.06)
rs72917600	1.00 (0.97, 1.03)		1.04 (1.01, 1.07)
rs73408359	1.00 (0.97, 1.02)		1.03 (1.00, 1.07)
rs74778900	1.00 (0.97, 1.02)		1.03 (1.00, 1.07)
rs75489499	1.00 (0.97, 1.03)		1.03 (1.00, 1.06)
rs79325758	1.00 (0.97, 1.03)		1.03 (1.00, 1.07)
rs9946486	1.00 (0.97, 1.03)		1.03 (1.00, 1.06)

*P-values for heterogeneity comparing SNPs on *cis* and *trans* loci: IL18: 0.70 for ischaemic stroke, 0.58 for CHD; TRAIL: 0.73 for ischaemic stroke, 0.51 for CHD.
SNPs on *cis* loci are highlighted.
Abbreviation: CHD, coronary heart disease.

© 2020 Pang et al. *JAMA Cardiology.*

eTable 9. *P*-values from the MR Steiger test

Protein	Uncorrected *p*-value	FDR-adjusted *p*-value	Correct causal direction
ADA	8.63E-140	8.63E-140	TRUE
ADGRG1	4.34E-162	5.16E-161	TRUE
ANG1	1.25E-157	3.66E-157	TRUE
ANGPT2	3.48E-161	3.14E-160	TRUE
ARG1	1.62E-146	1.68E-146	TRUE
CAIX	5.62E-156	1.26E-155	TRUE
CASP8	2.78E-152	3.38E-152	TRUE
CCL3	4.78E-153	6.23E-153	TRUE
CCL4	2.87E-153	3.91E-153	TRUE
CCL17	7.92E-152	9.26E-152	TRUE
CCL19	6.07E-156	1.33E-155	TRUE
CCL20	7.46E-159	2.92E-158	TRUE
CCL23	3.67E-160	1.94E-159	TRUE
CD4	4.59E-162	5.16E-161	TRUE
CD5	1.71E-152	2.14E-152	TRUE
CD8A	8.84E-148	9.25E-148	TRUE
CD27	3.87E-157	1.05E-156	TRUE
CD28	6.31E-161	4.74E-160	TRUE
CD40	8.71E-161	5.60E-160	TRUE
CD40L	2.68E-158	9.55E-158	TRUE
CD70	3.21E-162	4.81E-161	TRUE
CD83	9.50E-156	1.99E-155	TRUE
CD244	5.59E-160	2.79E-159	TRUE
CRTAM	1.16E-155	2.37E-155	TRUE
CSF1	6.38E-154	9.27E-154	TRUE
CX3CL1	4.00E-161	3.27E-160	TRUE
CXCL1	2.42E-159	9.90E-159	TRUE
CXCL5	1.04E-156	2.68E-156	TRUE
CXCL9	3.26E-154	5.06E-154	TRUE
CXCL10	1.36E-153	1.94E-153	TRUE
CXCL11	3.12E-151	3.55E-151	TRUE
CXCL12	3.31E-153	4.44E-153	TRUE
CXCL13	1.90E-149	2.08E-149	TRUE
DCN	2.93E-155	5.53E-155	TRUE
EGF	6.91E-160	3.11E-159	TRUE
FasLG	2.70E-154	4.27E-154	TRUE
FGF2	4.19E-155	7.55E-155	TRUE
Gal1	1.49E-161	1.49E-160	TRUE
Gal9	8.41E-149	8.91E-149	TRUE
GZMA	1.45E-145	1.46E-145	TRUE
GZMB	2.08E-156	4.93E-156	TRUE
GZMH	8.39E-152	9.68E-152	TRUE
HGF	6.95E-161	4.81E-160	TRUE

© 2020 Pang et al. *JAMA Cardiology.*

HO1	3.11E-152	3.74E-152	TRUE
**Protein**	**Uncorrected *p*-value**	**FDR-adjusted *p*-value**	**Correct causal direction**
ICOSLG	7.01E-149	7.51E-149	TRUE
IFN-beta	2.25E-156	5.19E-156	TRUE
IFN-gamma	5.22E-157	1.38E-156	TRUE
IL1-alpha	7.34E-153	9.44E-153	TRUE
IL2	9.03E-159	3.39E-158	TRUE
IL4	7.22E-165	2.17E-163	TRUE
IL5	4.02E-158	1.34E-157	TRUE
IL6	4.40E-153	5.83E-153	TRUE
IL7	3.32E-155	6.10E-155	TRUE
IL8	6.52E-155	1.13E-154	TRUE
IL10	1.96E-155	3.84E-155	TRUE
IL12	1.58E-156	3.96E-156	TRUE
IL12-RB1	1.23E-154	2.02E-154	TRUE
IL13	2.18E-152	2.68E-152	TRUE
IL18	6.03E-155	1.06E-154	TRUE
IL33	2.28E-149	2.47E-149	TRUE
KLRD1	1.01E-154	1.69E-154	TRUE
LAMP3	1.74E-153	2.45E-153	TRUE
LAP-TGF-beta1	1.25E-155	2.50E-155	TRUE
MCP1	9.77E-153	1.24E-152	TRUE
MCP2	2.40E-153	3.32E-153	TRUE
MCP3	3.50E-160	1.94E-159	TRUE
MCP4	3.75E-157	1.05E-156	TRUE
MIC-A/B	1.04E-163	2.35E-162	TRUE
MMP7	6.32E-146	6.46E-146	TRUE
MMP12	3.68E-154	5.52E-154	TRUE
NCR1	6.56E-160	3.11E-159	TRUE
NOS3	1.22E-167	9.65E-166	TRUE
PD-L1	6.62E-152	7.83E-152	TRUE
PD-L2	5.85E-158	1.88E-157	TRUE
PDCD1	1.26E-157	3.66E-157	TRUE
PDGF_subunit_b	6.14E-158	1.91E-157	TRUE
PGF	9.74E-160	4.17E-159	TRUE
PTN	2.81E-150	3.13E-150	TRUE
TIE2	6.82E-155	1.16E-154	TRUE
TNF	4.39E-154	6.47E-154	TRUE
TNFRSF4	1.70E-156	4.14E-156	TRUE
TNFRSF9	2.76E-158	9.55E-158	TRUE
TNFRSF12A	8.23E-163	1.48E-161	TRUE
TNFRSF21	3.45E-154	5.27E-154	TRUE
TNFSF14	1.74E-150	1.96E-150	TRUE
TRAIL	2.95E-155	5.53E-155	TRUE
TWEAK	1.22E-160	7.30E-160	TRUE

© 2020 Pang et al. *JAMA Cardiology.*

VEGFA	7.04E-156	1.51E-155	TRUE
VEGFC	1.26E-154	2.03E-154	TRUE
**Protein**	**Uncorrected *p*-value**	**FDR-adjusted *p*-value**	**Correct causal direction**
VEGFR2	2.15E-167	9.65E-166	TRUE

© 2020 Pang et al. *JAMA Cardiology.*

eTable 10. Randomised controlled trials of anti-inflammatory therapy in CVD

Drug	Target	Study population	Indication	Main findings	PMID
Canakinumab	ILβ	Treatment group: 6717 patients	Prior AMI with CRP >2 mg/l (at least 30 days after AMI)	1) Reduction in CRP levels at 48 months	28845751
		Control group: 3344 patients		2) Reduction in IL6 levels at 12 months	
				3) Reduction in MACE (150-mg group)	
Anakinra	IL1Ra	Treatment group: 93 patients<br>Control group: 89 patients	Patients with non-ST elevation acute coronary syndrome	1) Reduction in CRP levels at 7 days	25079365
				2) Reduction in CRP and IL6 levels at 14 days	
				3) MACE at day 30 and 3 months was similar	
				4) A significant excess of MACE at 1 year in the treatment group	
Methotrexate	IL6, TNF	Treatment group: 2391 patients<br>Control group: 2395 patients	Prior MI or multi-vessel coronary disease with either type 2 diabetes or metabolic syndrome	1) No reduction in IL1β, IL6, and CRP at 2.3 years	30415610
				2) No reduction in MACE at 2.3 years	
Colchicine	Multiple	Treatment group: 282 patients<br>Control group: 250 patients	Patients with stable CHD receiving aspirin and/or clopidogrel and statins	1) Reduction in the composite incidence of ACS, out-of-hospital	23265346
				2) Reduction in cardiac arrest, or non-cardioembolic ischaemic stroke at 3 years	
Colchicine	Multiple	Treatment group: 77 patients	ST-segment elevation AMI	1) Reduction in peak CRP level	26265659
		Control group: 74 patients		2) Reduction in infarction size at 6-9 days	
Colchicine	Multiple	Treatment group: 2366 patients<br>Control group: 2379 patients	Patients within 30 days after a MI	Reduction in ischaemic cardiovascular events	31733140
Tocilizumab	IL6R	Tocilizumab group: 1538 patients<br>Etanercept group: 1542 patients	Moderate to severe rheumatoid arthritis	No difference in MACE at 4.9 years	31469238

Abbreviations: MACE, major adverse cardiovascular event (a composite of non-fatal myocardial infarction, nonfatal stroke, or cardiovascular death); AMI, acute myocardial infarction.

© 2020 Pang et al. *JAMA Cardiology.*

eTable 11. Observational associations between BMI and proteins in CKB and the INTERVAL study*

	INTERVAL		CKB
**Protein**	**Effect size (95% CI) per 1 kg/m^2 higher BMI**		**Effect size (95% CI) per 1 kg/m^2 higher BMI**
IL6	0.027 (0.020, 0.035)		0.051 (0.030, 0.072)
CCL3	0.032 (0.025, 0.040)		0.019 (0.005, 0.034)
CCL19	0.026 (0.018, 0.033)		0.044 (0.024, 0.064)
CXCL10	0.018 (0.011, 0.026)		0.035 (0.015, 0.055)
MCP1	-0.012 (-0.0020, 0.0003)		0.018 (0.005, 0.030)
MCP3	0.006 (0.0001, 0.014)		0.015 (0.006, 0.025)
TNFSF14	0.017 (0.009, 0.024)		0.040 (0.028, 0.053)
TWEAK	-0.015 (-0.0023, -0.0008)		-0.013 (-0.021, -0.006)
HGF	0.048 (0.040, 0.055)		0.038 (0.029, 0.047)
PGF	0.019 (0.012, 0.027)		0.015 (0.006, 0.023)
VEGFA	0.003 (-0.005, 0.010)		0.024 (0.014, 0.034)
VEGFR2	0.015 (0.008, 0.022)		0.009 (0.003, 0.016)
DCN	-0.005 (-0.012, 0.003)		0.011 (0.005, 0.017)
Gal1	-0.004 (-0.012, 0.003)		0.021 (0.015, 0.027)

* Protein values were rank-inverse normalised before performing linear regression of protein biomarkers on BMI. Estimates in the INTERVAL study adjusted for age and sex. Estimates in CKB adjusted for age, age squared, sex, region, smoking, alcohol, education, household income, self-rated health, systolic blood pressure, diabetes, statin treatment, prior kidney disease, and fasting time. The SomaScan assay was used in the INTERVAL study (Sun BB, Maranville JC, Peters JE, Stacey D, Staley JR, Blackshaw J, et al. Genomic atlas of the human plasma proteome. *Nature*. 2018;558(7708):73-79).

© 2020 Pang et al. *JAMA Cardiology.*

eMethods

Assessment of adiposity

All anthropometric measurements were taken by trained technicians while participants were wearing light clothes and no shoes, usually to the nearest 0.1 cm or 0.1 kg. Standing height and sitting height were measured using a stadiometer. Sitting height was measured as the length of the body from buttocks to the crown of the head. Weight was measured using a body composition analyser (TANITA-TBF-300GS; Tanita Corporation), with subtraction of weight of clothing according to season (ranging from 0.5 kg in summer to 2.0-2.5 kg in winter). Waist circumference (WC) and hip circumference (HC) were measured using a soft non-stretchable tape, with HC measured at the maximum circumference around the buttocks. Body mass index (BMI) was calculated as the measured weight in kilograms divided by the square of the measured height in metres. BMI at 25 years (BMI25) was calculated using the recalled weight at age 25 years and the measured height at baseline. Waist-to-hip ratio (WHR) was the ratio of WC to HC. Percent body fat (%BF) was the fraction of total weight that was estimated to be fat weight by the Tanita body composition analyser using proprietary algorithms. Other derived measures of adiposity included fat mass (the product of %BF and body weight), lean mass (the subtraction of fat mass from body weight), waist-height ratio (WC divided by height), and leg length (the subtraction of sitting height from standing height).

OLINK proteomics

Details of the OLINK assay have been reported elsewhere, including measurements of assay performance and validation.[1,2] Briefly, a 96-well microplate format is used, including 88 samples and eight external quality control standards (two pooled plasma controls, three blank samples used to set the LOD and three inter-plate controls used for normalization). Each sample is mixed with oligonucleotide-labelled antibodies and four internal controls.

© 2020 Pang et al. *JAMA Cardiology.*

When both high-specificity antibodies bind the target protein in the sample, the attached oligonucleotides form a unique DNA reporter sequence that is subsequently amplified and quantified by quantitative polymerase chain reaction (qPCR). qPCR data are pre-processed to generate NPX values.

In addition, eight external controls were ran on every plate (**eFigure 19**).[3,4] External controls were added as follows. First, a pooled plasma sample containing the 92 proteins (inter-plate control, same design as the extension control) was run in triplicate in each plate to monitor inter-plate variability and for normalization. Second, negative controls (i.e. negative buffer samples) were run in triplicate in each plate. The background signal was used to calculate the limit of detection (LOD) for each protein. The LOD was calculated as the mean of 3 negative controls plus 3SD. Fixed SDs were calculated during validation of OLINK panels and probe lots and were based on runs of negative controls (at least 36 replicates). For multi-plate studies, the maximum observed LOD for each assay is chosen and reported as the study LOD. Third, a pooled plasma sample was run in duplicate in each plate to calculate intra- and inter-assay %CV. The %CV was calculated on linear NPX values (2^NPX). Intra- and inter-assay CV were reported for each assay.

eFigure 19. Internal and external controls

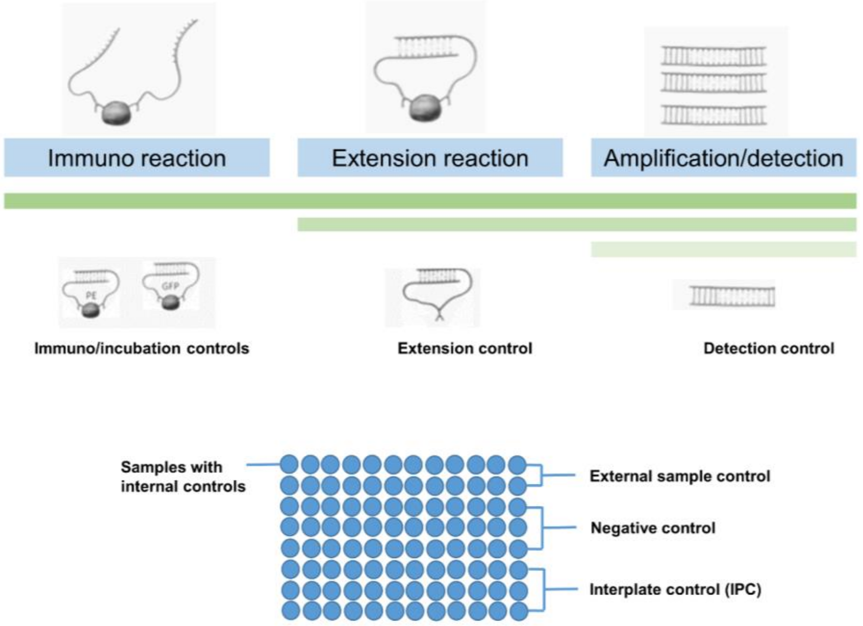

* Adapted from OLINK validation document.[3,4]

© 2020 Pang et al. *JAMA Cardiology.*

The specificity of the OLINK assays has been described in the validation document.[3] For each protein target, two oligonucleotide-coupled antibodies (PEA probes) must bind in close enough proximity to enable the oligos to hybridize and form a unique DNA template for detection by qPCR. The 92-antibody pairs selected in the Immuno-Oncology panel have been evaluated against 92-panel specific proteins as well as an additional 107 proteins. These 107 proteins were selected from a library of protein biomarkers included in other OLINK panels (e.g. inflammation panel). This was done by creating a pool of antigens, which was incubated across all 92-antibody protein pairs for the panel. Only if there was a correct match would a reporter sequence be created and serve as a template for subsequent real-time qPCR. Ten sub-pools of antigen were evaluated to cover the 92 assays in the OLINK panel as illustrated in the validation document.[3]

eFigure 20. Assay specificity

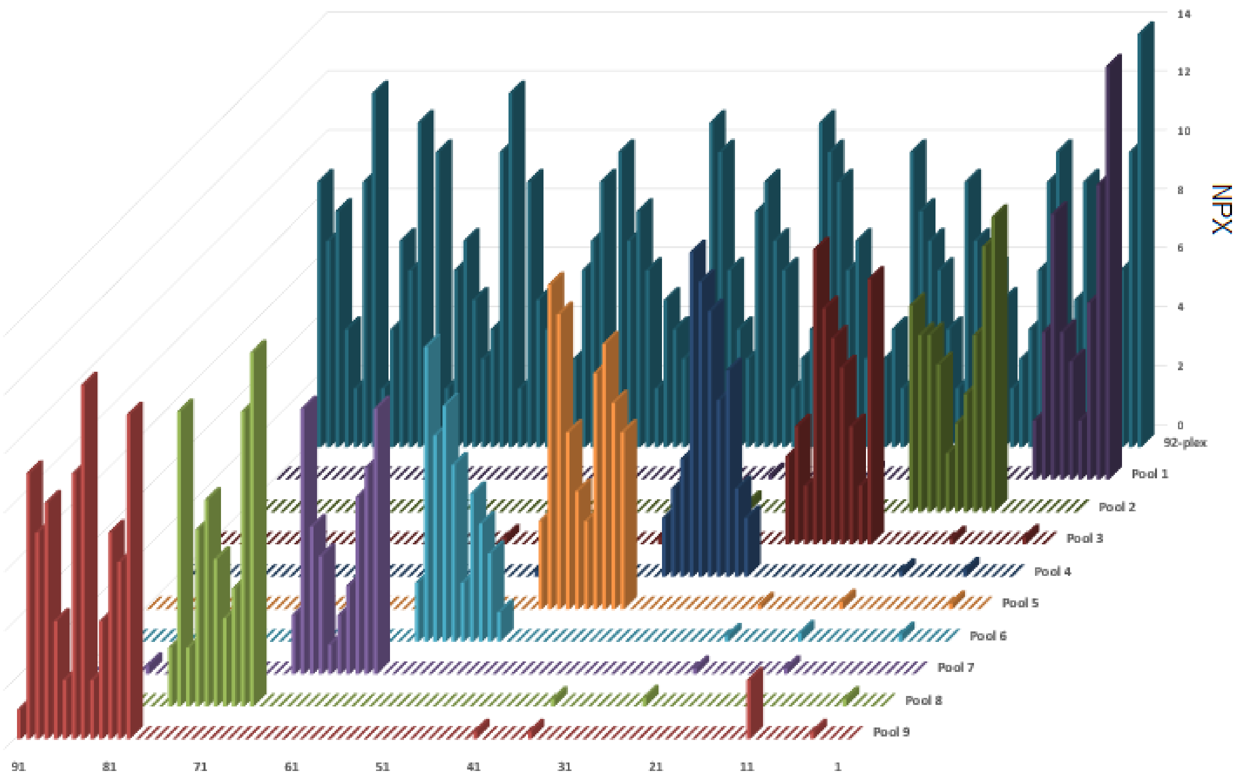

* Values in this figure are for illustration purpose and do not show the actual values reported in the OLINK validation studies.[3]

© 2020 Pang et al. *JAMA Cardiology.*

Statistical analysis

Observational analyses

In observational analysis involving 628 participants, linear regression was used to assess the associations of adiposity with protein markers, adjusted for age at baseline, age squared, sex, area (10 regions), education (6 groups: no formal school, primary school, middle school, high school, technical school/college, or university), household income (6 groups: <2500, 2500-4999, 5000-9999, 10,000-19,999, 20,000-34,999, or ≥35,000 RMB), alcohol (6 groups: abstainers, ex-weekly drinkers, reduced-intake drinkers, occasional drinkers, and among weekly drinkers, <20 or ≥20 g/day [women], <30 or ≥30 g/day [men]), self-rated health (3 groups: poor, fair, and good), systolic blood pressure (SBP, continuous), diabetes, statin treatment (3 groups: no CVD, CVD on statin treatment, and CVD not on statin treatment), prior kidney disease (yes or no), and fasting time (continuous). Statin treatment was assessed among patients with prior CVD at baseline. Prior kidney disease was assessed by the question “Has a doctor ever told you that you had had kidney disease?”. In prospective analyses of associations of protein levels with risk of CVD, Cox proportional hazards models were used to estimate hazard ratios (HRs) of vascular disease per 1-SD higher protein markers, adjusted for the same variables as in the analysis of adiposity and protein markers.

In prospective analyses of associations of protein levels with risk of CVD, Cox proportional hazards models were used to estimate hazard ratios (HRs) of vascular disease per 1-SD higher protein markers, adjusted for the same variables as in the analysis of adiposity and protein markers. To estimate the extent to which additional adjustment for protein biomarkers might influence the observational association of BMI with CVD (as an approximation to the quantification of potential mediation), we extended the base model to include proteins associated with both BMI and CVD risk and examined the percent change

© 2020 Pang et al. *JAMA Cardiology.*

in the log HRs per 1-SD higher BMI. The proportional change in CVD logHR associated with high BMI due to additional adjustment for protein biomarkers was calculated as follows: $((logHR_{basic\ model} - logHR_{adjusted\ model}) / (logHR_{basic\ model}))$ X 100%.

We used the following approach to account for multiple comparisons. Significance was assessed at 5% false discovery rate (FDR)[5] in the observational analysis of BMI with protein biomarkers. Unadjusted *p*-values are reported for the genetic associations of BMI with protein biomarkers and for the observational associations of protein biomarkers with vascular events to avoid over-correction. The Mendelian randomization-derived effects of BMI on protein biomarkers were compared with the corresponding observational associations using Cochran's Q test (with significance assessed at 5% FDR). In addition, we used a method based on the Rényi decomposition as an alternative approach to visualise multiple comparisons for the observational associations of BMI with proteins and of proteins with vascular events. The isolation of protein biomarkers in the Rényi plot above the line of unit slope suggests presence of significant associations taking into account multiple testing.[6]

Subgroup analyses of the observational associations were conducted by sex, region (urban vs rural), smoking (ever vs never), physical activity (<21 vs ≥21 MET-h/day [median]), RPG (<5.6 vs ≥5.6 mmol/L [median]), and hypertension status (yes vs no). Hypertension was defined as: (i) previous diagnosis of hypertension; (ii) SBP ≥140 mmHg and DBP ≥90 mmHg; or, (iii) currently on anti-hypertensive medication.

Mendelian randomisation

Several sensitivity analyses were conducted for Mendelian randomisation analysis. First, we used MR-Egger and weighted median methods to explore whether our finding depends on the assumption that all the variants have no horizontal pleiotropic effects (i.e. the effects of BMI variants on multiple biological pathways). The MR-Egger method is a

© 2020 Pang et al. *JAMA Cardiology.*

statistical approach that allows one or more genetic variants to have pleiotropic effects, while the weighted median estimator can give valid estimates even in the presence of horizontal pleiotropy as long as at least half the genetic variants have no pleiotropic effects. The weighted median estimator retains greater precision in the estimates than MR-Egger (owing to a power penalty). Second, the genetic associations of BMI with proteomics were compared across different BMI scores (i.e. unweighted score and externally weighted scores using (a) weights from Biobank Japan and (b) meta-analysis of UKB and GIANT. Third, the associations of genetically-predicted BMI with protein biomarkers was also assessed by two-sample Mendelian randomisation using GWAS summary statistics from the GWAS of BMI in BBJ (i.e. SNP-BMI) together with summary-specific estimates in CKB (i.e. SNP-proteins). The derivation of the summary estimates in CKB used the same adjustment as the individual participant-level data (IPD) analysis. Inverse-variance weighted (IVW) analysis was performed by linear regression of the SNP-protein associations on the SNP-BMI associations. Fourth, the MR Steiger test was used to elucidate whether the assumption that the exposure causes the outcome is valid by testing whether the correlation of BMI SNPs in each protein biomarker was less than the correlation with BMI (**eTable 9**).[7] Fifth, we excluded from the unweighted score SNPs within 1M bp of the gene or genes encoding protein biomarkers.

Meta-analysis and two-sample Mendelian randomisation

In order to maximise sample size, we meta-analysed using fixed effect models (1) causal estimates between BMI and protein biomarkers with those reported by or calculated from previous studies (Analysis 1) and (2) our observational estimates between protein biomarkers and incident CVD with previous prospective studies (Analysis 2). Protein biomarkers were selected for exploration of causal effects if there were causal associations of BMI with proteins in CKB and available GWAS for these proteins (seven proteins: IL6, IL18, MCP1, MCP3, TRAIL, TWEAK, and HGF). We conducted two-sample

© 2020 Pang et al. *JAMA Cardiology.*

Mendelian randomisation analysis to estimate the causal associations of protein with CVD (Analysis 3). IL6, MCP1, and MCP3 were excluded from this analysis because no instruments were available. Instruments for protein biomarkers were extracted from four proteomics GWAS and a literature search in GWAS catalogue using the *p*-value threshold in the original studies (**eTable 12** on page 75-78, Supplement).[8-11] SNPs were included if they were not in linkage disequilibrium (r^2<0.01 in CEU) with one another and were in *cis* to the locus of interest (-/+ 300 kbp). *Trans* associated variants were also included as a sensitivity analysis. Selection criteria, data sources, and calculations are reported in **eTable 13** (on page 79, Supplement). In addition to CVD, we conducted two-sample Mendelian randomisation analysis to estimate the genetic association between IL18 and type 2 diabetes using summary-level statistics reported by DIAGRAM (34,840 cases and 114,981 controls).[12] We selected type 2 diabetes because a previous RCT suggested that IL18 might be a potential drug target.[13] As only one *cis*-pQTL was identified for IL18 (**eTable 12** on page 75-78, Supplement), the genetic association between IL18 and type 2 diabetes was calculated using the Wald ratio.

Colocalisation analysis

Colocalisation analysis was conducted for protein biomarkers for which we found some evidence of genetic association with CVD (i.e. TWEAK, HGF, and ischaemic stroke), using SNPs within 2kb of the protein-encoding locus and GWAS summary statistics for ischaemic stroke from MEGASTROKE in CEU.[14] For TWEAK and HGF, GWAS summary statistics were obtained from the INTERVAL study (in CEU).[10] We used the R package "coloc" to perform genetic colocalization analysis of two potentially related phenotypes and to assess whether they share common genetic causal variant(s) in a given region.[15] Because summary-level data was used, approximate Bayes factor (ABF) colocalisation analyses were conducted. Posterior probabilities of the following hypotheses were calcuated: H0: neither trait has a genetic association in the region; H1: only trait 1 has a

© 2020 Pang et al. *JAMA Cardiology.*

genetic association in the region; H2: only trait 2 has a genetic association in the region; H3: both traits are associated, but with different causal variants; H4: both traits are associated and share a single causal variant.[15] When visualising colocalisation analysis (using the R package “locuscomparer”), the *cis*-SNP with the smallest *p*-value was selected as the lead SNP.

References:

1. Lundberg M, Thorsen SB, Assarsson E, Villablanca A, Tran B, Gee N, et al. Multiplexed homogeneous proximity ligation assays for high-throughput protein biomarker research in serological material. *Mol Cell Proteomics*. 2011;10(4):M110004978. doi:10.1074/mcp.M110.004978.

2. Assarsson E, Lundberg M, Holmquist G, Bjorkesten J, Thorsen SB, Ekman D, et al. Homogenous 96-plex PEA immunoassay exhibiting high sensitivity, specificity, and excellent scalability. *PLoS One*. 2014;9(4):e95192. doi:10.1371/journal.pone.0095192.

3. OLINK validation document for the Immuno-Oncology panel (article number 95310). Available at: https://www.olink.com/resources-support/document-download-center/ (assessed 2018 December; the original document can only be available by contacting OLINK directly)

4. OLINK FAQs: how is quality control of the data performed? Available at: https://www.olink.com/question/how-is-quality-control-of-the-data-performed/ (assessed 2020 May)

5. Benjamini YH. Controlling the false discovery rate: a practical and powerful approach to multiple testing. *Journal of the Royal Statistical Society Series B (Methodological)*. 1995:289-300

6. Cox DR, Kartsonaki C. On the analysis of large numbers of *p*-values. International *Statistical Review*. 2019;87(3):505-513

7. Hemani G, Tilling K, and Davey Smith G. Orienting the causal relationship between imprecisely measured traits using GWAS summary data. *PLoS Genet*. 2017;13(11): e1007081. doi:1371/journal.pgen.1007081.

8. Ahola-Olli AV, Wurtz P, Havulinna AS, Aalto K, Pitkanen N, Lehtimaki T, et al. Genome-wide association study identifies 27 loci influencing concentrations of circulating cytokines and growth factors. *Am J Hum Genet*. 2017;100(1):40-50.

9. Folkersen L, Fauman E, Sabater-Lleal M, Strawbridge RJ, Franberg M, Sennblad B, et al. Mapping of 79 loci for 83 plasma protein biomarkers in cardiovascular disease. *PLoS Genet.* 2017;13(4):e1006706. doi:10.1371/journal.pgen.1006706.

10. Sun BB, Maranville JC, Peters JE, Stacey D, Staley JR, Blackshaw J, et al. Genomic atlas of the human plasma proteome. *Nature*. 2018;558(7708):73-79.

11. Chong M, Sjaarda J, Pigeyre M, Mohammadi-Shemirani P, Lali R, Shoamanesh A, et al. Novel drug targets for ischemic stroke identified through Mendelian randomization analysis of the blood proteome. *Circulation*. 2019;140(10);819-830.

© 2020 Pang et al. *JAMA Cardiology.*

12. Morris AP, Voight BF, Teslovich TM, Ferreira T, Segre AV, Steinthorsdottir, et al. Large-scale association analysis provides insights into the genetic architecture and pathophysiology of type 2 diabetes. *Nat Genet.* 2012;44(9):981-90.

13. McKie EA, Reid JL, Mistry PC, DeWall SL, Abberley L, Ambery PD, et al. A study to investigate the efficacy and safety of an anti-interleukin-18 monoclonal antibody in the treatment of type 2 diabetes mellitus. *PLoS One*. 2016;11:e0150018. doi: 10.1371/journal.pone.0150018.

14. Malik R, Chauhan G, Traylor M, Sargurupremraj M, Okada Y, Mishra A, et al. Multiancestry genome-wide association study of 520,000 subjects identifies 32 loci associated with stroke and stroke subtypes. *Nat Genet.* 2018;50(4):524-537.

15. Giambartolomei C. et al. Bayesian test for colocalisation between pairs of genetic association studies using summary statistics. *PLoS Genet*. 2014;10:e1004383. doi:10.1371/journal.pgen.1004383.

16. CARDIoGRAMplusC4D Consortium, M Nikpey, A Goel, H Won, LM Hall C. Willenborg, S Kanoni, D Saleheen et al. A comprehensive 1000 Genomes–based genome-wide association meta-analysis of coronary artery disease. *Nat Genet.* 2015;47(10):1121-1130.

17. Yengo L, Sidorenko J, Kemper KE, Zheng Z, Wood AR, Weedon MN, et al. Meta-analysis of genome-wide association studies for height and body mass index in ~700,000 individuals of European ancestry. *Hum Mol Genet*. 2018;27(20):3641-3649.

© 2020 Pang et al. *JAMA Cardiology.*

eTable 12. SNPs that were used as instruments for selected protein biomarkers

PMID	Protein	SNP	Position	Chr	Locus	Effect allele	Other allele	Effect size*	SE	*p*-value
27989323	HGF	rs5745687	81359051	7	HGF	C	T	0.307	0.041	2.75E-14
29875488	HGF	rs5745695	81358075	7	HGF	A	G	0.19	0.03	0.5481
27989323	HGF	rs3748034	3446091	4	HGFAC	T	G	-0.15	0.023	1.81E-10
27989323	HGF	rs762861	3442011	4	HGFAC	C	G	-0.108	0.019	1.56E-08
27989323	IL18	rs71478720	112009605	11	IL18	C	T	0.2669	0.0276	3.71E-22
27989323	IL18	rs12470143	31763558	2	unknown	C	T	-0.1496	0.0235	2.12E-10
27989323	IL18	rs141091241	112111460	11	unknown	C	T	0.4122	0.0728	1.83E-08
27989323	IL18	rs149612950	68166499	5	unknown	G	A	-0.505	0.0906	2.54E-08
27989323	IL18	rs449348	32499150	2	unknown	G	A	-0.1371	0.0243	1.90E-08
27989323	IL18	rs55772679	112235908	11	unknown	C	T	0.1393	0.024	6.86E-09
29875488, 31208196	TWEAK	rs11078691	7424935	17	TWEAK	T	C	0.13	0.033	0.016408
29875488, 31208196	TWEAK	rs117646332	7656668	17	TWEAK	A	G	0.316	0.05	8.03E-05
29875488, 31208196	TWEAK	rs118081933	7606211	17	TWEAK	A	G	-0.273	0.092	0.081268
29875488, 31208196	TWEAK	rs12451505	7625888	17	TWEAK	C	G	0.15	0.028	0.000785
29875488, 31208196	TWEAK	rs12937133	7289042	17	TWEAK	T	C	0.083	0.026	0.056135
29875488, 31208196	TWEAK	rs12941509	7448288	17	TWEAK	G	C	0.393	0.027	7.44E-22
29875488, 31208196	TWEAK	rs150836621	7516177	17	TWEAK	T	G	0.183	0.033	0.794534
29875488, 31208196	TWEAK	rs1614984	7571452	17	TWEAK	A	G	0.188	0.025	1.78E-06
29875488, 31208196	TWEAK	rs1641511	7559677	17	TWEAK	A	G	0.26	0.029	1.16E-08
29875488, 31208196	TWEAK	rs181975550	7595379	17	TWEAK	T	C	-0.424	0.075	0.000418
29875488, 31208196	TWEAK	rs2078486	7583083	17	TWEAK	A	G	-0.074	0.049	0.410656
29875488, 31208196	TWEAK	rs33989543	7324788	17	TWEAK	A	G	-0.146	0.048	0.070651
29875488, 31208196	TWEAK	rs3803802	7621464	17	TWEAK	A	C	-0.146	0.025	0.000277
29875488, 31208196	TWEAK	rs56206519	7303143	17	TWEAK	A	G	0.081	0.025	0.057269
29875488, 31208196	TWEAK	rs56332843	7647537	17	TWEAK	A	G	-0.161	0.039	0.011796
29875488, 31208196	TWEAK	rs62062613	7642035	17	TWEAK	T	C	-0.137	0.038	0.027598
29875488, 31208196	TWEAK	rs6503016	7259073	17	TWEAK	T	C	0.077	0.026	0.076536

© 2020 Pang et al. *JAMA Cardiology.*

PMID	Protein	SNP	Position	Chr	Locus	Effect allele	Other allele	Effect size*	SE	*p*-value
29875488, 31208196	TWEAK	rs6608	7464413	17	TWEAK	T	C	-0.349	0.032	1.74E-12
29875488, 31208196	TWEAK	rs72842814	7314137	17	TWEAK	A	G	-0.382	0.076	0.001873
29875488, 31208196	TWEAK	rs9906416	7344263	17	TWEAK	T	C	-0.253	0.03	4.91E-08
28369058	TRAIL	rs76769120	12243825	1	TRAIL	G	C	0.42	0.001	7E-14
27989323	TRAIL	rs11081739	29583126	18	unknown	G	A	-0.1411	0.0202	3.34E-12
27989323	TRAIL	rs112324482	29534956	18	unknown	G	A	-0.1039	0.0174	2.21E-09
27989323	TRAIL	rs112821861	29569386	18	unknown	G	T	0.8518	0.0496	4.90E-68
27989323	TRAIL	rs112827182	171992068	3	unknown	G	A	-0.3025	0.041	2.88E-13
27989323	TRAIL	rs117153803	28735908	18	unknown	G	T	-0.1736	0.0295	4.90E-09
27989323	TRAIL	rs12457501	29836551	18	unknown	C	T	-0.2019	0.0273	1.44E-13
27989323	TRAIL	rs12607805	29117455	18	unknown	G	A	0.174	0.0254	8.20E-12
27989323	TRAIL	rs142552223	172234017	3	unknown	G	A	0.3823	0.0379	5.77E-24
27989323	TRAIL	rs2912332	28892506	18	unknown	G	A	0.2139	0.026	1.47E-16
27989323	TRAIL	rs3136596	172228872	3	unknown	G	A	-0.1147	0.0209	3.65E-08
27989323	TRAIL	rs60035989	29231813	18	unknown	C	A	-0.1062	0.0186	1.02E-08
27989323	TRAIL	rs62093482	29178899	18	unknown	C	T	-0.9987	0.0532	7.10E-78
27989323	TRAIL	rs62093540	29316653	18	unknown	C	T	-0.2919	0.0289	6.82E-24
27989323	TRAIL	rs62093947	29660305	18	unknown	C	T	0.7596	0.046	3.31E-61
27989323	TRAIL	rs6506939	29312725	18	unknown	C	T	-0.0927	0.0169	4.17E-08
27989323	TRAIL	rs7233927	29778804	18	unknown	G	A	-0.0913	0.0165	2.86E-08
27989323	TRAIL	rs72917600	27859593	18	unknown	C	A	-0.2458	0.0378	4.79E-11
27989323	TRAIL	rs73408359	28927298	18	unknown	C	T	-0.411	0.0365	2.55E-29
27989323	TRAIL	rs74778900	28086266	18	AC115100.1,AC090506.1	C	T	-0.5906	0.0532	2.59E-28
27989323	TRAIL	rs75489499	172503541	3	unknown	C	T	0.2052	0.0348	3.39E-09
27989323	TRAIL	rs79325758	28029396	18	unknown	C	T	-0.3632	0.0483	1.30E-13
27989323	TRAIL	rs9946486	28795414	18	unknown	G	A	-0.1798	0.0246	3.02E-13
27989323	MCP1	rs10145849	82941991	14	SEL1L	G	A	0.076	0.016	3.41E-06
27989323	MCP1	rs10744620	3739094	12	EFCAB4B	C	T	-0.079	0.016	9.91E-07
27989323	MCP1	rs10888395	150762171	1	CTSK	C	T	0.081	0.016	5.98E-07

© 2020 Pang et al. *JAMA Cardiology.*

27989323	MCP1	rs111995966	109174969	2	LIMS1	G	T	-0.145	0.031	2.53E-06
27989323	MCP1	rs112313229	46364860	3	CCR2	G	A	0.165	0.031	1.43E-07
**PMID**	**Protein**	**SNP**	**Position**	**Chr**	**Locus**	**Effect allele**	**Other allele**	**Effect size***	**SE**	***p*-value**
27989323	MCP1	rs115936758	159170343	1	LOC100131825	C	T	0.178	0.035	5.74E-07
27989323	MCP1	rs116425179	45598703	3	LARS2	G	A	0.148	0.026	5.68E-09
27989323	MCP1	rs11710798	48570686	3	PFKFB4	C	A	-0.092	0.02	2.87E-06
27989323	MCP1	rs11720094	46559911	3	LRRC2	G	C	0.106	0.016	1.54E-11
27989323	MCP1	rs11926788	42623498	3	SEC22C	G	C	0.189	0.036	2.06E-07
27989323	MCP1	rs12047264	159535626	1	APCS	G	A	0.093	0.019	1.87E-06
27989323	MCP1	rs12073356	208007848	1	LOC148696	G	A	0.143	0.031	4.17E-06
27989323	MCP1	rs12727764	158982477	1	IFI16	G	T	-0.097	0.021	5.02E-06
27989323	MCP1	rs138591554	46289206	3	CCR3	T	A	0.317	0.033	7.94E-22
27989323	MCP1	rs1386930	45884003	3	LZTFL1	C	T	-0.09	0.016	7.86E-09
27989323	MCP1	rs141676607	46653244	3	LOC100132146	C	T	-0.313	0.064	9.48E-07
27989323	MCP1	rs142043796	46679831	3	LOC100132146	G	C	0.241	0.049	8.30E-07
27989323	MCP1	rs145155829	44165646	1	KDM4A	C	T	0.215	0.046	3.72E-06
27989323	MCP1	rs146522229	47798480	19	C5AR1	C	T	0.598	0.118	3.56E-07
27989323	MCP1	rs191688264	30745361	22	SF3A1	C	A	0.483	0.099	1.10E-06
27989323	MCP1	rs2036297	46172903	3	CCR1	G	A	-0.119	0.016	1.09E-13
27989323	MCP1	rs2281300	159156285	1	CADM3	C	T	0.089	0.017	1.71E-07
27989323	MCP1	rs2712431	128316890	3	RPN1	C	A	0.079	0.017	4.76E-06
27989323	MCP1	rs34190208	46736217	3	ALS2CL	C	T	-0.108	0.022	9.91E-07
27989323	MCP1	rs35060576	46434525	3	CCRL2	G	A	-0.096	0.016	3.79E-09
27989323	MCP1	rs35333710	159172854	1	CADM3	G	A	-0.148	0.027	3.71E-08
27989323	MCP1	rs3774641	45937833	3	CCR9	G	T	-0.132	0.019	5.04E-12
27989323	MCP1	rs41338844	46272951	3	CCR3	G	A	-0.22	0.046	3.22E-06
27989323	MCP1	rs4682860	42863804	3	CCBP2	G	A	-0.081	0.016	2.31E-07
27989323	MCP1	rs56212190	42168539	1	HIVEP3	C	T	-0.181	0.037	9.85E-07
27989323	MCP1	rs56300632	4.27E+07	3	NKTR	G	A	-0.077	0.017	3.04E-06
27989323	MCP1	rs62242985	46385638	3	CCR2	G	A	0.101	0.016	5.54E-10

© 2020 Pang et al. *JAMA Cardiology.*

27989323	MCP1	rs7019112	23028336	9	FLJ35282	G	T	0.213	0.047	7.12E-06
27989323	MCP1	rs7197349	78687219	16	WWOX	G	A	-0.097	0.021	2.62E-06
27989323	MCP1	rs7519506	158859138	1	MNDA	C	T	0.099	0.019	2.44E-07
**PMID**	**Protein**	**SNP**	**Position**	**Chr**	**Locus**	**Effect allele**	**Other allele**	**Effect size***	**SE**	***p*-value**
27989323	MCP1	rs75265958	45758020	3	SACM1L	G	T	-0.123	0.027	5.16E-06
27989323	MCP1	rs7527322	159579533	1	APCS	G	A	0.081	0.017	1.54E-06
27989323	MCP1	rs75826707	45908859	3	CCR9	G	A	-0.168	0.037	5.51E-06
27989323	MCP1	rs78629618	46880130	3	PRSS42	C	T	-0.119	0.026	4.23E-06

*Mean difference in the standardised concentration of protein biomarker per allele.
For PMID 31208196, protein SNPs were selected from a meta-analysis of proteomics GWAS in CEU. For each biomarker, common SNPs (MAF>0.01) with a combined *p*-value below 0.01 were retained. Beta coefficients (i.e. effect size) for these SNPs were not reported in this paper so the coefficients were extracted from the INTERVAL study (the largest GWAS for TWEAK: Sun BB, Maranville JC, Peters JE, Stacey D, Staley JR, Blackshaw J, et al. Genomic atlas of the human plasma proteome. *Nature*. 2018(7708);558:73-79).

© 2020 Pang et al. *JAMA Cardiology.*

eTable 13. Selected proteins, data sources, and calculation in meta-analysis and two-sample Mendelian randomisation

Selected proteins	Data sources	Calculation
Analysis 1. MR: BMI and proteins		
7 proteins causally associated with BMI or observationally associated with CVD in CKB and have available GWAS (IL6, IL18, MCP1, MCP3, TRAIL, TWEAK, and HGF)	1) BMI GWAS: GIANT consortium + UKB (0.32 million European individuals), 670 SNPs[17]<br>2) 3 proteomics GWAS[8-11]: Ahola-Olli (48 proteins, 8293 European individuals), Folkersen (83 proteins, 3394 European individuals), Sun (1104 proteins, 3301 European individuals)	For each protein, a conventional inverse-variance weighted (IVW) MR analysis was done by linear regression of the SNP-protein associations on the SNP-BMI associations, with the y-axis intercept forced through the origin. Instruments for BMI are shown in **eFigure 1** and **eTable 4**.
Analysis 2. OB: proteins and CVD		
7 proteins as in *Analysis 1*	Pubmed search: "cardiovascular disease", "coronary heart disease", "myocardial infarction", "stroke", "risk", "IL6", "IL18", "MCP1", "MCP3", "TRAIL", "TWEAK", and "HGF" (restricting to studies in English published in the past 10 years up until March 1, 2019)	A pooled RR of CVD per 1-SD higher protein was calculated for each protein separately using a fixed-effect meta-analysis.
Analysis 3. MR: proteins and CVD		
4 proteins causally associated with BMI or observationally associated with CVD in CKB and had available instruments (no instruments available for IL6 and MCP3)	1) 4 proteomics GWAS[8-11]: Ahola-Olli (48 proteins, 8293 European individuals), Folkersen (83 proteins, 3394 European individuals), Sjaarda (227 proteins, 4147 mostly European individuals), Sun (1104 proteins, 3301 European individuals)<br>2) CVD GWAS[14,16]: CARDIoGRAMplusC4D (60,801 cases and 123,504 controls) and MEGASTROKE (67,162 cases and 454,450 controls)	For each protein, a conventional IVW MR analysis was done by logistic regression of the SNP-disease associations on the SNP-protein associations, with the y-axis intercept forced through the origin. Instruments for proteins are shown in **eTable 12**. Separate analysis was done for CHD and ischaemic stroke.

Analysis 2: Inclusion criteria were prospective cohort studies, nested case-control studies, or case-subcohort studies reporting the association between plasma proteins and CVD incidence or mortality. Bibliographies of included studies and related reviews were manually searched for additional eligible articles. No observational studies were identified for TRAIL and TWEAK.
Analysis 3: Two proteins (IL6 and MCP3) were not included in the two-sample Mendelian randomisation analysis because no instruments were available. In the main analysis, SNPs were included if they were in *cis* loci and were not in linkage disequilibrium. In the sensitivity analysis, SNPs on *trans* loci were also included.

© 2020 Pang et al. *JAMA Cardiology.*
